# Supplementary material for: Consistent Estimation in Mendelian Randomization with Some Invalid Instruments Using a Weighted Median Estimator
Source: Genet Epidemiol. 2016 Apr 7;40(4):304–14. doi: 10.1002/gepi.21965 (PMC4849733; doi:10.1002/gepi.21965)
Supplement: Supplementary file 1 — Supporting Information S1 [file GEPI-40-304-s001.docx]

\documentclass[a4paper,12pt]{article} % Figure 1 change colour

\usepackage{color}

\usepackage{epsfig}

\usepackage{amsfonts}

\usepackage{amsmath}

\usepackage{amssymb, amsbsy}

\usepackage{mathtools}

\usepackage{chngpage}

\usepackage{rotating}

\usepackage{multirow}

\usepackage{setspace}

\usepackage{bbding, pifont}

\usepackage{url}

\usepackage{bibunits}

\usepackage{chngpage}

\usepackage{setspace}

\usepackage[square, authoryear]{natbib}

\usepackage{array}

\usepackage{caption}

\hyphenation{MR-Egger}

\newcommand{\tick}{\ding{51}}

\newcommand{\cross}{\ding{55}}

\renewcommand{\thempfootnote}{\arabic{mpfootnote}}

\newcommand\blfootnote[1]{%

\begingroup

\renewcommand\thempfootnote{}\footnote{#1}%

\addtocounter{mpfootnote}{-1}%

\endgroup

}

\DeclareMathOperator{\se}{se}

\DeclareMathOperator{\var}{var}

\newcommand{\slfrac}[2]{\left.#1\middle/#2\right.}

\addtolength{\hoffset}{-1.4cm}

\addtolength{\textwidth}{2.8cm}

\addtolength{\voffset}{-1.5cm}

\addtolength{\textheight}{3cm}

\begin{document}

\makeatletter

\renewcommand\@biblabel[1]{}

\makeatother

\title{Consistent estimation in Mendelian randomization with some invalid instruments using a weighted median estimator}

\author{}%\author{Jack Bowden \and \and Stephen Burgess} \thanks{Address: Strangeways Research Laboratory, 2 Worts Causeway, Cambridge, CB1 8RN, UK. Telephone: +44 1223 748651. Fax: +44 1223 748658. Correspondence to: sb452@medschl.cam.ac.uk.}

\author{Jack Bowden\\ \small{Integrative Epidemiology Unit, University of Bristol} \\ \and George Davey Smith\\ \small{Integrative Epidemiology Unit, University of Bristol} \\ \and Philip C.\ Haycock\\ \small{Integrative Epidemiology Unit, University of Bristol} \\ \and Stephen Burgess\thanks{Corresponding author. Address: Department of Public Health \& Primary Care, Strangeways Research Laboratory, 2 Worts Causeway, Cambridge, CB1 8RN, UK. Telephone: +44 1223 740002. Correspondence to: sb452@medschl.cam.ac.uk.}\\ \small{Department of Public Health and Primary Care, University of Cambridge}}

\date{}

\maketitle

\renewcommand{\thetable}{\Roman{table}}

%\defcitealias{ehret2011genepi}{Ehret et al., 2011}

\noindent \noindent \textbf{Running title:} Mendelian randomization with some invalid instruments [48 chars].

\clearpage

\setstretch{1.54}

\begin{bibunit}[apalike]

\subsection*{Abstract}

Developments in genome-wide association studies and the increasing availability of summary genetic association data have made application of Mendelian randomization relatively straightforward. However, obtaining reliable results from a Mendelian randomization investigation remains problematic, as the conventional inverse-variance weighted method only gives consistent estimates if all of the genetic variants in the analysis are valid instrumental variables. We present a novel weighted median estimator for combining data on multiple genetic variants into a single causal estimate. This estimator is consistent even when up to 50\% of the information comes from invalid instrumental variables. In a simulation analysis, it is shown to have better finite-sample Type 1 error rates than the inverse-variance weighted method, and is complementary to the recently proposed MR-Egger (Mendelian randomization-Egger) regression method. In analyses of the causal effects of low-density lipoprotein cholesterol and high-density lipoprotein cholesterol on coronary artery disease risk, the inverse-variance weighted method suggests a causal effect of both lipid fractions, whereas the weighted median and MR-Egger regression methods suggest a null effect of high-density lipoprotein cholesterol that corresponds with the experimental evidence. Both median-based and MR-Egger regression methods should be considered as sensitivity analyses for Mendelian randomization investigations with multiple genetic variants.

\vspace{2mm}

\noindent \noindent \textbf{Key words:} Mendelian randomization, instrumental variables, robust statistics, Egger regression, pleiotropy.

%\vspace{2mm}

%\noindent \noindent \textbf{Abbreviations:} CAD, coronary artery disease; GLGC, Global Lipids Genetics Consortium; \MBOX{HDL-C}, high-density lipoprotein cholesterol; InSIDE, instrument strength independent of direct effect; IVW, inverse-variance weighted; IV, instrumental variable; \MBOX{LDL-C}, low-density lipoprotein cholesterol; MR, Mendelian randomization.

\clearpage

\setstretch{1.32}

\section*{Introduction}

Over the past decade, Mendelian randomization has become an established tool for probing questions of causality when characterizing the aetiology of disease \citep{daveysmith2003, burgess2015book}. The requirement for such approaches stems from a fundamental limitation of observational data, namely that causation cannot automatically be inferred from an association between an exposure and a disease. The association could be due to unobserved confounding between the exposure and the outcome, or reverse causation (the outcome affects the exposure) \citep{daveysmith2004}. These limitations are generally of no consequence when the aim is merely to predict the likelihood of future outcomes. However, if an exposure has a non-causal association with an outcome, then public health or pharmaceutical interventions targeted at the exposure will realize no material benefit and represent a waste of resources.

The basic premise of Mendelian randomization relies on genetic variants that explain variation in the exposure, but do not affect the disease outcome except possibly through the exposure. Such genetic variants are known as instrumental variables (IVs) \citep{greenland2000}. Subgroups of individuals with differing numbers of alleles of a genetic IV can be thought of as having been randomized to receive a different mean level of the exposure during their life course \citep{daveysmith2005a, nitsch2006}. If the randomization is indeed uncontaminated (in the sense that a person's genetic subgroup is independent of all factors, except the exposure and any causal consequence of the exposure), then differences in the outcome between genetic subgroups can be causally attributed to the exposure \citep{didelez2007}. The following three assumptions are necessary for a genetic variant to be a valid IV \citep{martens2006}:

\begin{itemize}

\item IV1: the variant is predictive of the exposure;

\item IV2: the variant is independent of any confounding factors of the exposure--outcome association;

\item IV3: the variant is conditionally independent of the outcome given the exposure and the confounding factors.

\end{itemize}

The IV assumptions are illustrated in Figure~\ref{dag}. IV1 is the only assumption that can be fully empirically tested, since IV2 and IV3 depend on all possible confounders of the exposure--outcome association, both measured and unmeasured. Any statistical method for obtaining causal inferences must by necessity make an untestable assumption. The validity of a causal conclusion from a Mendelian randomization analysis depends on the plausibility of these assumptions.

\begin{figure}[htbp]

\begin{center}

\includegraphics[width=10cm]{wmedian_figs/Figure1R}

\end{center}

\caption{\onehalfspacing Illustrative diagram representing the hypothesized relationships between genetic variant $G_j$, exposure $X$, disease $Y$ and confounders $U$ when $G_j$ is a valid instrumental variable (IV). Crosses indicate violations of assumptions IV2 and IV3 that potentially lead to invalid inferences from conventional methods.} \label{dag}

\end{figure}

Early implementations of the Mendelian randomization approach were largely constrained by limitations of power as investigations were undertaken in small sample sizes, and used only a handful of genetic variants (each explaining a small proportion of the variance in the exposure). However, a revolution in the field is under way led by the identification of increasing numbers of genetic variants robustly associated with particular traits, and the public release by many large consortia of summary association estimates for hundreds of thousands of genetic variants with exposures and disease outcomes \citep{burgess2014twosample}, such as the Global Lipids Genetics Consortium (GLGC) for lipid fractions \citep{willer2013} and the CARDIoGRAM consortium for coronary artery disease risk \citep{deloukas2013}. The availability of such summary data has facilitated powerful Mendelian randomization investigations to be conducted in a two-sample framework by providing genetic associations precisely estimated in large sample sizes \citep{burgess2013genepi}.

The inclusion of multiple variants in a Mendelian randomization analysis typically leads to increased statistical power \citep{freeman2013}, but presents new challenges \citep{glymour2012}. Firstly, if there is substantial overlap in the datasets from which the association estimates with the exposure and with the outcome were obtained, then the resulting analysis suffers from bias and inflated type 1 error rates when the included variants are `weak' (that is, they do not explain a substantial proportion of variation in the exposure in the dataset under analysis) \citep{burgess2010avoiding, pierce2013}. Secondly, it may not be the case that all included genetic variants are valid IVs. % Selecting variants that achieve genome-wide significance and/or combining genetic variants into a single allele score can remedy this issue. However, this does not address the issue of invalid IVs. Furthermore, if a gene-score is automatically constructed from many variants, this can remove the opportunity to detect and adjust for invalid instruments in the analysis (Bowden, Smith, and Burgess 2015).

In this paper we propose a new method for Mendelian randomization using summary data that offers protection against invalid instruments: the weighted median estimator. This approach can provide a consistent estimate of the causal effect even when up to 50\% of the information contributing to the analysis comes from genetic variants that are invalid IVs. We explore the statistical properties of the weighted median estimator in a realistic simulation study, and compare with an alternative summary data analysis method also robust to some violations of the IV assumptions, MR-Egger regression \citep{bowden2015}. We explain how the two approaches differ in their assumptions, and when they each work well or fail. % , and how, if used together, they can provide an authoritative basis for probing the sensitivity of an MR-analysis to violations of the IV assumptions

We provide an illustrative estimate of two-sample Mendelian randomization using summary data on the associations of 185 genetic variants with high-density lipoprotein cholesterol, low-density lipoprotein cholesterol, and triglycerides from the GLGC, and with coronary artery disease risk from the \mbox{CARDIoGRAM} consortium. We conclude with a discussion of the issues raised and the potential for future research. % is median really better for weak instruments??

\section*{Methods}

Consider data from a Mendelian randomization study on $J$ genetic variants $G_1, \ldots, G_J$, a continuous exposure $X$ and a continuous outcome $Y$. All confounding variables are subsumed into a single variable $U$. We initially assume that all genetic variants are valid IVs, and further assume that all the relationships between variables in Figure~\ref{dag} are linear without heterogeneity or effect modification:

\begin{align}

X|G_j &= \gamma_0 + \gamma_j G_j + \epsilon_{Xj} \notag \\

Y|G_j &= \Gamma_0 + \Gamma_j G_j + \epsilon_{Yj}. \notag

\end{align}

Assumption IV1 tells us that all variants are associated with the exposure, so $\gamma_j \neq 0$ for all $j$. Assumptions IV2 and IV3 tell us that the genetic associations with the outcome $\Gamma_j$ are equal to the genetic associations with the exposure $\gamma_j$ multiplied by the causal effect of the exposure on the outcome $\beta$: so $\Gamma_j = \beta \gamma_j$. The error terms $\epsilon_{Xj}$ and $\epsilon_{Yj}$ are assumed to be normally distributed and contain contributions from the confounder $U$ and all genetic variants except $G_j$. In a one-sample setting, the exposure and outcome data are collected on the same individuals, in which case $\epsilon_{Xj}$ and $\epsilon_{Yj}$ are correlated. If exposure and outcome data are collected on different sets of individuals (known as two-sample Mendelian randomization \citep{pierce2013}), then these error terms are independent. We assume throughout this manuscript that all genetic variants are uncorrelated (that is, not in linkage disequilibrium), so that the information provided by each genetic variant is independent. Extensions to allow for correlated variants in a straightforward application of Mendelian randomization have been developed \citep{burgess2013genepi}, but the situation of uncorrelated variants is usual in applied practice.

\subsection*{Inverse-variance weighted method}

The causal effect of the exposure on the outcome can be estimated using the $j$th variant as the ratio of the gene--outcome association and the gene--exposure association estimates \citep{lawlor2007}:

\begin{equation}

\hat{\beta}_j = \frac{\hat{\Gamma}_j}{\hat{\gamma}_j}. \notag

\end{equation}

If the IV assumptions are satisfied for genetic variant $j$, then $\Gamma_j = \beta \gamma_j$ and the ratio estimate is consistent asymptotically. Furthermore, if the genetic variants are uncorrelated (not in linkage disequilibrium) then the ratio estimates from each genetic variant can be combined into an overall estimate using a formula from the meta-analysis literature \citep{johnson2013vig}:

\begin{equation}

\hat{\beta}_{IVW} = \frac{\sum_j \hat{\gamma}_j^2 \sigma_{Yj}^{-2} \hat{\beta}_j}{\sum_j \hat{\gamma}_j^2 \sigma_{Yj}^{-2}} \notag

\end{equation}

where $\sigma_{Yj}$ is the standard error of the gene--outcome association estimate for variant $j$. This is referred to as the inverse variance weighted (IVW) estimator \citep{burgess2013genepi}. Provided that the genetic variants are uncorrelated, the IVW estimate is asymptotically equal to the two-stage least squares estimate commonly used with individual-level data. If all genetic variants satisfy the IV assumptions, then the IVW estimate is a consistent estimate of the causal effect (that is, it converges to the true value as the sample size increases), as it is a weighted mean of the individual ratio estimates.

\subsection*{Simple median estimator}

The IVW estimate is an efficient analysis method when all genetic variants are valid IVs. Unfortunately, it will be biased even if only one genetic variant is invalid. For this reason, the IVW estimate can be said to have a 0\% breakdown level. However, an estimator exists that enjoys a 50\% breakdown level; that is, it provides a consistent estimate of the causal effect when up to (but not including) 50\% of genetic variants are invalid. This simple estimator is the median ratio estimate \citep{han2008}. Specifically, let $\hat{\beta}_{j}$ denote the $j$th ordered ratio estimate (arranged from smallest to largest). If the total number of genetic variants is odd ($J = 2k+1$), the simple median estimator is the middle ratio estimate $\hat{\beta}_{k+1}$. If it is even ($J = 2k$), the median is interpolated between the two middle estimates $\frac{1}{2} (\hat{\beta}_{k} + \hat{\beta}_{k+1})$. In terms of notation, we assume in this manuscript that genetic variants are ordered according to their ratio estimates.

In order to understand why the median estimator achieves a 50\% breakdown level, we consider a fictional analysis using ten genetic variants, six of which are valid IVs and four of which are invalid. Figure~\ref{illustrate} (left) shows a scatter plot of 10 gene--exposure ($\hat{\gamma}_j$) and gene--outcome ($\hat{\Gamma}_j$) association estimates for an Mendelian randomization study with a finite sample size \citep{kang2014}. The ratio estimate for each genetic variant is the gradient of the line connecting the relevant datapoint for that variant to the origin. The true causal effect is shown by the dotted black line, the median estimate by the dashed line, and the IVW estimate by the solid line. Estimates from valid IVs are shown by hollow circles, estimates from invalid IVs are shown by solid circles. Although the valid IVs follow the true slope, the IVW estimate is pulled away from the true value by the invalid instruments, which yield biased estimates of the causal effect. Figure~\ref{illustrate} (right) shows the same scatter plot for an infinite sample size. Now the six valid instruments lie perfectly on the true line, and all yield the same true causal estimate. The median ratio estimate (in this case, an average of the 5th and 6th ratio estimates) is the true causal effect. In contrast, the IVW estimate remains biased even with infinite data, as the ratio estimate from each genetic variant always contributes towards the overall IVW estimate.

\begin{figure}[hbtp]

\centering

\includegraphics[width=0.49\textwidth, clip]{wmedian_figs/Figure2Left}

\includegraphics[width=0.49\textwidth, clip]{wmedian_figs/Figure2Right}

\caption{\onehalfspacing \label{illustrate} Fictional example of a Mendelian randomization analysis with 10 genetic variants -- 6 valid instrumental variables (hollow circles) and 4 invalid instrumental variables (solid circles) for finite sample size (left) and infinite sample size (right) showing inverse variance weighted (IVW, solid line) and simple median (dashed line) estimates compared with the true causal effect (dotted line). The ratio estimate for each genetic variant is the gradient of the line connecting the relevant datapoint for that variant to the origin; the simple median estimate is the median of these ratio estimates.}

\end{figure}

\subsection*{Weighted median estimator}

The simple median estimator is inefficient, especially when the precision of the individual estimates varies considerably. In order to account for this, a weighted median can be defined as follows. Let $w_j$ be the weight given to the $j$th ordered ratio estimate, and let $s_j = \sum_{k=1}^j w_k$ be the sum of weights up to and including the weight of the $j$th ordered ratio estimate. Weights are standardized, so that the sum of the weights $s_J$ is 1. The weighted median estimator is the median of a distribution having estimate $\hat{\beta}_j$ as its $p_j = 100 (s_j-\frac{w_j}{2})$th percentile. For all other percentile values, we extrapolate linearly between the neighboring ratio estimates. The contribution of the $j$th genetic variant to the empirical distribution is proportional to its weight $w_j$. The simple median estimator can be thought of as a weighted median estimator with equal weights. While the simple median provides a consistent estimate of causal effect if at least 50\% of IVs are valid, the weighted median will provide a consistent estimate if at least 50\% of the weight comes from valid IVs. We assume that no single IV contributes more than 50\% of the weight, otherwise the 50\% validity assumption is equivalent to assuming that this IV is valid (in which case, an analysis should simply be based on this one IV). Some technical remarks on weighted medians are given in Web Appendix 1.

As an illustration, two sets of weights are given in Table~\ref{illustweights}, and percentiles are calculated for each set of weights as well as for the simple median (equal weights). As the first set of weights are symmetric, the weighted median in this case equals the simple median. However, less weight is given to outlying estimates, and the empirical distribution function (Figure~\ref{illustdist}, red line) is close to the median value across a wider range of the distribution. Confidence intervals for the weighted median, which can be obtained by a parametric bootstrap method, should therefore be narrower. In the second set of weights, smaller estimates happen to receive more weight (Figure~\ref{illustdist}, blue line). The weighted median estimate will be interpolated between ratio estimates $\hat{\beta}_3$ and $\hat{\beta}_4$, but will be closer to $\hat{\beta}_4$ as the percentile $p_4$ is closest to 50\%. The exact weighted median estimate in this case will be:

\begin{equation}

\hat{\beta}_{WM} = \hat{\beta}_3 + (\hat{\beta}_4 - \hat{\beta}_3) \times \frac{50 - 27.78}{52.78 - 27.78} \notag % = 0.111 \hat{\beta}_3 + 0.889 \hat{\beta}_4

\end{equation}

The weighted median can also be thought of as the simple median from a set of values (a pseudo-population) in which the ratio estimate $\hat{\beta}_1$ for variant $1$ appears $100 \times w_1$ times, ratio estimate $\hat{\beta}_2$ for variant $2$ appears $100 \times w_2$ times, and so on. R code to calculate weighted median estimates, confidence intervals, standard errors and p-values is provided in Web Appendix 2.

\begin{center}[Table~\ref{illustweights} should appear about here]\end{center}

\begin{figure}[htbp]

\begin{center}

\includegraphics[width=12cm]{wmedian_figs/Figure3}

\end{center}

\caption{\onehalfspacing Empirical distribution functions of ordered ratio instrumental variable estimates ($\hat{\beta}_j$) used for calculation of the simple median estimate (black) and two weighted median estimates (shown in red and blue) using the weights given in Table~\ref{illustweights}.} \label{illustdist}

\end{figure}

Analogously to the IVW method, we suggest using the inverse of the variance of the ratio estimates as weights:

\begin{equation}

w'_j = \frac{\hat{\gamma}_j^2}{\sigma_{Yj}^2} \notag

\end{equation}

These weights are derived from the delta method for the variance of the ratio of two random variables, and represent the reciprocal of the variance of the ratio estimates (the inverse-variance weights) \citep{thomas2007}. Standardized weights are $w_j = \frac{w'_j}{\sum_j w'_j}$. The unstandardized weights are identical to those used in the IVW estimator. Only the first order term from the delta expansion is used here; further terms could be considered, although we found that they did not affect estimates or standard errors from the weighted median method substantially.

\subsection*{Penalized weighted median estimator}

In Figure~\ref{illustrate} (left side), although the invalid IVs do not contribute directly to the median estimate, they do influence it. The simple median estimate in this case is an average of the 5th and 6th ratio estimates. If the invalid IVs were not present, the median estimate would be the average of the 3rd and 4th ratio estimates. The presence of invalid instruments does not affect the median estimate asymptotically, but in this example it will bias the estimate in finite samples, as the 5th and 6th ratio estimates will always be larger than the 3rd and 4th ratio estimates. This is likely to be a problem when the estimates from invalid IVs are not balanced about the true causal effect (as in this example, all 4 invalid estimates are greater than the true causal effect).

One way of minimizing this problem is downweighting the contribution to the analysis of genetic variants with heterogeneous ratio estimates. Heterogeneity between estimates can be quantified by Cochran's Q statistic:

\begin{equation}

Q = \sum_j Q_j = \sum_j w'_j (\hat{\beta}_j - \hat{\beta})^2 \notag

\end{equation}

where we take $\hat{\beta}$ to be the IVW estimate \citep{greco2015}. The Q statistic has a chi-squared distribution on $J-1$ degrees of freedom under the null hypothesis that all genetic variants are valid IVs and the same causal effect is identified by all variants. Under this null hypothesis, the components of the Q statistic corresponding to the individual genetic variants ($Q_j$) approximately have chi-squared distributions with 1 degree of freedom. So as not to distort the weightings of the majority of variants, we propose penalization using the one-sided upper p-value (denoted $q_j$) on a chi-squared-1 distribution corresponding to $Q_j$, by multiplying the weight by the p-value multiplied by 20 (or by 1 if the p-value is greater than 0.05). The (unstandardized) penalized weights ($w^{*}_j$) are therefore:

\begin{equation}

w^{*}_j = w'_j \times \min(1, 20 q_j) \notag

\end{equation}

This means that most variants will be unaffected by the penalization, but outlying variants will be severely downweighted.

\subsection*{MR-Egger regression}

An alternative robust method for Mendelian randomization with summary data has been recently proposed by Bowden et al.\ \citep{bowden2015}, referred to as `MR-Egger regression'. This approach was motivated from a method in the meta-analysis literature for the assessment of small-study bias (often called ``publication bias'') \citep{egger1997}. This performs a weighted linear regression of the gene--outcome coefficients $\hat{\Gamma}_j$ on the gene--exposure coefficients $\hat{\gamma}_j$:

\begin{equation}

\hat{\Gamma}_j = \beta_{0E} + \beta_{E} \hat{\gamma}_j \notag

\end{equation}

in which all the $\hat{\gamma}_j$ associations are orientated to be positive (the orientation of the $\hat{\Gamma}_j$ associations should be altered if necessary to match the orientation of the $\hat{\gamma}_j$ parameters), and the weights in the regression are the inverse-variances of the gene--outcome associations ($\sigma_{Yj}^{-2}$). Re-orientation of the variants is performed as the orientation of genetic variants is arbitrary (that is, estimates can be presented with reference to either the major or minor allele), and different orientations of genetic variants change the estimate of the intercept, as well as the sign and magnitude of the pleiotropic effect of the genetic variant. If there is no intercept term in the regression model, then the MR-Egger slope estimate $\hat{\beta}_E$ will equal the IVW estimate \citep{burgess2014pleioajeappendix}.

The value of the intercept term $\hat{\beta}_{0E}$ can be interpreted as an estimate of the average pleiotropic effect across the genetic variants \citep{bowden2015}. The pleiotropic effect is the effect of the genetic variant on the outcome that is not mediated via the exposure. An intercept term that differs from zero is indicative of overall directional pleiotropy; that is, pleiotropic effects do not cancel out and the IVW estimate is biased.

MR-Egger regression additionally provides an estimate for the true causal effect $\hat{\beta}_{E}$ that is consistent even if all genetic variants are invalid due to violation of IV3, but under a weaker assumption known as the InSIDE (instrument strength independent of direct effect) assumption. If the association of the $j$th genetic variant with the outcome $\Gamma_j = \beta \gamma_j + \alpha_j$, where $\alpha_j$ is the pleiotropic (direct) effect of the variant, then the InSIDE assumption states that the pleiotropic effects $\alpha_j$ must be distributed independently of the instrument strength parameters $\gamma_j$ \citep{kolesar2014}. (Formally, the consistency property holds both as the sample size and the number of instruments increases. For a fixed number of instruments, consistency only holds asymptotically if the correlation between the $\alpha_j$ and $\gamma_j$ parameters is zero.) The InSIDE assumption is likely to be satisfied if pleiotropic effects on the outcome are direct (that is, not via a confounder). There is some empirical evidence supporting the proposition that genetic effects on separate exposures are independent \citep{pickrell2015a}. However, if the pleiotropic effects of genetic variants are all via a single confounder, then they will be correlated with instrument strength, and the InSIDE assumption will be violated.

\section*{Simulation study}

In order to investigate the performance of the weighted median method in realistic settings, as well as to determine in what scenarios it performs well or badly in comparison with the IVW and MR-Egger regression methods, we perform a simulation study. We assume there are 25 genetic variants that are candidate IVs, and consider three scenarios:

\begin{itemize}

\item[1.] balanced pleiotropy, InSIDE assumption satisfied -- pleiotropic effects are equally likely to be positive as negative, these effects are uncorrelated with the instrument strength;

\item[2.] directional pleiotropy, InSIDE assumption satisfied -- only positive pleiotropic effects are simulated, these effects are uncorrelated with the instrument strength;

\item[3.] directional pleiotropy, InSIDE assumption not satisfied -- pleiotropic effects are via a confounder, these effects on the outcome are therefore positive and are correlated with the instrument strength.

\end{itemize}

The status of a genetic variant as an invalid IV is determined by a random draw for each variant. The probability of being an invalid variant is taken as 0.1, 0.2, and 0.3. We consider cases with 10\thinspace000 and 20\thinspace000 participants.

We generated 10\thinspace000 simulated datasets for each scenario in a two-sample setting with two values of the causal effect ($\beta = 0$, null causal effect; $\beta = 0.1$, positive causal effect); the simulations are repeated in Web Appendix 3 in a one-sample setting. Only the summary data on the genetic associations with the exposure and with the outcome (and their standard errors) are used as data inputs in the analysis methods (individual-level data are not used). Details of the data-generating model and the parameters used in the simulation study are given in Web Appendix 3.

\subsection*{Simulation results}

The simulation results in the two-sample setting are given in Table~\ref{tab:SimScenario1} (null causal effect) and Table~\ref{tab:SimScenario2} (positive causal effect). In Scenario 1 (balanced pleiotropy), the methods all give close to unbiased causal estimates, and have reasonable Type 1 error rates (power under the null is equal to Type 1 error). However, the power of the estimates with a positive causal effect differs substantially. The weighted median methods have lower mean standard errors than the IVW method, and generally have greater power with a positive causal effect (although not uniformly so), particularly as the proportion of invalid IVs increases. This is because invalid IVs do not influence the median estimates directly. Although Type 1 error rates from MR-Egger regression are at nominal levels, estimates from the MR-Egger method are considerably less precise (mean standard errors are around 3 times larger than IVW standard errors), and power to detect a causal effect is considerably reduced. Precision in the MR-Egger method depends on the genetic variants having different associations with the exposure; if all genetic variants had the same magnitude of association with the exposure, then the MR-Egger regression estimate would not be identified.

In Scenario 2 (directional pleiotropy, InSIDE assumption satisfied), estimates from the IVW method are biased with inflated Type 1 error rates. Estimates from the weighted median methods are less biased, although there is a consistent bias in the direction of the pleiotropic variants. Nominal Type 1 error rates are maintained when only 10\% of genetic variants are invalid IVs, although Type 1 error rates are above the expected 5\% rate when 20\% or more genetic variants are invalid IVs. However, even though they are inflated, Type 1 error rates are far lower for the median-based methods than those from the IVW method. The penalized weighted median estimates are less biased when a few genetic variants are invalid, but when 30\% of genetic variants are invalid, it seems that the invalid IVs are often retained in the analysis (as their estimates are homogeneous with each other) and valid IVs are excluded. Estimates from MR-Egger regression are close to unbiased, and Type 1 error rates are at nominal levels, suggesting its utility as a sensitivity analysis method when the InSIDE assumption is satisfied. However, the wide confidence intervals and low power with a true causal effect (close to 5\%) mean that MR-Egger regression is not likely to be a discerning sensitivity analysis, but rather giving conservative findings as to whether a causal effect is present or not. Although power to detect a causal effect is greater in the IVW method, this is achieved at the cost of the Type 1 error rate; comparisons of power that do not make reference to the Type 1 error rate are meaningless.

In Scenario 3 (directional pleiotropy, InSIDE assumption not satisfied), all methods suffer from bias and inflated Type 1 error rates. Most concerning are results from the MR-Egger method, which are more biased than those from the IVW method and have similar Type 1 error rates. The weighted median methods, and in particular the penalized weighted median method, give lower Type 1 error rates, suggesting their potential use as a sensitivity analysis method when InSIDE is not satisfied, as well as when it is satisfied.

In Web Tables~\ref{tab:SimScenario1a} and \ref{tab:SimScenario2a}, results are presented in a one-sample setting. Each of the methods suffers from weak instrument bias in this setting, and Type 1 error rates are inflated. Estimates from MR-Egger regression are substantially more biased than those from other methods. However, the median-based methods remain a reasonable sensitivity analysis, as Type 1 error rates are similar to those of the IVW method in Scenario 1, and substantially lower in Scenarios 2 and 3. In Web Table~\ref{tab:SimScenario1c}, results are presented in a two-sample setting for the simple median estimator, and a weighted median estimator using inverse-standard error weights rather than the inverse-variance weights used above. Simple median estimates are slightly less precise than those from the weighted median methods, and have similar bias and Type 1 error properties in Scenarios 1 and 2, but much improved Type 1 error rates in Scenario 3. This is because the invalid variants in this scenario are stronger than the valid variants, and so receive more weight in the weighted analyses. It is unclear that this would occur in applied practice, although it suggests that the simple median method is a worthwhile additional sensitivity analysis. The inverse-standard error weighted median estimator has improved Type 1 error properties compared with the inverse-variance weighted median estimator particularly in Scenario 3 (although not uniformly), indicating the potential to use different choices of weights in median-based methods to provide further sensitivity analyses.

\begin{center}[Tables~\ref{tab:SimScenario1} and \ref{tab:SimScenario2} should appear around here]\end{center}

\section*{Example: lipid concentrations and coronary artery disease risk}

Low-density lipoprotein cholesterol (\mbox{LDL-c}) is observationally positively associated with coronary artery disease (CAD) risk \citep{diangel2009}. Evidence from randomized trials of pharmaceutical interventions to lower \mbox{LDL-c} concentrations (such as statins \citep{pedersen1994, cheung2004}), and from Mendelian randomization \citep{linsel2008, do2013} suggests that this association is reflective of a causal effect of \mbox{LDL-c} on CAD risk. On the contrary, although high-density lipoprotein cholesterol (\mbox{HDL-c}) is observationally inversely associated with CAD risk, interventions to raise \mbox{HDL-c} concentrations \citep{schwartz2012} and focused Mendelian randomization investigations \citep{voight2012} suggest that there is not even a moderate causal effect of \mbox{HDL-c} on CAD risk. However, a more liberal Mendelian randomization analysis including genetic variants associated with other lipid fractions (in particular, triglycerides) did suggest a causal effect of \mbox{HDL-c} on CAD risk \citep{holmes2014}. As a proof of concept example, we use summary data from the GLGC on genetic associations with lipid fractions, and from CARDIoGRAM on associations with CAD risk, and perform the analysis methods discussed in this paper to see whether the causal effect of \mbox{LDL-c} is preserved by the weighted median and MR-Egger regression methods, as well as whether the spurious causal effect of \mbox{HDL-c} is contradicted by the robust methods. We use data provided by Do et al.\ \citep{do2013} on 185 genetic variants for this analysis. The genetic associations with the lipid fractions are in standard deviation units, and with the outcome are log odds ratios, so the causal effects represent log odds ratios per 1 standard deviation increase in the lipid fraction. Further details of the analysis are provided in Web Appendix 4.

Taking each of \mbox{LDL-c}, \mbox{HDL-c}, and additionally triglycerides as the exposure variable in turn, we consider two analysis strategies. First, we take all genetic variants associated with the exposure at a genome-wide level of significance (taken as $p<10^{-8}$). Second, we restrict to genetic variants for which the p-value for association with the target exposure (say, \mbox{HDL-c}) is less than the p-values for association with the non-target exposures (say, \mbox{LDL-c} and triglycerides). We perform each of the methods presented in the manuscript (IVW, simple median, weighted median, penalized weighted median, MR-Egger regression). The data are presented as scatter plots in Figure~\ref{scatters} and Web Figure~\ref{scatters2}, and as funnel plots in Web Figure~\ref{funnels}.

\begin{figure}[htbp]

\begin{center}

\includegraphics[width=0.98\textwidth]{wmedian_figs/scattersR}

\end{center}

\caption{\onehalfspacing Scatter plots of genetic associations with the outcome (coronary artery disease risk, CAD) against genetic associations with the exposure (low-density lipoprotein cholesterol, \mbox{LDL-c}; high-density lipoprotein cholesterol, \mbox{HDL-c}; triglycerides). Left side: all genetic variants, right side: genetic variants having primary association with the target exposure. Solid line represents IVW estimate, dashed line represents weighted median estimate, and dotted line represents MR-Egger estimate.} \label{scatters}

\end{figure}

\begin{center}[Table~\ref{exampletab} should appear about here]\end{center}

Results are given in Table~\ref{exampletab}. The causal effects of \mbox{LDL-c} and triglycerides are robustly detected by all the analysis methods. In contrast for \mbox{HDL-c}, both MR-Egger regression and the weighted median methods suggest that the `causal effect' of \mbox{HDL-c} on CAD risk detected by the IVW method is suspect. With the exception of the simple median estimates, all other robust analysis methods give estimates compatible with the null. The test for directional pleiotropy in the MR-Egger regression method suggested pleiotropy for \mbox{HDL-c}, but not for \mbox{LDL-c} or triglycerides. Although similar results for \mbox{HDL-c} can be obtained by filtering out genetic variants that are suspected to have pleiotropic effects, by omitting genetic variants there is a loss of power (for example, in Holmes et al.\ a liberal genetic risk score explained 3.8\% of the variance in \mbox{HDL-c}, whereas a conservative score omitting potentially pleiotropic variants explained only 0.3\%).

By way of comparison between the robust methods, similar results were seen in this example as in the simulation study. The median-based methods were consistently and substantially more precise than the MR-Egger regression method, with standard errors reduced by around 30-50\%. The precision of the weighted median methods, in particular the penalized weighted median method, was not much worse than that of the IVW method, and in some cases was slightly better. The simple median method was not as impressive, suggesting a causal effect of \mbox{HDL-c} on CAD risk, and giving less precise estimates than those from the weighted median methods. The MR-Egger regression method performed well despite doubts about the InSIDE assumption in the case of \mbox{HDL-c}; many variants associated with \mbox{HDL-c} are also associated with \mbox{LDL-c} and triglycerides, and these associations are approximately proportional (see Web Figure~\ref{scatters2}), hence pleiotropic effects on CAD risk may operate via \mbox{LDL-c} and triglycerides. This may be the reason why the MR-Egger estimate changed sign in the analysis using variants having primary association with \mbox{HDL-c}.

%The wider conclusion from this example is that naive Mendelian randomization analyses for \mbox{LDL-c}, \mbox{HDL-c}, and triglycerides gave results in line with the observational associations of the lipid fractions, suggesting a protective causal effect of \mbox{HDL-c} that is not supported by experimental evidence. In contrast, the median-based and Egger regression analyses gave results in line with the experimental evidence, and in line with more principled Mendelian randomization investigations \citep{voight2012, holmes2014}. An advantage of the weighted median and penalized weighted median methods is that precision of the causal estimate is not lost, either by excluding potentially pleiotropic variants (as in a more principled analysis), or by using Egger regression.

\section*{Discussion}

In this paper, we have introduced a weighted median method for the estimation of a causal effect using multiple instrumental variables. Unlike other methods commonly used in Mendelian randomization, this method can give consistent estimates when some of the genetic variants in the analysis are not valid IVs. We have shown how the method performs in a simulation study and in an applied example.

A summary of the median-based and other methods considered in this paper is given in Table~\ref{summresults}. Of particular interest is the comparison between the weighted median approach and MR-Egger regression. MR-Egger regression can give consistent estimates when 100\% of genetic variants are invalid IVs, whereas the weighted median method requires 50\% of the weight to come from valid IVs. However, the weighted median approach allows the IV assumptions to be violated in a more general way for the invalid IVs, whereas MR-Egger regression replaces one set of untestable assumptions (IV2 and IV3) with a weaker, but still untestable assumption (the InSIDE assumption). Additionally, weighted median estimates were substantially more precise than those from MR-Egger regression in the simulation study and applied example. MR-Egger regression estimates are likely to be particularly imprecise if all genetic variants have similar magnitudes of association with the exposure.

Although the only mechanism for generating invalid IVs considered in this paper was pleiotropy, median-based methods are agnostic to the mechanism by which the invalid IVs violate the IV assumptions. Consistent estimates would be guaranteed if some genetic variants were invalid IVs due to other mechanisms, such as linkage disequilibrium, population stratification, or differential survival \citep{bochud2008}. One potential source of bias that may not be resolved by the proposed robust methods is selection bias \citep{hernan2004}. This could arise from differential selection of individuals in the datasets from which the genetic associations are obtained, or else the selection of genetic variants based on their strength in the dataset under analysis, or if genetic variants were discovered as associated with the exposure in the dataset under analysis. Selection bias may affect all genetic variants in a particular analysis, and so is unlikely to be addressed by the use of the median-based methods proposed in this paper.

We clarify that the objective of this paper is not to provide guidance on how to choose genetic variants for a Mendelian randomization analysis. This is an important question, but not one that it is possible to answer in a general way, in that it requires biological as well as statistical considerations of the specific analysis question in each case. The robust analysis methods presented in this paper are able to weaken the assumptions necessary for the consistent estimation of a causal effect. However, they do not eliminate the need to assess the validity of the instrumental variable assumptions. This is particularly true for the median-based approaches, as the assumption that 50\% of the information in the analysis comes from valid instrumental variables is still required. As a corollary, these robust methods should not be used to promote analysis approaches based on the whole genome for causal inference without further justification.

\begin{center}[Table~\ref{summresults} should appear about here]\end{center}

One assumption that we have made in this paper is that of no causal effect heterogeneity. This means that the same change in the outcome is expected no matter how the exposure is intervened on, and so the same causal effect is identified by different genetic variants that are valid IVs. In practice, it may be that genetic variants influence the exposure via different biological mechanisms. However, empirical evidence for the causal effect of \mbox{LDL-c} on CAD risk provides no evidence of causal effect heterogeneity despite genetic variants having different mechanisms and substantially different magnitudes of association with \mbox{LDL-c} \citep{daveysmith2014}.

Other approaches for the estimation of causal effects with some invalid instrumental variables have been proposed. Kang et al.\ have proposed a method based on penalized regression for detecting and accounting for invalid instruments that provides a consistent estimate of causal effect if at least 50\% of the genetic variants are valid \citep{kang2014}. Han has proposed a similar penalized estimator within the generalized method of moments framework, again with a 50\% breakdown level \citep{han2008}. Koles\'{a}r et al.\ have proposed a method within the framework of k-class estimators with a 100\% breakdown level under the InSIDE assumption \citep{kolesar2014}. The first two methods are similar to the median-based approaches proposed here, and the final method is similar to MR-Egger regression. An alternative approach was proposed by Windmeijer et al.\ based on Hansen’s overidentification test, a test of the homogeneity of the ratio estimates from different candidate instrumental variables \citep{windmeijer2015}. The basic idea of this approach is to report a causal estimate based on genetic variants whose ratio estimates are mutually similar. A related approach based on step-wise selection using a heterogeneity test statistic was proposed by Johnson \citep{johnson2013vig}. While the exclusion of certain variants to consider their potential impact on the overall estimate may be a reasonable sensitivity analysis, a potential danger of such an approach is that of \emph{post hoc} or data-driven analysis, in which genetic variants are cherry-picked for inclusion in the analysis model.

We have chosen in this paper to focus on the two-sample situation using summary data as this is most relevant to Mendelian randomization; all the other methods have been developed for use with individual-level data in a one-sample setting, and hence are not considered in this manuscript. We look forward to further methodological developments in this area. One particular area that may be fruitful for such developments is bias-correction methods in the meta-analysis literature; two particular examples are the trim-and-fill method \citep{duval2000} and the use of pseudo-data \citep{bowden2006}. Equivalently, there may be application of the median-based methods proposed in this paper in the field of meta-analysis, to provide a more robust pooled estimate.

From a practical perspective, it is important to acknowledge the limitations of all methods for obtaining causal inferences. Our aim in presenting the median-based methods in this paper is not to recommend a single authoritative method for all Mendelian randomization analyses. Rather, examining the results from different methods that make different assumptions (IVW, simple median, weighted median, MR-Egger regression) provides a sensitivity analysis that either adds to or questions the robustness of a finding from a Mendelian randomization investigation. If, as in the case of the effect of \mbox{LDL-c} on CAD risk, a causal effect is reported across all methods, then a causal finding is far more plausible than if the methods give contradictory findings. Our advice in Mendelian randomization investigations using multiple genetic variants where the IV assumptions are in doubt for some or all genetic variants, would therefore be to perform and report results from a range of sensitivity analyses using robust methods, including the simple median, weighted median, and MR-Egger regression methods, in addition to the main analysis result.

\vspace{3mm}

\setstretch{1.3}

\subsection*{Acknowledgements}

\noindent \noindent The authors have no conflict of interest. Jack Bowden is supported by a Methodology Research Fellowship from the Medical Research Council (grant number MR/N501906/1). George Davey Smith is supported by the Medical Research Council (grant number MC\_UU\_12013/1-9). Philip C Haycock is supported by a Cancer Research UK Population Research Postdoctoral Fellowship. Stephen Burgess is supported by a fellowship from the Wellcome Trust (100114).

\putbib[masterref]

\end{bibunit}

\clearpage

\section*{Tables}

\begin{table}[hp]

\setlength{\extrarowheight}{4pt}

\begin{small}

\begin{center}

\caption{\label{illustweights} \textbf{Weights and percentiles of weighted median function}}

\begin{tabular}[c]{c|cccccccccc}

\hline

& $\hat{\beta}_1$ & $\hat{\beta}_2$ & $\hat{\beta}_3$ & $\hat{\beta}_4$ & $\hat{\beta}_5$ & $\hat{\beta}_6$ & $\hat{\beta}_7$ & $\hat{\beta}_8$ & $\hat{\beta}_9$ & $\hat{\beta}_{10}$ \\

\hline

Simple median & & & & & & & & & & \\

Weight ($w_j$) & $\frac{1}{10}$ & $\frac{1}{10}$ & $\frac{1}{10}$ & $\frac{1}{10}$ & $\frac{1}{10}$ & $\frac{1}{10}$ & $\frac{1}{10}$ & $\frac{1}{10}$ & $\frac{1}{10}$ & $\frac{1}{10}$ \\

Percentile ($p_j$) & 5 & 15 & 25 & 35 & 45 & 55 & 65 & 75 & 85 & 95 \\

\hline

Weighting 1 & & & & & & & & & & \\

Weight ($w_j$) & $\frac{1}{30}$ & $\frac{2}{30}$ & $\frac{3}{30}$ & $\frac{4}{30}$ & $\frac{5}{30}$ & $\frac{5}{30}$ & $\frac{4}{30}$ & $\frac{3}{30}$ & $\frac{2}{30}$ & $\frac{1}{30}$ \\

Percentile & 1.67 & 6.67 & 15.00 & 26.67 & 41.67 & 58.33 & 73.33 & 85.00 & 93.33 & 98.33 \\

\hline

Weighting 2 & & & & & & & & & & \\

Weight ($w_j$) & $\frac{2}{36}$ & $\frac{3}{36}$ & $\frac{10}{36}$ & $\frac{8}{36}$ & $\frac{5}{36}$ & $\frac{3}{36}$ & $\frac{2}{36}$ & $\frac{1}{36}$ & $\frac{1}{36}$ & $\frac{1}{36}$ \\

Percentile ($p_j$) & 2.78 & 9.72 & 27.78 & 52.78 & 70.83 & 81.94 & 88.89 & 93.06 & 95.83 & 98.61 \\

\hline

\end{tabular}

\caption*{\onehalfspacing Weights and percentiles of the empirical distribution function assigned to the ordered ratio instrumental variable estimates ($\hat{\beta}_j$) for the hypothetical examples given in Figure~\ref{illustdist}.}

\end{center}

\end{small}

\end{table}

%\setlength{\tabcolsep}{5pt}

\begin{sidewaystable}[htbp]

\setlength{\extrarowheight}{2pt}

\begin{minipage}{\textwidth}

%\begin{adjustwidth}{-0.8cm}{-0.8cm}

\begin{center}

\begin{footnotesize}

\centering

\caption{\label{tab:SimScenario1} \textbf{Results from simulation study in two-sample setting with null causal effect}}

\begin{tabular}{cc|cc|cc|cc|cc|cc}

\hline

\blfootnote{Abbreviations: IV, instrumental variable; SE, standard error}

& & & & \multicolumn{2}{c|}{Inverse-variance weighted} & \multicolumn{2}{c|}{Weighted median} & \multicolumn{2}{c|}{Penalized weighted median}

& \multicolumn{2}{c}{MR-Egger regression} \\

\hline

& Proportion of & & & Mean estimate & & Mean estimate & & Mean estimate & & Mean estimate & \\

$N$ & invalid IVs & $F$ & $R^2$ & (mean SE) & Power & (mean SE) & Power & (mean SE) & Power & (mean SE) & Power \\

\hline

\multicolumn{12}{c}{Scenario 1. Balanced pleiotropy, InSIDE assumption satisfied} \\

\hline

10\thinspace000 & 0.1 & 10.7 & 2.6\% & -0.001 (0.114) & 5.4 & -0.001 (0.093) & 3.2 & -0.001 (0.093) & 3.4 & -0.003 (0.287) & 6.3 \\

10\thinspace000 & 0.2 & 10.7 & 2.6\% & 0.001 (0.153) & 6.2 & 0.001 (0.098) & 4.5 & 0.001 (0.098) & 4.0 & -0.001 (0.386) & 6.2 \\

10\thinspace000 & 0.3 & 10.7 & 2.6\% & 0.003 (0.185) & 6.3 & 0.001 (0.103) & 6.2 & 0.001 (0.104) & 5.2 & 0.000 (0.467) & 6.0 \\

20\thinspace000 & 0.1 & 20.5 & 2.5\% & -0.001 (0.107) & 5.1 & 0.000 (0.067) & 3.4 & 0.000 (0.067) & 3.6 & 0.000 (0.305) & 6.0 \\

20\thinspace000 & 0.2 & 20.5 & 2.5\% & 0.002 (0.150) & 5.3 & 0.001 (0.071) & 4.4 & 0.001 (0.071) & 4.4 & -0.004 (0.426) & 6.1 \\

20\thinspace000 & 0.3 & 20.5 & 2.5\% & -0.004 (0.184) & 5.7 & -0.001 (0.075) & 6.4 & -0.001 (0.077) & 6.3 & -0.004 (0.523) & 6.2 \\

\hline

\multicolumn{12}{c}{Scenario 2. Directional pleiotropy, InSIDE assumption satisfied} \\

\hline

10\thinspace000 & 0.1 & 10.7 & 2.6\% & 0.126 (0.111) & 14.6 & 0.033 (0.093) & 4.9 & 0.024 (0.093) & 4.2 & 0.013 (0.279) & 6.3 \\

10\thinspace000 & 0.2 & 10.7 & 2.6\% & 0.256 (0.145) & 37.0 & 0.078 (0.100) & 10.7 & 0.071 (0.102) & 9.6 & 0.037 (0.363) & 6.5 \\

10\thinspace000 & 0.3 & 10.7 & 2.6\% & 0.384 (0.169) & 62.7 & 0.139 (0.109) & 21.8 & 0.149 (0.114) & 22.1 & 0.046 (0.421) & 6.3 \\

20\thinspace000 & 0.1 & 20.5 & 2.5\% & 0.134 (0.104) & 15.0 & 0.026 (0.067) & 4.9 & 0.026 (0.068) & 5.2 & 0.003 (0.295) & 6.1 \\

20\thinspace000 & 0.2 & 20.5 & 2.5\% & 0.271 (0.141) & 42.9 & 0.061 (0.072) & 11.9 & 0.080 (0.078) & 15.8 & 0.011 (0.398) & 6.2 \\

20\thinspace000 & 0.3 & 20.5 & 2.5\% & 0.404 (0.166) & 70.4 & 0.115 (0.080) & 25.4 & 0.177 (0.095) & 35.9 & 0.016 (0.467) & 6.0 \\

\hline

\multicolumn{12}{c}{Scenario 3. Directional pleiotropy, InSIDE assumption not satisfied} \\

\hline

10\thinspace000 & 0.1 & 13.5 & 3.3\% & 0.182 (0.092) & 48.0 & 0.145 (0.095) & 29.9 & 0.062 (0.094) & 12.6 & 0.363 (0.195) & 50.9 \\

10\thinspace000 & 0.2 & 16.3 & 3.9\% & 0.318 (0.105) & 77.2 & 0.303 (0.097) & 61.3 & 0.186 (0.097) & 37.9 & 0.555 (0.204) & 72.5 \\

10\thinspace000 & 0.3 & 19.2 & 4.6\% & 0.421 (0.110) & 91.1 & 0.435 (0.092) & 82.5 & 0.335 (0.095) & 65.9 & 0.651 (0.204) & 83.2 \\

20\thinspace000 & 0.1 & 26.0 & 3.1\% & 0.189 (0.084) & 53.5 & 0.131 (0.072) & 32.4 & 0.059 (0.070) & 13.2 & 0.412 (0.184) & 57.5 \\

20\thinspace000 & 0.2 & 31.7 & 3.8\% & 0.327 (0.100) & 81.0 & 0.290 (0.075) & 63.8 & 0.176 (0.077) & 40.5 & 0.607 (0.198) & 77.1 \\

20\thinspace000 & 0.3 & 37.2 & 4.4\% & 0.427 (0.105) & 93.5 & 0.428 (0.072) & 83.9 & 0.321 (0.077) & 68.4 & 0.697 (0.197) & 86.9 \\

\hline

\end{tabular}

\caption*{\onehalfspacing Mean estimates, mean standard errors, and power of 95\% confidence interval to reject null hypothesis of inverse-variance weighted, weighted median, and MR-Egger regression methods in simulation study for two-sample Mendelian randomization with a null ($\beta=0$) causal effect.}

\end{footnotesize}

\end{center}

%\end{adjustwidth}

\end{minipage}

\end{sidewaystable}

%\setlength{\tabcolsep}{6pt}

\begin{sidewaystable}[htbp]

\setlength{\extrarowheight}{2pt}

\begin{minipage}{\textwidth}

%\begin{adjustwidth}{-0.8cm}{-0.8cm}

\begin{center}

\begin{footnotesize}

\centering

\caption{\label{tab:SimScenario2} \textbf{Results from simulation study in two-sample setting with positive causal effect}}

\begin{tabular}{cc|cc|cc|cc|cc|cc}

\hline

& & & & \multicolumn{2}{c|}{Inverse-variance weighted} & \multicolumn{2}{c|}{Weighted median} & \multicolumn{2}{c|}{Penalized weighted median}

& \multicolumn{2}{c}{MR-Egger regression} \\

\hline

& Proportion of & & & Mean estimate & & Mean estimate & & Mean estimate & & Mean estimate & \\

$N$ & invalid IVs & $F$ & $R^2$ & (mean SE) & Power & (mean SE) & Power & (mean SE) & Power & (mean SE) & Power \\

\hline

\multicolumn{12}{c}{Scenario 1. Balanced pleiotropy, InSIDE assumption satisfied} \\

\hline

10\thinspace000 & 0.1 & 10.7 & 2.6\% & 0.090 (0.116) & 16.2 & 0.085 (0.098) & 12.3 & 0.086 (0.098) & 12.4 & 0.049 (0.292) & 6.7 \\

10\thinspace000 & 0.2 & 10.7 & 2.6\% & 0.092 (0.155) & 11.7 & 0.088 (0.103) & 13.5 & 0.089 (0.103) & 12.7 & 0.052 (0.390) & 6.5 \\

10\thinspace000 & 0.3 & 10.7 & 2.6\% & 0.094 (0.186) & 9.4 & 0.088 (0.109) & 13.4 & 0.089 (0.109) & 12.8 & 0.053 (0.470) & 6.4 \\

20\thinspace000 & 0.1 & 20.5 & 2.5\% & 0.095 (0.108) & 22.1 & 0.092 (0.071) & 24.0 & 0.093 (0.071) & 24.2 & 0.064 (0.309) & 6.8 \\

20\thinspace000 & 0.2 & 20.5 & 2.5\% & 0.097 (0.150) & 13.7 & 0.093 (0.075) & 24.4 & 0.094 (0.075) & 24.1 & 0.060 (0.428) & 6.5 \\

20\thinspace000 & 0.3 & 20.5 & 2.5\% & 0.092 (0.184) & 9.6 & 0.091 (0.079) & 22.6 & 0.092 (0.080) & 22.7 & 0.061 (0.525) & 6.3 \\

\hline

\multicolumn{12}{c}{Scenario 2. Directional pleiotropy, InSIDE assumption satisfied} \\

\hline

10\thinspace000 & 0.1 & 10.7 & 2.6\% & 0.217 (0.114) & 45.9 & 0.121 (0.099) & 20.9 & 0.111 (0.099) & 18.3 & 0.066 (0.285) & 7.4 \\

10\thinspace000 & 0.2 & 10.7 & 2.6\% & 0.348 (0.148) & 68.0 & 0.168 (0.107) & 32.5 & 0.160 (0.108) & 28.7 & 0.090 (0.367) & 7.3 \\

10\thinspace000 & 0.3 & 10.7 & 2.6\% & 0.475 (0.171) & 84.3 & 0.232 (0.116) & 47.6 & 0.239 (0.121) & 46.1 & 0.099 (0.425) & 7.0 \\

20\thinspace000 & 0.1 & 20.5 & 2.5\% & 0.230 (0.105) & 61.4 & 0.120 (0.071) & 37.2 & 0.119 (0.072) & 36.0 & 0.067 (0.298) & 7.1 \\

20\thinspace000 & 0.2 & 20.5 & 2.5\% & 0.366 (0.143) & 80.3 & 0.157 (0.077) & 52.5 & 0.173 (0.082) & 53.9 & 0.076 (0.401) & 6.7 \\

20\thinspace000 & 0.3 & 20.5 & 2.5\% & 0.500 (0.168) & 92.3 & 0.213 (0.086) & 66.3 & 0.269 (0.099) & 72.0 & 0.081 (0.469) & 6.4 \\

\hline

\multicolumn{12}{c}{Scenario 3. Directional pleiotropy, InSIDE assumption not satisfied} \\

\hline

10\thinspace000 & 0.1 & 13.5 & 3.3\% & 0.274 (0.095) & 71.1 & 0.238 (0.101) & 48.5 & 0.154 (0.099) & 29.1 & 0.432 (0.202) & 55.5 \\

10\thinspace000 & 0.2 & 16.3 & 3.9\% & 0.411 (0.107) & 89.9 & 0.400 (0.103) & 75.8 & 0.283 (0.103) & 55.6 & 0.634 (0.209) & 76.5 \\

10\thinspace000 & 0.3 & 19.2 & 4.6\% & 0.515 (0.112) & 96.8 & 0.533 (0.099) & 90.5 & 0.433 (0.101) & 78.5 & 0.736 (0.209) & 86.9 \\

20\thinspace000 & 0.1 & 26.0 & 3.1\% & 0.285 (0.085) & 81.1 & 0.229 (0.076) & 63.8 & 0.153 (0.074) & 47.6 & 0.491 (0.189) & 62.1 \\

20\thinspace000 & 0.2 & 31.7 & 3.8\% & 0.423 (0.101) & 93.4 & 0.391 (0.079) & 85.0 & 0.274 (0.081) & 71.1 & 0.694 (0.201) & 81.2 \\

20\thinspace000 & 0.3 & 37.2 & 4.4\% & 0.525 (0.106) & 98.0 & 0.529 (0.076) & 94.7 & 0.420 (0.082) & 87.1 & 0.788 (0.200) & 90.2 \\

\hline

\end{tabular}

\caption*{\onehalfspacing Mean estimates, mean standard errors, and power of 95\% confidence interval to reject null hypothesis of inverse-variance weighted, weighted median, and MR-Egger regression methods in simulation study for two-sample Mendelian randomization with a positive ($\beta=0.1$) causal effect.}

\end{footnotesize}

\end{center}

%\end{adjustwidth}

\end{minipage}

\end{sidewaystable}

\begin{table}[htbp]

\setlength{\extrarowheight}{4pt}

\centering

\begin{small}

\begin{center}

\begin{minipage}{\textwidth}

\centering

\caption{\label{exampletab} \textbf{Results from applied example}}

\begin{tabular}[c]{c|cc|cc}

\hline

& & & \multicolumn{2}{c}{Primary association } \\

& \multicolumn{2}{c|}{All genetic variants} & \multicolumn{2}{c}{with target exposure} \\

Analysis method & Estimate (SE) & p-value \footnote{p-values are indicated as: * = $p<0.05$, ** = $p<0.01$, *** = $p<0.0001$}

& Estimate (SE) & p-value \footnotemark[1] \\

\hline

\multicolumn{5}{c}{Low-density lipoprotein cholesterol (\mbox{LDL-c})} \\

\hline

Inverse-variance weighted & 0.482 (0.060) & *** & 0.470 (0.055) & *** \\

Simple median & 0.429 (0.070) & *** & 0.429 (0.079) & *** \\

Weighted median & 0.458 (0.065) & *** & 0.457 (0.065) & *** \\

Penalized weighted median & 0.457 (0.063) & *** & 0.457 (0.067) & *** \\

MR-Egger regression: slope & 0.617 (0.103) & *** & 0.562 (0.094) & *** \\

\hphantom{MR-Egger regress:} intercept & -0.009 (0.005) & & -0.006 (0.005) & \\

\hline

\multicolumn{5}{c}{High-density lipoprotein cholesterol (\mbox{HDL-c})} \\

\hline

Inverse-variance weighted & -0.254 (0.070) & *** & -0.137 (0.066) & * \\

Simple median & -0.267 (0.090) & ** & -0.224 (0.085) & ** \\

Weighted median & -0.069 (0.071) & & -0.066 (0.065) & \\

Penalized weighted median & -0.071 (0.068) & & -0.064 (0.066) & \\

MR-Egger regression: slope & -0.013 (0.115) & & 0.092 (0.107) & \\

\hphantom{MR-Egger regress:} intercept & -0.014 (0.005) & * & -0.013 (0.005) & * \\

\hline

\multicolumn{5}{c}{Triglycerides} \\

\hline

Inverse-variance weighted & 0.416 (0.081) & *** & 0.417 (0.095) & ** \\

Simple median & 0.512 (0.101) & *** & 0.565 (0.105) & *** \\

Weighted median & 0.516 (0.084) & *** & 0.521 (0.087) & *** \\

Penalized weighted median & 0.528 (0.078) & *** & 0.539 (0.089) & *** \\

MR-Egger regression: slope & 0.422 (0.140) & ** & 0.464 (0.155) & ** \\

\hphantom{MR-Egger regress:} intercept & -0.000 (0.008) & & -0.004 (0.009) & \\

\hline

\end{tabular}

\end{minipage}

\end{center}

\end{small}

\caption*{\onehalfspacing Estimates (standard errors) of causal effects of lipid fractions on coronary artery disease risk. Estimates are log odds ratios per 1 standard deviation increase in the exposure. The intercept term in MR-Egger regression provides a test of directional pleiotropy.}

\end{table}

\begin{table}[htbp]

\setlength{\extrarowheight}{4pt}

\centering

\begin{small}

\begin{center}

\begin{minipage}{\textwidth}

\centering

\begin{adjustwidth}{-0.4cm}{-0.4cm}

\caption{\label{summresults} \textbf{Summary of methods considered in this paper}}

\begin{tabular}[c]{p{0.18\textwidth}cccp{0.5\textwidth}}

\hline

Method & Breakdown & IV2 & IV3 & Comments \\

\hline

Two-stage least squares & 0\% & \cross & \cross & Requires individual-level data.

Biased when at least 1 genetic variant is an invalid IV. \\ [2mm]

Inverse-variance weighted (IVW) & 0\% & \cross & \cross & Equivalent to two-stage least squares method with summary data.

Also biased when at least 1 genetic variant is an invalid IV. \\ [2mm]

Simple median & 50\% & \tick & \tick & Consistent when 50\% of genetic variants are valid IVs.

Inefficient compared with IVW and weighted median methods. \\ [2mm]

Weighted \mbox{median} & 50\% & \tick & \tick & Consistent when 50\% of weight contributed by genetic variants is valid.

Efficiency is similar to that of IVW method. \\ [2mm]

Penalized weighted \mbox{median} & 50\% & \tick & \tick & Equivalent to weighted median when there is no causal effect heterogeneity.

Downweights the contribution of heterogeneous variants, so may have better finite sample properties, particularly if there is directional pleiotropy. \\ [2mm]

MR-Egger regression & 100\% & \cross & \tick & Consistent when 100\% of genetic variants are invalid, but requires variants to satisfy a weaker assumption (the InSIDE assumption).

This assumption is not automatically violated by an association between a genetic variant and a confounder, but it would be violated if several variants were associated with the same confounder.

Substantially less efficient than IVW and median-based methods, and more susceptible to weak instrument bias in a one-sample setting. \\

\hline

\end{tabular}

\end{adjustwidth}

\end{minipage}

\end{center}

\end{small}

\caption*{\onehalfspacing Breakdown refers to the breakdown level, the proportion of information that can come from invalid instrumental variables (IVs) before the method gives biased estimates. IV2 and IV3 refer to whether violations of the second (no association with confounders) and third (no direct effect on the outcome) instrumental variable assumptions are allowed (\tick) or not allowed (\cross).}

\end{table}

\clearpage

\section*{Figure legends}

\indent \indent \textbf{Figure \ref{dag}:} Illustrative diagram representing the hypothesized relationships between genetic variant $G_j$, exposure $X$, disease $Y$ and confounders $U$ when $G_j$ is a valid instrumental variable (IV). Red crosses indicate violations of assumptions IV2 and IV3 that potentially lead to invalid inferences from conventional methods.

\textbf{Figure \ref{illustrate}:} Fictional example of a Mendelian randomization analysis with 10 genetic variants -- 6 valid instrumental variables (hollow circles) and 4 invalid instrumental variables (solid circles) for finite sample size (left) and infinite sample size (right) showing inverse variance weighted (IVW, solid line) and simple median (dashed line) estimates compared with the true causal effect (dotted line).

\textbf{Figure \ref{illustdist}:} Empirical distribution functions of ordered ratio instrumental variable estimates ($\hat{\beta}_j$) used for calculation of the simple median estimate (black) and two weighted median estimates (shown in red and blue) using the weights given in Table~\ref{illustweights}.

\textbf{Figure \ref{scatters}:} Scatter plots of genetic associations with the outcome (coronary artery disease risk, CAD) against genetic associations with the exposure (low-density lipoprotein cholesterol, \mbox{LDL-c}; high-density lipoprotein cholesterol, \mbox{HDL-c}; triglycerides). Left side: all genetic variants, right side: genetic variants having primary association with the target exposure.

\clearpage

\begin{bibunit}[apalike]

\renewcommand{\thesection}{A\arabic{section}}

\renewcommand{\thesubsection}{A.\arabic{subsection}}

\renewcommand{\thetable}{A\arabic{table}}

\renewcommand{\thefigure}{A\arabic{figure}}

\renewcommand{\thepage}{A\arabic{page}}

\setcounter{table}{0}

\setcounter{figure}{0}

\setcounter{page}{1}

\renewcommand{\tablename}{Web Table}

\renewcommand{\figurename}{Web Figure}

\setcounter{section}{0}

\setcounter{subsection}{0}

\section*{Web Appendix 1: Some technical remarks}

The concept of a weighted median is one that goes back to the 19th century \citep{edgeworth1887, edgeworth1888}. However, there is no universally agreed method for calculating a weighted median (or an arbitrary percentile from a finite sample; see \url{https://en.wikipedia.org/w/index.php?title=Percentile\&oldid=668900109} for a non-technical overview). We have settled with the weighted percentile definition in this paper after examining the performance of a number of different methods. An alternative approach would be to define a weighted empirical distribution and take the median from this distribution. The cumulative distribution function $F(p), 0 \leq p \leq 1$ for the empirical distribution would be defined as:

\begin{equation}

F(p) = \hat{\beta}_{j}, \mbox{ for $j$ such that } s_{j-1} < p < s_{j}

\end{equation}

where $s_j = \sum_{k=1}^j w_k$ is the cumulative sum of weights up to the $j$th genetic variant. The weighted median estimate according to the empirical distribution method would be $F(0.5)$. Code for the empirical distribution implementation of a weighted median method (as well as code for the weighted percentile method used in this paper) is given in Web Appendix 2. For the simple median estimator, this difference is moot.

The advantage of the empirical distribution method is that it is always consistent if over 50\% of the weight in the analysis comes from valid IVs. In the weighted percentile method advocated in this paper, there are corner cases that can be constructed in which this is not true. For instance, if $w_1 = 0.3$, $w_2 = 0.15$, $w_3 = 0.08$, and $w_4 = 0.47$, and the only first three variants are valid IVs, then the weighted percentile method will not be consistent, as it will extrapolate between the third and fourth ratio estimates. However, provided that there are a moderately large number of variants (as will normally be the case in applied practice) and provided that a large proportion of the weight is not concentrated in one single variant, this is unlikely to be a serious issue. If the greatest weight is 10\%, consistency in the weighted percentile method is guaranteed provided that 55\% of the weight comes from valid IVs. There are several advantages of using the weighted percentile approach, which in our opinion outweigh this technical deficiency. By extrapolating, the weighted median estimate is not constrained to take the value of one of the ratio estimates. This gives greater stability to the point estimate. Bootstrap confidence intervals are also more reliable, as the distribution of estimates from each iteration of the bootstrap will be more continuous.

In any event, the aim of this paper is not to advocate a single weighted median method as superior to other weighted median methods (in the same way as we would not argue for reliance on any single sensitivity analysis for a Mendelian randomization investigation). We look forward to further technical developments in finding an optimal weighted median method.

\clearpage

\section*{Web Appendix 2: Software code}

We provide R code for implementing the weighted median approach using the weighted percentile function (as performed in the simulations and example of the paper).

\scriptsize{

\begin{verbatim}

weighted.median <- function(betaIV.in, weights.in) {

betaIV.order = betaIV.in[order(betaIV.in)]

weights.order = weights.in[order(betaIV.in)]

weights.sum = cumsum(weights.order)-0.5*weights.order

weights.sum = weights.sum/sum(weights.order)

below = max(which(weights.sum<0.5))

weighted.est = betaIV.order[below] + (betaIV.order[below+1]-betaIV.order[below])*

(0.5-weights.sum[below])/(weights.sum[below+1]-weights.sum[below])

return(weighted.est) }

weighted.median.boot = function(betaXG.in, betaYG.in, sebetaXG.in, sebetaYG.in, weights.in){

med = NULL

for(i in 1:1000){

betaXG.boot = rnorm(length(betaXG.in), mean=betaXG.in, sd=sebetaXG.in)

betaYG.boot = rnorm(length(betaYG.in), mean=betaYG.in, sd=sebetaYG.in)

betaIV.boot = betaYG.boot/betaXG.boot

med[i] = weighted.median(betaIV.boot, weights.in)

}

return(sd(med)) }

betaIV = betaYG/betaXG # ratio estimates

weights = (sebetaYG/betaXG)^-2 # inverse-variance weights

betaIVW = sum(betaYG*betaXG*sebetaYG^-2)/sum(betaXG^2*sebetaYG^-2)

# IVW estimate

penalty = pchisq(weights*(betaIV-betaIVW)^2, df=1, lower.tail=FALSE)

pen.weights = weights*pmin(1, penalty*20) # penalized weights

betaWM = weighted.median(betaIV, weights) # weighted median estimate

sebetaWM = weighted.median.boot(betaXG, betaYG, sebetaXG, sebetaYG, weights)

# standard error

betaPWM = weighted.median(betaIV, pen.weights) # penalized weighted median estimate

sebetaPWM = weighted.median.boot(betaXG, betaYG, sebetaXG, sebetaYG, pen.weights)

# standard error

\end{verbatim}

}

\normalsize{

We found that the bootstrap confidence interval (that is, the 2.5th to the 97.5th percentile of the bootstrap estimates) gives poor coverage, tending to be too conservative. However, the bootstrap standard error (the standard deviation of the bootstrap estimates) gave more reasonable coverage using a normal approximation (estimate $\pm \, 1.96 \, \times$ standard error) to form a 95\% confidence interval.

An alternative interpretation of a weighted median estimator based on an empirical distribution function is discussed in Web Appendix 1.

}

\scriptsize{

\begin{verbatim}

weighted.median.empirical <- function(betaIV.in, weights.in) {

betaIV.order = betaIV.in[order(betaIV.in)]

weights.order = weights.in[order(betaIV.in)]

weights.sum = cumsum(weights.order)

weights.sum = weights.sum/sum(weights.order)

which.below = max(which(weights.sum<0.5))

return(betaIV.order[which.below+1]) }

betaWME = weighted.median.empirical(betaIV, weights)

# alternative weighted median estimate

\end{verbatim}

}

\normalsize{

An inverse-standard error weighted can be implemented by replacing the inverse-variance weights with:

}

\scriptsize{

\begin{verbatim}

weights = (sebetaYG/betaXG)^-1 # inverse-standard error weights

\end{verbatim}

}

\normalsize{

The IVW and MR-Egger regression approaches used in this paper can be obtained using the following code:

}

\scriptsize{

\begin{verbatim}

betaIVW = summary(lm(betaYG~betaXG-1, weights=sebetaYG^-2))$coef[1,1]

sebetaIVW = summary(lm(betaYG~betaXG-1, weights=sebetaYG^-2))$coef[1,2]/

min(summary(lm(betaYG~betaXG-1, weights=sebetaYG^-2))$sigma, 1)

betaEGGER = summary(lm(betaYG~betaXG, weights=sebetaYG^-2))$coef[2,1]

sebetaEGGER = summary(lm(betaYG~betaXG, weights=sebetaYG^-2))$coef[2,2]/

min(summary(lm(betaYG~betaXG, weights=sebetaYG^-2))$sigma, 1)

\end{verbatim}

}

\normalsize{

This differs slightly from the code originally given in Bowden et al.\ \citep{bowden2015} (although it is similar to that used in the simulation study of Bowden et al.), as it allows for heterogeneity in the causal effects from different IVs via not constraining the residual standard error to be 1. If the estimate of the residual standard error is less than 1, then we divide the standard errors of the coefficients by this estimate to ensure that they are not overly precise. However, if it is greater than 1, then we do not divide by the residual standard error, as this results in over-precision when there is true heterogeneity between the causal effects identified by different IVs. In particular, dividing by the residual standard error in the simulation study resulted in substantial under-coverage (as there was substantial heterogeneity in the causal estimates from each genetic variant due to the invalid IVs). However, if there is substantial heterogeneity in practice, this would call into question the validity of the IVW method, and a stricter filtering as to which of the genetic variants to include in the analysis should be applied.

Power for each method was based on a 95\% confidence interval (estimate $\pm \, 1.96 \, \times$ standard error). This is slightly too narrow in the case of the IVW and MR-Egger methods in the presence of heterogeneity, as a t-distribution should be used in this case. With 25 genetic variants, this would lead to a confidence interval of estimate $\pm \, 2.06 \, \times$ standard error for the IVW method (t-distribution on 24 degrees of freedom), and estimate $\pm \, 2.07 \, \times$ standard error for the MR-Egger method (23 degrees of freedom). We used a normal approximation for consistency between the methods. Consequently, Type 1 error rates in the two-sample setting (Table~\ref{tab:SimScenario1}) were slightly above the nominal 5\% level, but not greatly so.

}

\clearpage

\section*{Web Appendix 3: Simulation setup and additional results}

\normalsize{The data-generating model was as follows:}

\begin{align}

U_{i} &= \sum^{J}_{j=1} \phi_{j} G_{ij} + \epsilon^{U}_{i} \nonumber \\

X_{i} &= \sum^{J}_{j=1} \gamma_{j} G_{ij} + U_{i} + \epsilon^{X}_{i} \nonumber \\

Y_{i} &= \sum^{J}_{j=1} \alpha_{j} G_{ij} + \beta X_{i} + U_{i} + \epsilon^{Y}_{i}. \nonumber

\end{align}

for participants indexed by $i = 1, \ldots, N$, and genetic variants indexed by $j = 1, \ldots, J$. The error terms $\epsilon^{U}_{i}$, $\epsilon^{X}_{i}$, and $\epsilon^{Y}_{i}$ were each drawn independently from standard normal distributions. The genetic effects on the exposure $\gamma_j$ are drawn from a uniform distribution between 0.03 and 0.1. Pleiotropic effects $\alpha_j$ and $\phi_j$ were set to zero if the genetic variant was a valid instrumental variable. Otherwise (with probability 0.1, 0.2, or 0.3):

\begin{itemize}

\item[1.] In Scenario 1 (balanced pleiotropy, InSIDE satisfied), the $\alpha_j$ parameter was drawn from a uniform distribution between $-0.2$ and 0.2.

\item[2.] In Scenario 2 (directional pleiotropy, InSIDE satisfied), the $\alpha_j$ parameter was drawn from a uniform distribution between 0 and 0.2.

\item[3.] In Scenario 3 (directional pleiotropy, InSIDE not satisfied), the $\phi_j$ parameter was drawn from a uniform distribution between $-0.2$ and 0.2.

\end{itemize}

The causal effect of the exposure on the outcome was either $\beta_X = 0$ (null causal effect) or $\beta_X = 0.1$ (positive causal effect). A total of 10\thinspace000 simulated datasets were generated for sample sizes of $N = 10\thinspace000$ and $20\thinspace000$ participants. Only the summary data, that is genetic associations with the exposure and with the outcome and their standard errors as estimated by univariate regression on the genetic variants in turn, were used by the analysis methods. In the two-sample setting, data were generated on $2N$ participants, and genetic associations with the exposure were estimated in the first $N$ participants, and genetic associations with the outcome in the second $N$ participants. The Monte Carlo standard error for the mean estimates was 0.002 or less, and for the power was less than 0.5\% in all cases.

The standard errors used in the IVW and MR-Egger methods in this simulation correspond to those used in the simulations of Bowden et al.\ \citep{bowden2015}, but differ from those previously recommended for use in the IVW \citep{johnson2013vig, burgess2013genepi} and originally recommended for the MR-Egger regression method (see Web Appendix 2 above). The reason is that the IVW method corresponds to a fixed-effect meta-analysis of the ratio estimates from each genetic variant. In the examples of this paper, there is heterogeneity in the ratio estimates, and so a fixed-effect analysis is inappropriate, and leads to overly precise confidence intervals and inflated Type 1 error rates. A conventional random-effects meta-analysis would not be wise, as the random-effects estimate upweights outlying estimates, inflating the influence of pleiotropic genetic variants on the analysis. Hence we have reached a compromise, similar to that suggested by Copas et al.\ \citep{henmi2010}, that we take the point estimate from a fixed-effect analysis, but allow confidence intervals to be inflated by heterogeneity as per a random-effects analysis. This is achieved by performing a weighted regression as described in the description of MR-Egger regression, but not setting the residual standard error in the regression to be 1, as is recommended in meta-analysis to correspond to a fixed-effect analysis (unless the estimate of the residual standard error is less than 1, in which case we divide the standard errors of the coefficients by the estimate of the residual standard error to avoid over-precision). This is equivalent to a multiplicative random-effects model \citep{thompson1999}.

Results in a two-sample setting are presented in the main manuscript. In a one-sample setting, estimates from the two-stage least squares method (individual-level data) and the IVW method (summary data) are known to be biased in the direction of the observational association; this is known as weak instrument bias. In a two-sample setting, estimates are less biased, and any bias is in the direction of the null (conservative bias) \citep{pierce2013}. Scenario 1 of Table~\ref{tab:SimScenario2} suggests that the same is true for the median-based and MR-Egger methods, with some bias in the direction of the null in a two-sample setting with balanced pleiotropy. When pleiotropy is unbalanced, bias due to pleiotropy seems to be stronger than any attenuation due to weak instruments, at least for the parameters considered in this paper.

Web Tables~\ref{tab:SimScenario1a} (null causal effect) and \ref{tab:SimScenario2a} (positive causal effect) present results in a one-sample setting. In a one-sample setting, all of the methods are affected by weak instrument bias. Estimates under the null are biased in the direction of the observational confounded association, and Type 1 error rates are inflated. Estimates from MR-Egger regression are particularly affected, with substantially more bias than estimates from the other methods. The weighted median methods have better Type 1 error properties under the null than those of the IVW method, although not as good as the MR-Egger method in Scenario 2 (but better in Scenarios 1 and 3). Bias in all methods reduces as the sample size increases.

Web Table~\ref{tab:SimScenario1c} presents results in a two-sample setting for the simple median method, and for an inverse-standard error weighted median method, for which the unstandardized weights are:

\begin{equation}

w'_j = \frac{\hat{\gamma}_j}{\sigma_{Yj}} \notag

\end{equation}

Results from the (inverse-variance) weighted median method considered above are reproduced from Tables~\ref{tab:SimScenario1} and \ref{tab:SimScenario2} for comparison. Estimates from the simple median method are less precise than those from the weighted median methods, but the differences are not substantial in this simulation setting. Bias and Type 1 error in Scenarios 1 and 2 are similar to those from the (inverse-variance) weighted median method, but in Scenario 3 the bias and Type 1 error properties are much improved. However, this is due to an artefact of the simulation, as invalid genetic variants are stronger on average than valid instruments, as they are additionally associated with the exposure through the confounder. Hence, it is not clear that the simple median method should be preferred to a weighted median method on the basis of these simulations, but it does provide another valuable sensitivity analysis that gives consistent estimates under a slightly different assumption (simple median method assumes that 50\% of variants are valid instrumental variables, weighted median methods assume that 50\% of the weight comes from valid instrumental variables).

Estimates from the inverse-standard error weighted median method are slightly more precise than those from the inverse-variance weighted median method. Type 1 error rates of the inverse-standard error method are slightly worse in Scenario 2, but better in Scenario 3. Power to detect a causal effect is better in Scenario 1. However, we do not expect these results to be fully generalizable, and hence would not advocate one implementation of the method over others. In fact, both the simple median and inverse-standard error method reported a causal effect of \mbox{HDL-c} on CAD risk in the applied example, suggesting that the inverse-variance weighted median method would be preferred in this single case.

%\setlength{\tabcolsep}{5pt}

\begin{sidewaystable}[htbp]

\setlength{\extrarowheight}{2pt}

\begin{minipage}{\textwidth}

%\begin{adjustwidth}{-0.8cm}{-0.8cm}

\begin{center}

\begin{footnotesize}

\centering

\caption{\label{tab:SimScenario1a} \textbf{Results from simulation study in one-sample setting with null causal effect}}

\begin{tabular}{cc|cc|cc|cc|cc|cc}

\hline

\footnotetext{Abbreviation: SE, standard error}

& & & & \multicolumn{2}{c|}{Inverse-variance weighted} & \multicolumn{2}{c|}{Weighted median} & \multicolumn{2}{c|}{Penalized weighted median}

& \multicolumn{2}{c}{MR-Egger regression} \\

\hline

& Proportion of & & & Mean estimate & & Mean estimate & & Mean estimate & & Mean estimate & \\

$N$ & invalid IVs & $F$ & $R^2$ & (mean SE) & Power & (mean SE) & Power & (mean SE) & Power & (mean SE) & Power \\

\hline

\multicolumn{12}{c}{Scenario 1. Balanced pleiotropy, InSIDE assumption satisfied} \\

\hline

10\thinspace000 & 0.1 & 10.7 & 2.6\% & 0.045 (0.114) & 8.7 & 0.066 (0.092) & 9.0 & 0.063 (0.093) & 8.4 & 0.233 (0.284) & 18.6 \\

10\thinspace000 & 0.2 & 10.7 & 2.6\% & 0.045 (0.153) & 7.6 & 0.066 (0.097) & 9.9 & 0.061 (0.097) & 8.7 & 0.229 (0.383) & 12.4 \\

10\thinspace000 & 0.3 & 10.7 & 2.6\% & 0.042 (0.184) & 7.1 & 0.063 (0.103) & 10.3 & 0.057 (0.103) & 8.6 & 0.224 (0.463) & 9.9 \\

20\thinspace000 & 0.1 & 20.5 & 2.5\% & 0.022 (0.106) & 6.3 & 0.036 (0.067) & 6.5 & 0.033 (0.067) & 6.0 & 0.165 (0.300) & 13.7 \\

20\thinspace000 & 0.2 & 20.5 & 2.5\% & 0.024 (0.150) & 6.3 & 0.037 (0.070) & 8.4 & 0.033 (0.071) & 7.8 & 0.180 (0.425) & 9.7 \\

20\thinspace000 & 0.3 & 20.5 & 2.5\% & 0.020 (0.183) & 6.4 & 0.034 (0.075) & 9.2 & 0.030 (0.076) & 8.6 & 0.165 (0.522) & 7.7 \\

\hline

\multicolumn{12}{c}{Scenario 2. Directional pleiotropy, InSIDE assumption satisfied} \\

\hline

10\thinspace000 & 0.1 & 10.7 & 2.6\% & 0.173 (0.110) & 30.4 & 0.099 (0.093) & 15.7 & 0.085 (0.093) & 12.4 & 0.248 (0.275) & 19.6 \\

10\thinspace000 & 0.2 & 10.7 & 2.6\% & 0.300 (0.142) & 55.4 & 0.139 (0.100) & 25.7 & 0.121 (0.100) & 20.1 & 0.257 (0.358) & 14.2 \\

10\thinspace000 & 0.3 & 10.7 & 2.6\% & 0.430 (0.165) & 78.4 & 0.199 (0.109) & 40.1 & 0.190 (0.112) & 35.0 & 0.280 (0.417) & 12.1 \\

20\thinspace000 & 0.1 & 20.5 & 2.5\% & 0.160 (0.104) & 24.3 & 0.063 (0.067) & 12.7 & 0.058 (0.068) & 11.5 & 0.177 (0.295) & 13.8 \\

20\thinspace000 & 0.2 & 20.5 & 2.5\% & 0.295 (0.140) & 55.3 & 0.098 (0.072) & 24.2 & 0.106 (0.077) & 24.6 & 0.189 (0.396) & 10.0 \\

20\thinspace000 & 0.3 & 20.5 & 2.5\% & 0.431 (0.165) & 79.9 & 0.146 (0.080) & 39.2 & 0.197 (0.095) & 44.9 & 0.194 (0.465) & 8.8 \\

\hline

\multicolumn{12}{c}{Scenario 3. Directional pleiotropy, InSIDE assumption not satisfied} \\

\hline

10\thinspace000 & 0.1 & 13.6 & 3.3\% & 0.222 (0.089) & 61.3 & 0.202 (0.094) & 45.1 & 0.118 (0.092) & 23.1 & 0.516 (0.180) & 69.5 \\

10\thinspace000 & 0.2 & 16.3 & 3.9\% & 0.347 (0.101) & 84.0 & 0.339 (0.094) & 72.7 & 0.226 (0.094) & 48.8 & 0.650 (0.190) & 82.3 \\

10\thinspace000 & 0.3 & 19.2 & 4.6\% & 0.445 (0.106) & 94.9 & 0.458 (0.090) & 89.0 & 0.360 (0.092) & 73.9 & 0.723 (0.191) & 90.0 \\

20\thinspace000 & 0.1 & 26.0 & 3.1\% & 0.206 (0.082) & 60.2 & 0.157 (0.071) & 42.6 & 0.084 (0.069) & 20.6 & 0.509 (0.173) & 69.2 \\

20\thinspace000 & 0.2 & 31.7 & 3.8\% & 0.343 (0.097) & 85.2 & 0.311 (0.073) & 72.6 & 0.193 (0.075) & 48.1 & 0.673 (0.189) & 83.4 \\

20\thinspace000 & 0.3 & 37.1 & 4.4\% & 0.442 (0.103) & 95.3 & 0.441 (0.071) & 89.0 & 0.332 (0.076) & 74.3 & 0.744 (0.191) & 90.3 \\

\hline

\end{tabular}

\caption*{\onehalfspacing Mean estimates, mean standard errors, and power of 95\% confidence interval to reject null hypothesis of inverse-variance weighted, weighted median, and MR-Egger regression methods in simulation study for one-sample Mendelian randomization with a null ($\beta=0$) causal effect.}

\end{footnotesize}

\end{center}

%\end{adjustwidth}

\end{minipage}

\end{sidewaystable}

%\setlength{\tabcolsep}{6pt}

\begin{sidewaystable}[htbp]

\setlength{\extrarowheight}{2pt}

\begin{minipage}{\textwidth}

%\begin{adjustwidth}{-0.8cm}{-0.8cm}

\begin{center}

\begin{footnotesize}

\centering

\caption{\label{tab:SimScenario2a} \textbf{Results from simulation study in one-sample setting with positive causal effect}}

\begin{tabular}{cc|cc|cc|cc|cc|cc}

\hline

& & & & \multicolumn{2}{c|}{Inverse-variance weighted} & \multicolumn{2}{c|}{Weighted median} & \multicolumn{2}{c|}{Penalized weighted median}

& \multicolumn{2}{c}{MR-Egger regression} \\

\hline

& Proportion of & & & Mean estimate & & Mean estimate & & Mean estimate & & Mean estimate & \\

$N$ & invalid IVs & $F$ & $R^2$ & (mean SE) & Power & (mean SE) & Power & (mean SE) & Power & (mean SE) & Power \\

\hline

\multicolumn{12}{c}{Scenario 1. Balanced pleiotropy, InSIDE assumption satisfied} \\

\hline

10\thinspace000 & 0.1 & 10.7 & 2.6\% & 0.145 (0.114) & 33.4 & 0.166 (0.097) & 40.9 & 0.164 (0.097) & 39.6 & 0.333 (0.284) & 29.6 \\

10\thinspace000 & 0.2 & 10.7 & 2.6\% & 0.146 (0.153) & 20.8 & 0.166 (0.102) & 37.7 & 0.161 (0.102) & 36.0 & 0.329 (0.383) & 18.1 \\

10\thinspace000 & 0.3 & 10.7 & 2.6\% & 0.143 (0.184) & 14.6 & 0.163 (0.108) & 35.1 & 0.158 (0.108) & 32.7 & 0.324 (0.463) & 13.7 \\

20\thinspace000 & 0.1 & 20.5 & 2.5\% & 0.122 (0.106) & 33.4 & 0.136 (0.070) & 50.9 & 0.133 (0.070) & 49.3 & 0.265 (0.300) & 23.8 \\

20\thinspace000 & 0.2 & 20.5 & 2.5\% & 0.124 (0.150) & 18.5 & 0.137 (0.074) & 47.7 & 0.133 (0.074) & 46.2 & 0.280 (0.425) & 13.9 \\

20\thinspace000 & 0.3 & 20.5 & 2.5\% & 0.120 (0.183) & 13.1 & 0.134 (0.078) & 43.2 & 0.130 (0.080) & 40.9 & 0.266 (0.521) & 10.2 \\

\hline

\multicolumn{12}{c}{Scenario 2. Directional pleiotropy, InSIDE assumption satisfied} \\

\hline

10\thinspace000 & 0.1 & 10.7 & 2.6\% & 0.273 (0.110) & 75.5 & 0.199 (0.098) & 54.4 & 0.183 (0.098) & 47.4 & 0.348 (0.275) & 31.5 \\

10\thinspace000 & 0.2 & 10.7 & 2.6\% & 0.400 (0.142) & 87.2 & 0.240 (0.106) & 64.0 & 0.217 (0.105) & 54.7 & 0.357 (0.358) & 21.2 \\

10\thinspace000 & 0.3 & 10.7 & 2.6\% & 0.530 (0.165) & 95.6 & 0.299 (0.116) & 74.8 & 0.282 (0.117) & 67.7 & 0.380 (0.417) & 17.5 \\

20\thinspace000 & 0.1 & 20.5 & 2.5\% & 0.260 (0.104) & 79.5 & 0.163 (0.071) & 66.0 & 0.156 (0.071) & 60.6 & 0.277 (0.295) & 24.1 \\

20\thinspace000 & 0.2 & 20.5 & 2.5\% & 0.395 (0.140) & 90.7 & 0.198 (0.076) & 77.0 & 0.200 (0.080) & 73.0 & 0.289 (0.396) & 14.8 \\

20\thinspace000 & 0.3 & 20.5 & 2.5\% & 0.531 (0.164) & 96.9 & 0.246 (0.085) & 84.3 & 0.286 (0.098) & 83.0 & 0.294 (0.465) & 11.9 \\

\hline

\multicolumn{12}{c}{Scenario 3. Directional pleiotropy, InSIDE assumption not satisfied} \\

\hline

10\thinspace000 & 0.1 & 13.6 & 3.3\% & 0.322 (0.089) & 87.9 & 0.302 (0.100) & 74.0 & 0.218 (0.097) & 56.5 & 0.617 (0.180) & 78.9 \\

10\thinspace000 & 0.2 & 16.3 & 3.9\% & 0.447 (0.101) & 95.6 & 0.439 (0.100) & 89.6 & 0.326 (0.099) & 75.1 & 0.751 (0.190) & 87.8 \\

10\thinspace000 & 0.3 & 19.2 & 4.6\% & 0.545 (0.106) & 98.8 & 0.558 (0.096) & 96.0 & 0.461 (0.097) & 88.3 & 0.823 (0.191) & 93.4 \\

20\thinspace000 & 0.1 & 26.0 & 3.1\% & 0.306 (0.082) & 89.9 & 0.258 (0.075) & 81.8 & 0.181 (0.072) & 68.7 & 0.609 (0.173) & 77.7 \\

20\thinspace000 & 0.2 & 31.7 & 3.8\% & 0.443 (0.097) & 96.7 & 0.411 (0.078) & 93.0 & 0.289 (0.078) & 83.3 & 0.774 (0.189) & 87.6 \\

20\thinspace000 & 0.3 & 37.1 & 4.4\% & 0.542 (0.103) & 99.0 & 0.541 (0.075) & 97.9 & 0.429 (0.079) & 92.9 & 0.844 (0.191) & 93.3 \\

\hline

\end{tabular}

\caption*{\onehalfspacing Mean estimates, mean standard errors, and power of 95\% confidence interval to reject null hypothesis of inverse-variance weighted, weighted median, and MR-Egger regression methods in simulation study for one-sample Mendelian randomization with a positive ($\beta=0.1$) causal effect.}

\end{footnotesize}

\end{center}

%\end{adjustwidth}

\end{minipage}

\end{sidewaystable}

%\setlength{\tabcolsep}{5pt}

\begin{table}[htbp]

\begin{minipage}{\textwidth}

\begin{adjustwidth}{-0.4cm}{-0.4cm}

\begin{center}

\begin{footnotesize}

\centering

\caption{\label{tab:SimScenario1c} \textbf{Results from simulation study in two-sample setting for additional methods}}

\begin{tabular}{c|cc|cc|cc|cc}

\hline

& & & \multicolumn{2}{c|}{Inverse-variance} & \multicolumn{2}{c|}{} & \multicolumn{2}{c}{Inverse-standard error} \\

& & & \multicolumn{2}{c|}{weighted median} & \multicolumn{2}{c|}{Simple median} & \multicolumn{2}{c}{weighted median} \\

\hline

& & Proportion of & Mean estimate & & Mean estimate & & Mean estimate & \\

&$N$ & invalid IVs & (mean SE) & Power & (mean SE) & Power & (mean SE) & Power \\

\hline

& \multicolumn{8}{c}{Null causal effect ($\beta = 0$)} \\

\hline

\multirow{6}{*}{\begin{sideways}Scenario 1\end{sideways}}

& 10\thinspace000 & 0.1 & -0.001 (0.093) & 3.2 & 0.000 (0.106) & 2.5 & 0.000 (0.091) & 3.1 \\

& 10\thinspace000 & 0.2 & 0.001 (0.098) & 4.5 & 0.001 (0.113) & 2.8 & 0.001 (0.096) & 4.4 \\

& 10\thinspace000 & 0.3 & 0.001 (0.103) & 6.2 & 0.001 (0.122) & 4.1 & 0.001 (0.101) & 5.6 \\

& 20\thinspace000 & 0.1 & 0.000 (0.067) & 3.4 & -0.001 (0.074) & 2.9 & 0.000 (0.065) & 3.4 \\

& 20\thinspace000 & 0.2 & 0.001 (0.071) & 4.4 & 0.000 (0.078) & 3.4 & 0.001 (0.069) & 4.1 \\

& 20\thinspace000 & 0.3 & -0.001 (0.075) & 6.4 & 0.001 (0.085) & 4.8 & -0.001 (0.074) & 6.2 \\

\hline

\multirow{6}{*}{\begin{sideways}Scenario 2\end{sideways}}

& 10\thinspace000 & 0.1 & 0.033 (0.093) & 4.9 & 0.042 (0.106) & 3.8 & 0.036 (0.091) & 4.9 \\

& 10\thinspace000 & 0.2 & 0.078 (0.100) & 10.7 & 0.098 (0.117) & 9.3 & 0.084 (0.098) & 11.6 \\

& 10\thinspace000 & 0.3 & 0.139 (0.109) & 21.8 & 0.179 (0.134) & 22.0 & 0.149 (0.108) & 24.2 \\

& 20\thinspace000 & 0.1 & 0.026 (0.067) & 4.9 & 0.033 (0.074) & 4.5 & 0.028 (0.066) & 5.0 \\

& 20\thinspace000 & 0.2 & 0.061 (0.072) & 11.9 & 0.075 (0.082) & 11.9 & 0.066 (0.071) & 13.2 \\

& 20\thinspace000 & 0.3 & 0.115 (0.080) & 25.4 & 0.137 (0.095) & 24.6 & 0.120 (0.080) & 27.9 \\

\hline

\multirow{6}{*}{\begin{sideways}Scenario 3\end{sideways}}

& 10\thinspace000 & 0.1 & 0.145 (0.095) & 29.9 & 0.033 (0.106) & 3.6 & 0.063 (0.092) & 11.1 \\

& 10\thinspace000 & 0.2 & 0.303 (0.097) & 61.3 & 0.074 (0.114) & 8.0 & 0.148 (0.096) & 31.9 \\

& 10\thinspace000 & 0.3 & 0.435 (0.092) & 82.5 & 0.129 (0.121) & 17.7 & 0.258 (0.096) & 58.7 \\

& 20\thinspace000 & 0.1 & 0.131 (0.072) & 32.4 & 0.023 (0.074) & 3.4 & 0.049 (0.067) & 11.1 \\

& 20\thinspace000 & 0.2 & 0.290 (0.075) & 63.8 & 0.054 (0.081) & 7.8 & 0.120 (0.073) & 31.9 \\

& 20\thinspace000 & 0.3 & 0.428 (0.072) & 83.9 & 0.095 (0.089) & 16.7 & 0.218 (0.076) & 58.1 \\

\hline

\hline

& \multicolumn{8}{c}{Positive causal effect ($\beta = 0.1$)} \\

\hline

\multirow{6}{*}{\begin{sideways}Scenario 1\end{sideways}}

& 10\thinspace000 & 0.1 & 0.085 (0.098) & 12.3 & 0.099 (0.112) & 11.1 & 0.091 (0.096) & 13.2 \\

& 10\thinspace000 & 0.2 & 0.088 (0.103) & 13.5 & 0.100 (0.119) & 10.7 & 0.093 (0.101) & 14.5 \\

& 10\thinspace000 & 0.3 & 0.088 (0.109) & 13.4 & 0.101 (0.128) & 11.3 & 0.092 (0.107) & 14.5 \\

& 20\thinspace000 & 0.1 & 0.092 (0.071) & 24.0 & 0.099 (0.078) & 22.6 & 0.095 (0.069) & 26.3 \\

& 20\thinspace000 & 0.2 & 0.093 (0.075) & 24.4 & 0.101 (0.083) & 21.8 & 0.096 (0.073) & 25.9 \\

& 20\thinspace000 & 0.3 & 0.091 (0.079) & 22.6 & 0.101 (0.089) & 20.7 & 0.094 (0.078) & 24.2 \\

\hline

\multirow{6}{*}{\begin{sideways}Scenario 2\end{sideways}}

& 10\thinspace000 & 0.1 & 0.121 (0.099) & 20.9 & 0.143 (0.113) & 20.3 & 0.130 (0.097) & 24.0 \\

& 10\thinspace000 & 0.2 & 0.168 (0.107) & 32.5 & 0.203 (0.125) & 32.8 & 0.180 (0.105) & 37.8 \\

& 10\thinspace000 & 0.3 & 0.232 (0.116) & 47.6 & 0.287 (0.143) & 49.3 & 0.248 (0.116) & 54.7 \\

& 20\thinspace000 & 0.1 & 0.120 (0.071) & 37.2 & 0.135 (0.079) & 38.8 & 0.125 (0.070) & 42.0 \\

& 20\thinspace000 & 0.2 & 0.157 (0.077) & 52.5 & 0.179 (0.088) & 53.4 & 0.165 (0.076) & 58.4 \\

& 20\thinspace000 & 0.3 & 0.213 (0.086) & 66.3 & 0.245 (0.102) & 67.6 & 0.223 (0.086) & 72.7 \\

\hline

\multirow{6}{*}{\begin{sideways}Scenario 3\end{sideways}}

& 10\thinspace000 & 0.1 & 0.238 (0.101) & 48.5 & 0.134 (0.112) & 18.5 & 0.157 (0.097) & 33.5 \\

& 10\thinspace000 & 0.2 & 0.400 (0.103) & 75.8 & 0.178 (0.121) & 28.2 & 0.247 (0.102) & 57.9 \\

& 10\thinspace000 & 0.3 & 0.533 (0.099) & 90.5 & 0.235 (0.129) & 42.5 & 0.359 (0.101) & 77.9 \\

& 20\thinspace000 & 0.1 & 0.229 (0.076) & 63.8 & 0.124 (0.079) & 32.9 & 0.147 (0.071) & 52.7 \\

& 20\thinspace000 & 0.2 & 0.391 (0.079) & 85.0 & 0.158 (0.086) & 43.5 & 0.221 (0.078) & 72.5 \\

& 20\thinspace000 & 0.3 & 0.529 (0.076) & 94.7 & 0.200 (0.095) & 54.7 & 0.323 (0.080) & 86.0 \\

\hline

\end{tabular}

\caption*{\onehalfspacing Mean estimates, mean standard errors, and power of 95\% confidence interval to reject null hypothesis of inverse-variance weighted median (as in main body of paper), simple median, and inverse-standard error weighted median methods in simulation study for two-sample Mendelian randomization with null ($\beta=0$) and positive ($\beta=0.1$) causal effects.}

\end{footnotesize}

\end{center}

\end{adjustwidth}

\end{minipage}

\end{table}

\clearpage

\section*{Web Appendix 4: Example setup and additional information}

Data from Do et al.\ is available in the supplementary material of their paper \citep{do2013}, and can be downloaded from \url{http://dx.doi.org/10.6084/m9.figshare.1116328}. These data are displayed in an interactive graphical format at \url{http://www.phpc.cam.ac.uk/charttest3.html}. The number of genetic variants used in each analysis is: 1) all variants: 73 (\mbox{LDL-c}), 85 (\mbox{HDL-c}), 47 (triglycerides); 2) primary association with target exposure: 61 (\mbox{LDL-c}), 69 (\mbox{HDL-c}), 31 (triglycerides).

Scatter plots for the associations of each pair of lipid fractions are given in Web Figure~\ref{scatters2}. Funnel plots for each of the exposures in turn are given in Web Figure~\ref{funnels}. There is some visual evidence of asymmetry in the funnel plots for \mbox{HDL-c}, a sign that may suggest directional pleiotropy (pleiotropic effects are not balanced on average).

There may be some overlap between participants used in the GLGC and CARDIoGRAM datasets. The studies ADVANCE (505 participants in GLGC; 278 cases, 312 controls, 590 participants in CARDIoGRAM), deCODE (15\thinspace612 participants in GLGC; 6640 cases, 27\thinspace611 controls, 34\thinspace251 participants in CARDIoGRAM), and LURIC (1506 participants in GLGC; 1138 cases, 509 controls, 1647 participants), appear in both papers: total 17\thinspace623 participants in GLGC, 36\thinspace488 participants in CARDIoGRAM \citep{willer2013, deloukas2013}. These studies comprise 9.3\% of the 188,577 participants in GLGC, and 41.9\% of the 86\thinspace995 participants (22\thinspace233 cases and 64\thinspace762 controls) in CARIDoGRAM. Correlation between the genetic association estimates depends on the smaller of these percentages. Hence, there is some possibility for bias in the direction of the observational association due to weak instrument bias in the analysis, although this is unlikely materially affect results as bias is unlikely to be substantial with such limited overlap \citep{pierce2013}.

\begin{figure}[htbp]

\begin{center}

\includegraphics[width=0.98\textwidth]{wmedian_figs/scatters2}

\end{center}

\caption{Scatter plots of genetic associations for each pair of lipid fractions (low-density lipoprotein cholesterol, \mbox{LDL-c}; high-density lipoprotein cholesterol, \mbox{HDL-c}; triglycerides) in turn. Left side: all genetic variants, right side: genetic variants having primary association with the target exposure.} \label{scatters2}

\end{figure}

\begin{figure}[htbp]

\begin{center}

\includegraphics[width=0.98\textwidth]{wmedian_figs/funnels}

\end{center}

\caption{Funnel plots of instrument strength (defined as genetic association with exposure divided by standard error of genetic association with outcome: $\frac{\hat{\gamma}_j}{\sigma_{Yj}}$) against ratio estimates (defined as genetic association with outcome divided by genetic association with exposure: $\frac{\hat{\Gamma}_j}{\hat{\gamma}_j}$) for each genetic variant, and each exposure (low-density lipoprotein cholesterol, \mbox{LDL-c}; high-density lipoprotein cholesterol, \mbox{HDL-c}; triglycerides) in turn. Left side: all genetic variants, right side: genetic variants having primary association with the target exposure.} \label{funnels}

\end{figure}

\clearpage

\putbib[masterref]

\end{bibunit}

\end{document}
