# Supplementary material for: Consistent Estimation in Mendelian Randomization with Some Invalid Instruments Using a Weighted Median Estimator
Source: Genet Epidemiol. 2016 Apr 7;40(4):304–14. doi: 10.1002/gepi.21965 (PMC4849733; doi:10.1002/gepi.21965)
Supplement: Supplementary file 2 — Web Table A1: Results from simulation study in one‐sample setting with null causal effect Web Table A2: Results from simulation study in one‐sample setting with positive causal effect Web Table A3: Results from simulation study in two‐sample setting for addi‐ tional methods Web Figure A1: Scatter plots of genetic associations for each pair of lipid fractions (low‐density lipoprotein cholesterol, LDL‐c; high‐density lipoprotein cholesterol, HDL‐ c; triglycerides) in turn. Left side: all genetic variants, right side: genetic variants having primary association with the target exposure. Web Figure A2: Funnel plots of instrument strength (defined as genetic association with exposure divided by standard error of genetic association with outcome: γ^jσYj) against ratio estimates (defined as genetic association with outcome divided by genetic association with exposure: Γ^jγ^j) for each genetic variant, and each exposure (low‐ density lipoprotein cholesterol, LDL‐c; high‐density lipoprotein cholesterol, HDL‐c; triglycerides) in turn. Left side: all genetic variants, right side: genetic variants having primary association with the target exposure. [file GEPI-40-304-s002.pdf]

Web Appendix 1: Some technical remarks

The concept of a weighted median is one that goes back to the 19th century [Edgeworth, 1887, 1888]. However, there is no universally agreed method for calculating a weighted median (or an arbitrary percentile from a finite sample; see `https://en.wikipedia.org/w/index.php?title=Percentile\&oldid=668900109` for a non-technical overview). We have settled with the weighted percentile definition in this paper after examining the performance of a number of different methods. An alternative approach would be to define a weighted empirical distribution and take the median from this distribution. The cumulative distribution function $F(p), 0 \leq p \leq 1$ for the empirical distribution would be defined as:

$$F(p) = \hat{\beta}_j, \text{ for } j \text{ such that } s_{j-1} < p < s_j \tag{1}$$

where $s_j = \sum_{k=1}^{j} w_k$ is the cumulative sum of weights up to the jth genetic variant. The weighted median estimate according to the empirical distribution method would be $F(0.5)$. Code for the empirical distribution implementation of a weighted median method (as well as code for the weighted percentile method used in this paper) is given in Web Appendix 2. For the simple median estimator, this difference is moot.

The advantage of the empirical distribution method is that it is always consistent if over 50% of the weight in the analysis comes from valid IVs. In the weighted percentile method advocated in this paper, there are corner cases that can be constructed in which this is not true. For instance, if $w_1 = 0.3$, $w_2 = 0.15$, $w_3 = 0.08$, and $w_4 = 0.47$, and the only first three variants are valid IVs, then the weighted percentile method will not be consistent, as it will extrapolate between the third and fourth ratio estimates. However, provided that there are a moderately large number of variants (as will normally be the case in applied practice) and provided that a large proportion of the weight is not concentrated in one single variant, this is unlikely to be a serious issue. If the greatest weight is 10%, consistency in the weighted percentile method is guaranteed provided that 55% of the weight comes from valid IVs. There are

several advantages of using the weighted percentile approach, which in our opinion outweigh this technical deficiency. By extrapolating, the weighted median estimate is not constrained to take the value of one of the ratio estimates. This gives greater stability to the point estimate. Bootstrap confidence intervals are also more reliable, as the distribution of estimates from each iteration of the bootstrap will be more continuous.

In any event, the aim of this paper is not to advocate a single weighted median method as superior to other weighted median methods (in the same way as we would not argue for reliance on any single sensitivity analysis for a Mendelian randomization investigation). We look forward to further technical developments in finding an optimal weighted median method.

Web Appendix 2: Software code

We provide R code for implementing the weighted median approach using the weighted percentile function (as performed in the simulations and example of the paper).

```
weighted.median <- function(betaIV.in, weights.in) {
  betaIV.order  = betaIV.in[order(betaIV.in)]
  weights.order = weights.in[order(betaIV.in)]
  weights.sum   = cumsum(weights.order)-0.5*weights.order
  weights.sum   = weights.sum/sum(weights.order)
        below   = max(which(weights.sum<0.5))
  weighted.est  = betaIV.order[below] + (betaIV.order[below+1]-betaIV.order[below])*
                  (0.5-weights.sum[below])/(weights.sum[below+1]-weights.sum[below])
  return(weighted.est) }

weighted.median.boot = function(betaXG.in, betaYG.in, sebetaXG.in, sebetaYG.in, weights.in){
med = NULL
for(i in 1:1000){
 betaXG.boot = rnorm(length(betaXG.in), mean=betaXG.in, sd=sebetaXG.in)
 betaYG.boot = rnorm(length(betaYG.in), mean=betaYG.in, sd=sebetaYG.in)
 betaIV.boot  = betaYG.boot/betaXG.boot
 med[i] = weighted.median(betaIV.boot, weights.in)
 }
return(sd(med)) }

betaIV       = betaYG/betaXG                          # ratio estimates
weights      = (sebetaYG/betaXG)^-2                   # inverse-variance weights
betaIVW      = sum(betaYG*betaXG*sebetaYG^-2)/sum(betaXG^2*sebetaYG^-2)
                                                      # IVW estimate
penalty      = pchisq(weights*(betaIV-betaIVW)^2, df=1, lower.tail=FALSE)
pen.weights  = weights*pmin(1, penalty*20)            # penalized weights

betaWM       = weighted.median(betaIV, weights)       # weighted median estimate
sebetaWM     = weighted.median.boot(betaXG, betaYG, sebetaXG, sebetaYG, weights)
                                                      # standard error
betaPWM      = weighted.median(betaIV, pen.weights)   # penalized weighted median estimate
sebetaPWM    = weighted.median.boot(betaXG, betaYG, sebetaXG, sebetaYG, pen.weights)
                                                      # standard error
```

We found that the bootstrap confidence interval (that is, the 2.5th to the 97.5th percentile of the bootstrap estimates) gives poor coverage, tending to be too conservative. However, the bootstrap standard error (the standard deviation of the bootstrap estimates) gave more reasonable coverage using a normal approximation (estimate

$\pm 1.96 \times$ standard error) to form a 95% confidence interval.

An alternative interpretation of a weighted median estimator based on an empirical distribution function is discussed in Web Appendix 1.

```
weighted.median.empirical <- function(betaIV.in, weights.in) {
  betaIV.order  = betaIV.in[order(betaIV.in)]
  weights.order = weights.in[order(betaIV.in)]
  weights.sum   = cumsum(weights.order)
  weights.sum   = weights.sum/sum(weights.order)
  which.below   = max(which(weights.sum<0.5))
  return(betaIV.order[which.below+1]) }

betaWME       = weighted.median.empirical(betaIV, weights)
                          # alternative weighted median estimate
```

An inverse-standard error weighted can be implemented by replacing the inverse-variance weights with:

```
weights      = (sebetaYG/betaXG)^-1           # inverse-standard error weights
```

The IVW and MR-Egger regression approaches used in this paper can be obtained using the following code:

```
betaIVW     = summary(lm(betaYG~betaXG-1, weights=sebetaYG^-2))$coef[1,1]
sebetaIVW   = summary(lm(betaYG~betaXG-1, weights=sebetaYG^-2))$coef[1,2]/
              min(summary(lm(betaYG~betaXG-1, weights=sebetaYG^-2))$sigma, 1)
betaEGGER   = summary(lm(betaYG~betaXG, weights=sebetaYG^-2))$coef[2,1]
sebetaEGGER = summary(lm(betaYG~betaXG, weights=sebetaYG^-2))$coef[2,2]/
              min(summary(lm(betaYG~betaXG, weights=sebetaYG^-2))$sigma, 1)
```

This differs slightly from the code originally given in Bowden et al. [Bowden et al., 2015] (although it is similar to that used in the simulation study of Bowden et al.), as it allows for heterogeneity in the causal effects from different IVs via not constraining the residual standard error to be 1. If the estimate of the residual standard error is less than 1, then we divide the standard errors of the coefficients by this estimate to ensure that they are not overly precise. However, if it is greater than 1, then we do not divide by the residual standard error, as this results in over-precision when there is true heterogeneity between the causal effects identified by different IVs. In particular, dividing by the residual standard error in the simulation study resulted

in substantial under-coverage (as there was substantial heterogeneity in the causal estimates from each genetic variant due to the invalid IVs). However, if there is substantial heterogeneity in practice, this would call into question the validity of the IVW method, and a stricter filtering as to which of the genetic variants to include in the analysis should be applied.

Coverage for each method was based on a 95% confidence interval (estimate $\pm 1.96 \times$ standard error). This is slightly too narrow in the case of the IVW and MR-Egger methods, as a t-distribution should be used. With 25 genetic variants, this would lead to a confidence interval of estimate $\pm 2.06 \times$ standard error for the IVW method (t-distribution on 24 degrees of freedom), and estimate $\pm 2.07 \times$ standard error for the MR-Egger method (23 degrees of freedom). We used a normal approximation for consistency between the methods. Consequently, coverage under the null in the two-sample setting (Table II) was slightly above the nominal 5% level, but not greatly so.

Web Appendix 3: Simulation setup and additional results

The data-generating model was as follows:

$$U_i = \sum_{j=1}^{J} \phi_j G_{ij} + \epsilon_i^U$$
$$X_i = \sum_{j=1}^{J} \gamma_j G_{ij} + U_i + \epsilon_i^X$$
$$Y_i = \sum_{j=1}^{J} \alpha_j G_{ij} + \beta X_i + U_i + \epsilon_i^Y.$$

for participants indexed by $i = 1, \ldots, N$, and genetic variants indexed by $j = 1, \ldots, J$. The error terms ϵ_i^U, ϵ_i^X, and ϵ_i^Y were each drawn independently from standard normal distributions. The genetic effects on the exposure γ_j are drawn from a uniform distribution between 0.03 and 0.1. Pleiotropic effects α_j and ϕ_j were set to zero if the genetic variant was a valid instrumental variable. Otherwise (with probability 0.1, 0.2, or 0.3):

1. In Scenario 1 (balanced pleiotropy, InSIDE satisfied), the α_j parameter was drawn from a uniform distribution between -0.2 and 0.2.

2. In Scenario 2 (directional pleiotropy, InSIDE satisfied), the α_j parameter was drawn from a uniform distribution between 0 and 0.2.

3. In Scenario 3 (directional pleiotropy, InSIDE not satisfied), the ϕ_j parameter was drawn from a uniform distribution between -0.2 and 0.2.

The causal effect of the exposure on the outcome was either $\beta_X = 0$ (null causal effect) or $\beta_X = 0.1$ (positive causal effect). A total of 10 000 simulated datasets were generated for sample sizes of $N = 10\,000$ and 20 participants. Only the summary data, that is genetic associations with the exposure and with the outcome and their standard errors as estimated by univariate regression on the genetic variants in turn,

were used by the analysis methods. In the two-sample setting, data were generated on $2N$ participants, and genetic associations with the exposure were estimated in the first N participants, and genetic associations with the outcome in the second N participants. The Monte Carlo standard error for the mean estimates was 0.002 or less, and for the power was less than 0.5% in all cases.

The standard errors used in the IVW and MR-Egger methods in this simulation correspond to those used in the simulations of Bowden et al. [Bowden et al., 2015], but differ from those previously recommended for use in the IVW [Johnson, 2013; Burgess et al., 2013] and originally recommended for the MR-Egger regression method (see Web Appendix 2 above). The reason is that the IVW method corresponds to a fixed-effect meta-analysis of the ratio estimates from each genetic variant. In the examples of this paper, there is heterogeneity in the ratio estimates, and so a fixed-effect analysis is inappropriate, and leads to overly precise confidence intervals and inflated Type 1 error rates. A conventional random-effects meta-analysis would not be wise, as the random-effects estimate upweights outlying estimates, inflating the influence of pleiotropic genetic variants on the analysis. Hence we have reached a compromise, similar to that suggested by Copas et al. [Henmi and Copas, 2010], that we take the point estimate from a fixed-effect analysis, but allow confidence intervals to be inflated by heterogeneity as per a random-effects analysis. This is achieved by performing a weighted regression as described in the description of MR-Egger regression, but not setting the residual standard error in the regression to be 1, as is recommended in meta-analysis to correspond to a fixed-effect analysis (unless the estimate of the residual standard error is less than 1, in which case we divide the standard errors of the coefficients by the estimate of the residual standard error to avoid over-precision). This is equivalent to a multiplicative random-effects model [Thompson and Sharp, 1999].

Results in a two-sample setting are presented in the main manuscript. In a one-sample setting, estimates from the two-stage least squares method (individual-level data) and the IVW method (summary data) are known to be biased in the direction

of the observational association; this is known as weak instrument bias. In a two-sample setting, estimates are less biased, and any bias is in the direction of the null (conservative bias) [Pierce and Burgess, 2013]. Scenario 1 of Table III suggests that the same is true for the median-based and MR-Egger methods, with some bias in the direction of the null in a two-sample setting with balanced pleiotropy. When pleiotropy is unbalanced, bias due to pleiotropy seems to be stronger than any attenuation due to weak instruments, at least for the parameters considered in this paper.

Web Tables A1 (null causal effect) and A2 (positive causal effect) present results in a one-sample setting. In a one-sample setting, all of the methods are affected by weak instrument bias. Estimates under the null are biased in the direction of the observational confounded association, and Type 1 error rates are inflated. Estimates from MR-Egger regression are particularly affected, with substantially more bias than estimates from the other methods. The weighted median methods have better coverage properties under the null than those of the IVW method, although not as good as the MR-Egger method in Scenario 2 (but better in Scenarios 1 and 3). Bias in all methods reduces as the sample size increases.

Web Table A3 presents results in a two-sample setting for the simple median method, and for an inverse-standard error weighted median method, for which the unstandardized weights are:

$$w'_j = \frac{\hat{\gamma}_j}{\sigma_{Yj}}$$

Results from the (inverse-variance) weighted median method considered above are reproduced from Tables II and III for comparison. Estimates from the simple median method are less precise than those from the weighted median methods, but the differences are not substantial in this simulation setting. Bias and coverage under the null in Scenarios 1 and 2 are similar to those from the (inverse-variance) weighted median method, but in Scenario 3 the bias and coverage properties are much improved. However, this is due to an artefact of the simulation, as invalid genetic variants are stronger on average than valid instruments, as they are additionally associated with the risk factor through the confounder. Hence, it is not clear that the simple median

method should be preferred to a weighted median method on the basis of these simulations, but it does provide another valuable sensitivity analysis that gives consistent estimates under a slightly different assumption (simple median method assumes that 50% of variants are valid instrumental variables, weighted median methods assume that 50% of the weight comes from valid instrumental variables).

Estimates from the inverse-standard error weighted median method are slightly more precise than those from the inverse-variance weighted median method. Coverage of the inverse-standard error method under the null is slightly worse in Scenario 2, but better in Scenario 3. Power to detect a causal effect is better in Scenario 1. However, we do not expect these results to be fully generalizable, and hence would not advocate one implementation of the method over others. In fact, both the simple median and inverse-standard error method reported a causal effect of HDL-c on CAD risk in the applied example, suggesting that the inverse-variance weighted median method would be preferred in this single case.

Web Table A1: **Results from simulation study in one-sample setting with null causal effect**

				Inverse-variance weighted		Weighted median		Penalized weighted median		MR-Egger regression	
N	Proportion of invalid IVs	F	R^2	Mean estimate (mean SE)	Power	Mean estimate (mean SE)	Power	Mean estimate (mean SE)	Power	Mean estimate (mean SE)	Power
Scenario 1. Balanced pleiotropy, InSIDE assumption satisfied											
10 000	0.1	10.7	2.6%	0.045 (0.114)	8.7	0.066 (0.092)	9.0	0.063 (0.093)	8.4	0.233 (0.284)	18.6
10 000	0.2	10.7	2.6%	0.045 (0.153)	7.6	0.066 (0.097)	9.9	0.061 (0.097)	8.7	0.229 (0.383)	12.4
10 000	0.3	10.7	2.6%	0.042 (0.184)	7.1	0.063 (0.103)	10.3	0.057 (0.103)	8.6	0.224 (0.463)	9.9
20 000	0.1	20.5	2.5%	0.022 (0.106)	6.3	0.036 (0.067)	6.5	0.033 (0.067)	6.0	0.165 (0.300)	13.7
20 000	0.2	20.5	2.5%	0.024 (0.150)	6.3	0.037 (0.070)	8.4	0.033 (0.071)	7.8	0.180 (0.425)	9.7
20 000	0.3	20.5	2.5%	0.020 (0.183)	6.4	0.034 (0.075)	9.2	0.030 (0.076)	8.6	0.165 (0.522)	7.7
Scenario 2. Directional pleiotropy, InSIDE assumption satisfied											
10 000	0.1	10.7	2.6%	0.173 (0.110)	30.4	0.099 (0.093)	15.7	0.085 (0.093)	12.4	0.248 (0.275)	19.6
10 000	0.2	10.7	2.6%	0.300 (0.142)	55.4	0.139 (0.100)	25.7	0.121 (0.100)	20.1	0.257 (0.358)	14.2
10 000	0.3	10.7	2.6%	0.430 (0.165)	78.4	0.199 (0.109)	40.1	0.190 (0.112)	35.0	0.280 (0.417)	12.1
20 000	0.1	20.5	2.5%	0.160 (0.104)	24.3	0.063 (0.067)	12.7	0.058 (0.068)	11.5	0.177 (0.295)	13.8
20 000	0.2	20.5	2.5%	0.295 (0.140)	55.3	0.098 (0.072)	24.2	0.106 (0.077)	24.6	0.189 (0.396)	10.0
20 000	0.3	20.5	2.5%	0.431 (0.165)	79.9	0.146 (0.080)	39.2	0.197 (0.095)	44.9	0.194 (0.465)	8.8
Scenario 3. Directional pleiotropy, InSIDE assumption not satisfied											
10 000	0.1	13.6	3.3%	0.222 (0.089)	61.3	0.202 (0.094)	45.1	0.118 (0.092)	23.1	0.516 (0.180)	69.5
10 000	0.2	16.3	3.9%	0.347 (0.101)	84.0	0.339 (0.094)	72.7	0.226 (0.094)	48.8	0.650 (0.190)	82.3
10 000	0.3	19.2	4.6%	0.445 (0.106)	94.9	0.458 (0.090)	89.0	0.360 (0.092)	73.9	0.723 (0.191)	90.0
20 000	0.1	26.0	3.1%	0.206 (0.082)	60.2	0.157 (0.071)	42.6	0.084 (0.069)	20.6	0.509 (0.173)	69.2
20 000	0.2	31.7	3.8%	0.343 (0.097)	85.2	0.311 (0.073)	72.6	0.193 (0.075)	48.1	0.673 (0.189)	83.4
20 000	0.3	37.1	4.4%	0.442 (0.103)	95.3	0.441 (0.071)	89.0	0.332 (0.076)	74.3	0.744 (0.191)	90.3

Mean estimates, mean standard errors, and power of 95% confidence interval to reject null hypothesis of inverse-variance weighted, weighted median, and MR-Egger regression methods in simulation study for one-sample Mendelian randomization with a null ($\beta = 0$) causal effect.

[0]Abbreviation: SE, standard error

Web Table A2: **Results from simulation study in one-sample setting with positive causal effect**

				Inverse-variance weighted		Weighted median		Penalized weighted median		MR-Egger regression	
N	Proportion of invalid IVs	F	R^2	Mean estimate (mean SE)	Power	Mean estimate (mean SE)	Power	Mean estimate (mean SE)	Power	Mean estimate (mean SE)	Power
Scenario 1. Balanced pleiotropy, InSIDE assumption satisfied											
10 000	0.1	10.7	2.6%	0.145 (0.114)	33.4	0.166 (0.097)	40.9	0.164 (0.097)	39.6	0.333 (0.284)	29.6
10 000	0.2	10.7	2.6%	0.146 (0.153)	20.8	0.166 (0.102)	37.7	0.161 (0.102)	36.0	0.329 (0.383)	18.1
10 000	0.3	10.7	2.6%	0.143 (0.184)	14.6	0.163 (0.108)	35.1	0.158 (0.108)	32.7	0.324 (0.463)	13.7
20 000	0.1	20.5	2.5%	0.122 (0.106)	33.4	0.136 (0.070)	50.9	0.133 (0.070)	49.3	0.265 (0.300)	23.8
20 000	0.2	20.5	2.5%	0.124 (0.150)	18.5	0.137 (0.074)	47.7	0.133 (0.074)	46.2	0.280 (0.425)	13.9
20 000	0.3	20.5	2.5%	0.120 (0.183)	13.1	0.134 (0.078)	43.2	0.130 (0.080)	40.9	0.266 (0.521)	10.2
Scenario 2. Directional pleiotropy, InSIDE assumption satisfied											
10 000	0.1	10.7	2.6%	0.273 (0.110)	75.5	0.199 (0.098)	54.4	0.183 (0.098)	47.4	0.348 (0.275)	31.5
10 000	0.2	10.7	2.6%	0.400 (0.142)	87.2	0.240 (0.106)	64.0	0.217 (0.105)	54.7	0.357 (0.358)	21.2
10 000	0.3	10.7	2.6%	0.530 (0.165)	95.6	0.299 (0.116)	74.8	0.282 (0.117)	67.7	0.380 (0.417)	17.5
20 000	0.1	20.5	2.5%	0.260 (0.104)	79.5	0.163 (0.071)	66.0	0.156 (0.071)	60.6	0.277 (0.295)	24.1
20 000	0.2	20.5	2.5%	0.395 (0.140)	90.7	0.198 (0.076)	77.0	0.200 (0.080)	73.0	0.289 (0.396)	14.8
20 000	0.3	20.5	2.5%	0.531 (0.164)	96.9	0.246 (0.085)	84.3	0.286 (0.098)	83.0	0.294 (0.465)	11.9
Scenario 3. Directional pleiotropy, InSIDE assumption not satisfied											
10 000	0.1	13.6	3.3%	0.322 (0.089)	87.9	0.302 (0.100)	74.0	0.218 (0.097)	56.5	0.617 (0.180)	78.9
10 000	0.2	16.3	3.9%	0.447 (0.101)	95.6	0.439 (0.100)	89.6	0.326 (0.099)	75.1	0.751 (0.190)	87.8
10 000	0.3	19.2	4.6%	0.545 (0.106)	98.8	0.558 (0.096)	96.0	0.461 (0.097)	88.3	0.823 (0.191)	93.4
20 000	0.1	26.0	3.1%	0.306 (0.082)	89.9	0.258 (0.075)	81.8	0.181 (0.072)	68.7	0.609 (0.173)	77.7
20 000	0.2	31.7	3.8%	0.443 (0.097)	96.7	0.411 (0.078)	93.0	0.289 (0.078)	83.3	0.774 (0.189)	87.6
20 000	0.3	37.1	4.4%	0.542 (0.103)	99.0	0.541 (0.075)	97.9	0.429 (0.079)	92.9	0.844 (0.191)	93.3

Mean estimates, mean standard errors, and power of 95% confidence interval to reject null hypothesis of inverse-variance weighted, weighted median, and MR-Egger regression methods in simulation study for one-sample Mendelian randomization with a positive ($\beta = 0.1$) causal effect.

Web Table A3: **Results from simulation study in two-sample setting for additional methods**

	N	Proportion of invalid IVs	Inverse-variance weighted median: Mean estimate (mean SE)	Power	Simple median: Mean estimate (mean SE)	Power	Inverse-standard error weighted median: Mean estimate (mean SE)	Power
			**Null causal effect ($\beta = 0$)**					
Scenario 1	10 000	0.1	-0.001 (0.093)	3.2	0.000 (0.106)	2.5	0.000 (0.091)	3.1
	10 000	0.2	0.001 (0.098)	4.5	0.001 (0.113)	2.8	0.001 (0.096)	4.4
	10 000	0.3	0.001 (0.103)	6.2	0.001 (0.122)	4.1	0.001 (0.101)	5.6
	20 000	0.1	0.000 (0.067)	3.4	-0.001 (0.074)	2.9	0.000 (0.065)	3.4
	20 000	0.2	0.001 (0.071)	4.4	0.000 (0.078)	3.4	0.001 (0.069)	4.1
	20 000	0.3	-0.001 (0.075)	6.4	0.001 (0.085)	4.8	-0.001 (0.074)	6.2
Scenario 2	10 000	0.1	0.033 (0.093)	4.9	0.042 (0.106)	3.8	0.036 (0.091)	4.9
	10 000	0.2	0.078 (0.100)	10.7	0.098 (0.117)	9.3	0.084 (0.098)	11.6
	10 000	0.3	0.139 (0.109)	21.8	0.179 (0.134)	22.0	0.149 (0.108)	24.2
	20 000	0.1	0.026 (0.067)	4.9	0.033 (0.074)	4.5	0.028 (0.066)	5.0
	20 000	0.2	0.061 (0.072)	11.9	0.075 (0.082)	11.9	0.066 (0.071)	13.2
	20 000	0.3	0.115 (0.080)	25.4	0.137 (0.095)	24.6	0.120 (0.080)	27.9
Scenario 3	10 000	0.1	0.145 (0.095)	29.9	0.033 (0.106)	3.6	0.063 (0.092)	11.1
	10 000	0.2	0.303 (0.097)	61.3	0.074 (0.114)	8.0	0.148 (0.096)	31.9
	10 000	0.3	0.435 (0.092)	82.5	0.129 (0.121)	17.7	0.258 (0.096)	58.7
	20 000	0.1	0.131 (0.072)	32.4	0.023 (0.074)	3.4	0.049 (0.067)	11.1
	20 000	0.2	0.290 (0.075)	63.8	0.054 (0.081)	7.8	0.120 (0.073)	31.9
	20 000	0.3	0.428 (0.072)	83.9	0.095 (0.089)	16.7	0.218 (0.076)	58.1
			**Positive causal effect ($\beta = 0.1$)**					
Scenario 1	10 000	0.1	0.085 (0.098)	12.3	0.099 (0.112)	11.1	0.091 (0.096)	13.2
	10 000	0.2	0.088 (0.103)	13.5	0.100 (0.119)	10.7	0.093 (0.101)	14.5
	10 000	0.3	0.088 (0.109)	13.4	0.101 (0.128)	11.3	0.092 (0.107)	14.5
	20 000	0.1	0.092 (0.071)	24.0	0.099 (0.078)	22.6	0.095 (0.069)	26.3
	20 000	0.2	0.093 (0.075)	24.4	0.101 (0.083)	21.8	0.096 (0.073)	25.9
	20 000	0.3	0.091 (0.079)	22.6	0.101 (0.089)	20.7	0.094 (0.078)	24.2
Scenario 2	10 000	0.1	0.121 (0.099)	20.9	0.143 (0.113)	20.3	0.130 (0.097)	24.0
	10 000	0.2	0.168 (0.107)	32.5	0.203 (0.125)	32.8	0.180 (0.105)	37.8
	10 000	0.3	0.232 (0.116)	47.6	0.287 (0.143)	49.3	0.248 (0.116)	54.7
	20 000	0.1	0.120 (0.071)	37.2	0.135 (0.079)	38.8	0.125 (0.070)	42.0
	20 000	0.2	0.157 (0.077)	52.5	0.179 (0.088)	53.4	0.165 (0.076)	58.4
	20 000	0.3	0.213 (0.086)	66.3	0.245 (0.102)	67.6	0.223 (0.086)	72.7
Scenario 3	10 000	0.1	0.238 (0.101)	48.5	0.134 (0.112)	18.5	0.157 (0.097)	33.5
	10 000	0.2	0.400 (0.103)	75.8	0.178 (0.121)	28.2	0.247 (0.102)	57.9
	10 000	0.3	0.533 (0.099)	90.5	0.235 (0.129)	42.5	0.359 (0.101)	77.9
	20 000	0.1	0.229 (0.076)	63.8	0.124 (0.079)	32.9	0.147 (0.071)	52.7
	20 000	0.2	0.391 (0.079)	85.0	0.158 (0.086)	43.5	0.221 (0.078)	72.5
	20 000	0.3	0.529 (0.076)	94.7	0.200 (0.095)	54.7	0.323 (0.080)	86.0

Mean estimates, mean standard errors, and power of 95% confidence interval to reject null hypothesis of inverse-variance weighted median (as in main body of paper), simple median, and inverse-standard error weighted median methods in simulation study for two-sample Mendelian randomization with null ($\beta = 0$) and positive ($\beta = 0.1$) causal effects.

Web Appendix 4: Example setup and additional information

Data from Do et al. is available in the supplementary material of their paper [Do et al., 2013], and can be downloaded from `http://dx.doi.org/10.6084/m9.figshare.1116328`. These data are displayed in an interactive graphical format at `http://www.phpc.cam.ac.uk/charttest3.html`. The number of genetic variants used in each analysis is: 1) all variants: 73 (LDL-c), 85 (HDL-c), 47 (triglycerides); 2) primary association with target exposure: 61 (LDL-c), 69 (HDL-c), 31 (triglycerides).

Scatter plots for the associations of each pair of lipid fractions are given in Web Figure A1. Funnel plots for each of the exposures in turn are given in Web Figure A2. There is some visual evidence of asymmetry in the funnel plots for HDL-c, a sign that may suggest directional pleiotropy (pleiotropic effects are not balanced on average).

There may be some overlap between participants used in the GLGC and CARDIoGRAM datasets. The studies ADVANCE (505 participants in GLGC; 278 cases, 312 controls, 590 participants in CARDIoGRAM), deCODE (15 612 participants in GLGC; 6640 cases, 27 611 controls, 34 251 participants in CARDIoGRAM), and LURIC (1506 participants in GLGC; 1138 cases, 509 controls, 1647 participants), appear in both papers: total 17 623 participants in GLGC, 36 488 participants in CARDIoGRAM [Global Lipids Genetics Consortium, 2013; CARDIoGRAMplusC4D Consortium, 2013]. These studies comprise 9.3% of the 188,577 participants in GLGC, and 41.9% of the 86 995 participants (22 233 cases and 64 762 controls) in CARIDoGRAM. Correlation between the genetic association estimates depends on the smaller of these percentages. Hence, there is some possibility for bias in the direction of the observational association due to weak instrument bias in the analysis, although this is unlikely materially affect results as bias is unlikely to be substantial with such limited overlap [Pierce and Burgess, 2013].

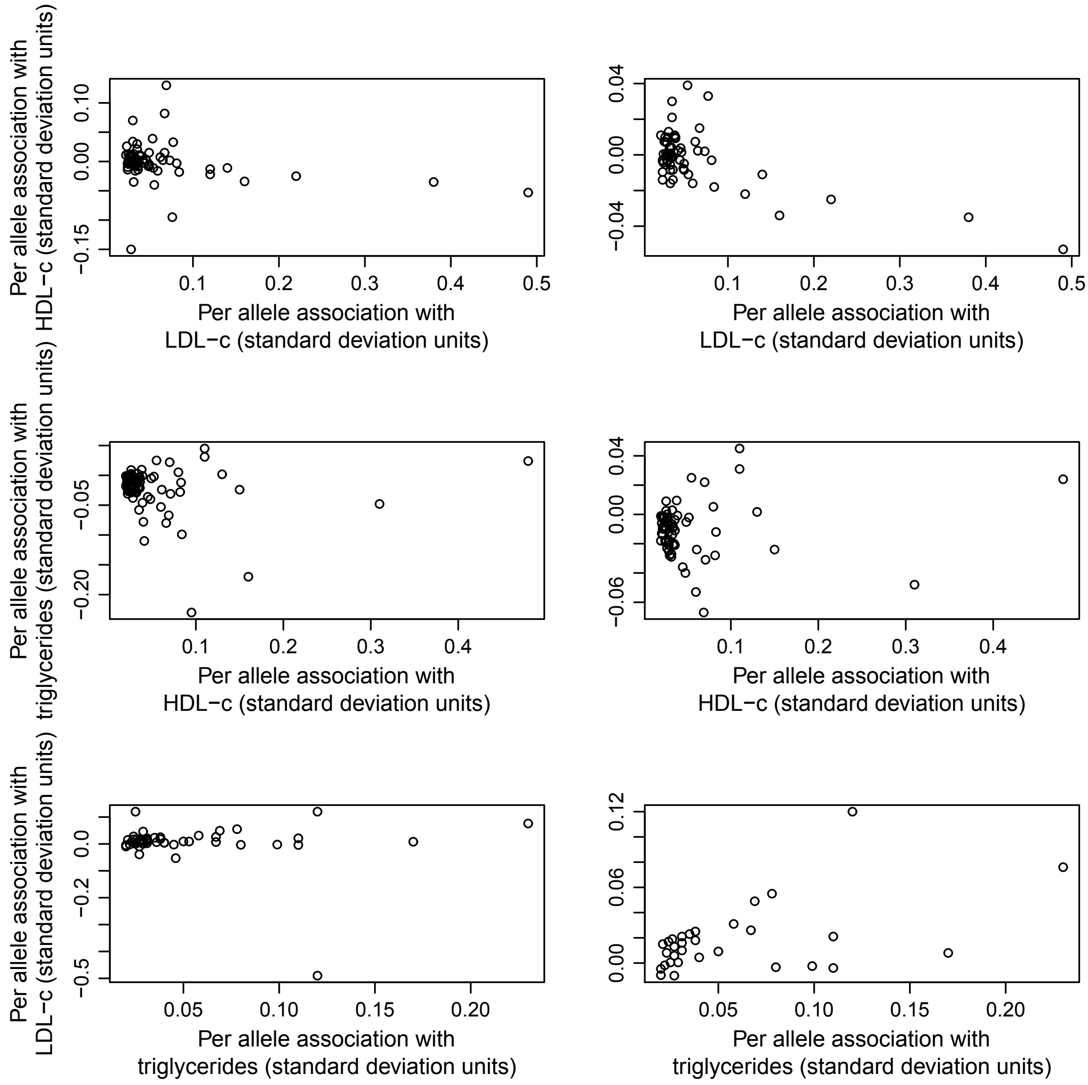

Web Figure A1: Scatter plots of genetic associations for each pair of lipid fractions (low-density lipoprotein cholesterol, LDL-c; high-density lipoprotein cholesterol, HDL-c; triglycerides) in turn. Left side: all genetic variants, right side: genetic variants having primary association with the target exposure.

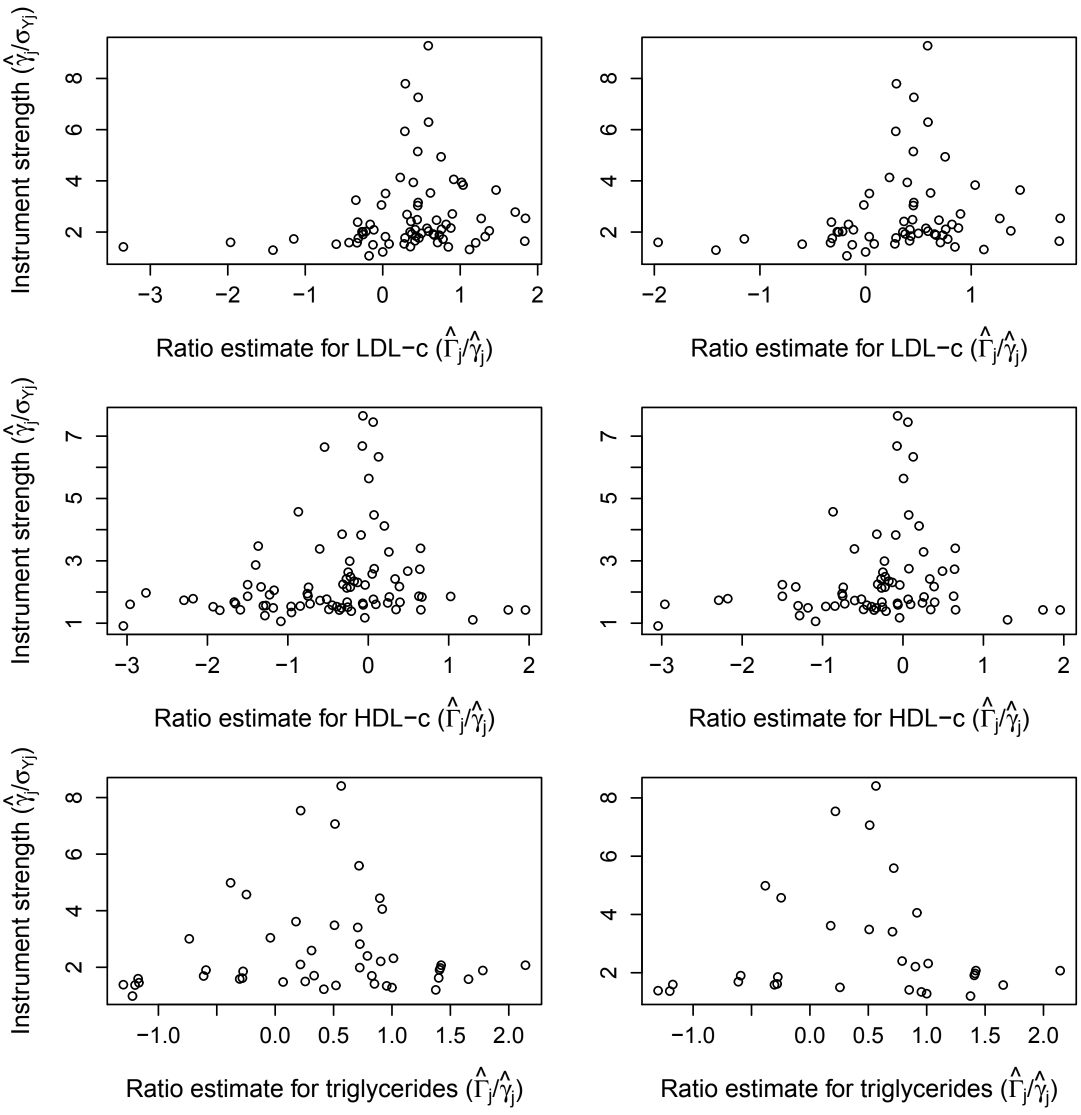

Web Figure A2: Funnel plots of instrument strength (defined as genetic association with exposure divided by standard error of genetic association with outcome: $\frac{\hat{\gamma}_j}{\sigma_{Yj}}$) against ratio estimates (defined as genetic association with outcome divided by genetic association with exposure: $\frac{\hat{\Gamma}_j}{\hat{\gamma}_j}$) for each genetic variant, and each exposure (low-density lipoprotein cholesterol, LDL-c; high-density lipoprotein cholesterol, HDL-c; triglycerides) in turn. Left side: all genetic variants, right side: genetic variants having primary association with the target exposure.

References

Bowden, J., Davey Smith, G., and Burgess, S. 2015. Mendelian randomization with invalid instruments: effect estimation and bias detection through Egger regression. *International Journal of Epidemiology*, 44(2):512–525.

Burgess, S., Butterworth, A., and Thompson, S. 2013. Mendelian randomization analysis with multiple genetic variants using summarized data. *Genetic Epidemiology*, 37(7):658–665.

CARDIoGRAMplusC4D Consortium 2013. Large-scale association analysis identifies new risk loci for coronary artery disease. *Nature Genetics*, 45(1):25–33.

Do, R., Willer, C. J., Schmidt, E. M., et al. 2013. Common variants associated with plasma triglycerides and risk for coronary artery disease. *Nature Genetics*, 45:1345–1352.

Edgeworth, F. Y. 1887. On observations relating to several quantities. *Hermathena*, 6(13):279–285.

Edgeworth, F. Y. 1888. On a new method of reducing observations relating to several quantities. *Philosophical Magazine*, 25(154):184–191.

Global Lipids Genetics Consortium 2013. Discovery and refinement of loci associated with lipid levels. *Nature Genetics*, 45:1274–1283.

Henmi, M. and Copas, J. B. 2010. Confidence intervals for random effects meta-analysis and robustness to publication bias. *Statistics in Medicine*, 29(29):2969–2983.

Johnson, T. 2013. Efficient calculation for multi-SNP genetic risk scores. Technical report, The Comprehensive R Archive Network. Available at http://cran.r-project.org/web/packages/gtx/vignettes/ashg2012.pdf [last accessed 2014/11/19].

Pierce, B. and Burgess, S. 2013. Efficient design for Mendelian randomization studies: subsample and two-sample instrumental variable estimators. *American Journal of Epidemiology*, 178(7):1177–1184.

Thompson, S. and Sharp, S. 1999. Explaining heterogeneity in meta-analysis: a comparison of methods. *Statistics in Medicine*, 18(20):2693–2708.
