# Supplementary material for: Consistent Estimation in Mendelian Randomization with Some Invalid Instruments Using a Weighted Median Estimator
Source: Genet Epidemiol. 2016 Apr 7;40(4):304–14. doi: 10.1002/gepi.21965 (PMC4849733; doi:10.1002/gepi.21965)
Supplement: Supplementary file 3 — Figure 1: Illustrative diagram representing the hypothesized relationships between ge‐ netic variant Gj , exposure X, disease Y and confounders U when Gj is a valid instrumental variable (IV). Crosses indicate violations of assumptions IV2 and IV3 that potentially lead to invalid inferences from conventional methods. Figure 2: Fictional example of a Mendelian randomization analysis with 10 genetic variants – 6 valid instrumental variables (hollow circles) and 4 invalid instrumental variables (solid circles) for finite sample size (left) and infinite sample size (right) showing inverse variance weighted (IVW, solid line) and simple median (dashed line) estimates compared with the true causal effect (dotted line). Figure 3: Empirical distribution functions of ordered ratio instrumental variable es‐ timates (β^j) used for calculation of the simple median estimate (black) and two weighted median estimates (shown in red and blue) using the weights given in Table I. Figure 4: Scatter plots of genetic associations with the outcome (coronary artery disease risk, CAD) against genetic associations with the exposure (low‐density lipoprotein choles‐ terol, LDL‐c; high‐density lipoprotein cholesterol, HDL‐c; triglycerides). Left side: all ge‐ netic variants, right side: genetic variants having primary association with the target expo‐ sure. Solid line represents IVW estimate, dashed line represents weighted median estimate, and dotted line represents MR‐Egger estimate. [file GEPI-40-304-s003.pdf]

Figure legends

Figure 1: Illustrative diagram representing the hypothesized relationships between genetic variant G_j, exposure X, disease Y and confounders U when G_j is a valid instrumental variable (IV). Crosses indicate violations of assumptions IV2 and IV3 that potentially lead to invalid inferences from conventional methods.

Figure 2: Fictional example of a Mendelian randomization analysis with 10 genetic variants – 6 valid instrumental variables (hollow circles) and 4 invalid instrumental variables (solid circles) for finite sample size (left) and infinite sample size (right) showing inverse variance weighted (IVW, solid line) and simple median (dashed line) estimates compared with the true causal effect (dotted line).

Figure 3: Empirical distribution functions of ordered ratio instrumental variable estimates ($\hat{\beta}_j$) used for calculation of the simple median estimate (black) and two weighted median estimates (shown in red and blue) using the weights given in Table I.

Figure 4: Scatter plots of genetic associations with the outcome (coronary artery disease risk, CAD) against genetic associations with the exposure (low-density lipoprotein cholesterol, LDL-c; high-density lipoprotein cholesterol, HDL-c; triglycerides). Left side: all genetic variants, right side: genetic variants having primary association with the target exposure. Solid line represents IVW estimate, dashed line represents weighted median estimate, and dotted line represents MR-Egger estimate.
