# Supplementary material for: Consistent Estimation in Mendelian Randomization with Some Invalid Instruments Using a Weighted Median Estimator
Source: Genet Epidemiol. 2016 Apr 7;40(4):304–14. doi: 10.1002/gepi.21965 (PMC4849733; doi:10.1002/gepi.21965)
Supplement: Supplementary file 4 — Supporting Information S1 [file GEPI-40-304-s004.pdf]

Annotated

Consistent estimation in Mendelian randomization with some invalid instruments using a weighted median estimator

Jack Bowden
Integrative Epidemiology Unit, University of Bristol

George Davey Smith
Integrative Epidemiology Unit, University of Bristol

Philip C. Haycock
Integrative Epidemiology Unit, University of Bristol

Stephen Burgess*
Department of Public Health and Primary Care, University of Cambridge

Running title: Mendelian randomization with some invalid instruments [48 chars].

*Corresponding author. Address: Department of Public Health & Primary Care, Strangeways Research Laboratory, 2 Worts Causeway, Cambridge, CB1 8RN, UK. Telephone: +44 1223 740002. Correspondence to: sb452@medschl.cam.ac.uk.

Abstract

Developments in genome-wide association studies and the increasing availability of summary genetic association data have made application of Mendelian randomization relatively straightforward. However, obtaining reliable results from a Mendelian randomization investigation remains problematic, as the conventional inverse-variance weighted method only gives consistent estimates if all of the genetic variants in the analysis are valid instrumental variables. We present a novel weighted median estimator for combining data on multiple genetic variants into a single causal estimate. This estimator is consistent even when up to 50% of the information comes from invalid instrumental variables. In a simulation analysis, it is shown to have better finite-sample Type 1 error rates than the inverse-variance weighted method, and is complementary to the recently proposed MR-Egger (Mendelian randomization-Egger) regression method. In analyses of the causal effects of low-density lipoprotein cholesterol and high-density lipoprotein cholesterol on coronary artery disease risk, the inverse-variance weighted method suggests a causal effect of both lipid fractions, whereas the weighted median and MR-Egger regression methods suggest a null effect of high-density lipoprotein cholesterol that corresponds with the experimental evidence. Both median-based and MR-Egger regression methods should be considered as sensitivity analyses for Mendelian randomization investigations with multiple genetic variants.

Key words: Mendelian randomization, instrumental variables, robust statistics, Egger regression, pleiotropy.

Introduction

Over the past decade, Mendelian randomization has become an established tool for probing questions of causality when characterizing the aetiology of disease [Davey Smith and Ebrahim, 2003; Burgess and Thompson, 2015]. The requirement for such approaches stems from a fundamental limitation of observational data, namely that causation cannot automatically be inferred from an association between an exposure and a disease. The association could be due to unobserved confounding between the exposure and the outcome, or reverse causation (the outcome affects the exposure) [Davey Smith and Ebrahim, 2004]. These limitations are generally of no consequence when the aim is merely to predict the likelihood of future outcomes. However, if an exposure has a non-causal association with an outcome, then public health or pharmaceutical interventions targeted at the exposure will realize no material benefit and represent a waste of resources.

The basic premise of Mendelian randomization relies on genetic variants that explain variation in the exposure, but do not affect the disease outcome except possibly through the exposure. Such genetic variants are known as instrumental variables (IVs) [Greenland, 2000]. Subgroups of individuals with differing numbers of alleles of a genetic IV can be thought of as having been randomized to receive a different mean level of the exposure during their life course [Davey Smith and Ebrahim, 2005; Nitsch et al., 2006]. If the randomization is indeed uncontaminated (in the sense that a person's genetic subgroup is independent of all factors, except the exposure and any causal consequence of the exposure), then differences in the outcome between genetic subgroups can be causally attributed to the exposure [Didelez and Sheehan, 2007]. The following three assumptions are necessary for a genetic variant to be a valid IV [Martens et al., 2006]:

- IV1: the variant is predictive of the exposure;
- IV2: the variant is independent of any confounding factors of the exposure–outcome association;
- IV3: the variant is conditionally independent of the outcome given the exposure and the confounding factors.

The IV assumptions are illustrated in Figure 1. IV1 is the only assumption that can be fully empirically tested, since IV2 and IV3 depend on all possible confounders of the exposure–outcome association, both measured and unmeasured. Any statistical method for obtaining causal inferences must by necessity make an untestable assumption. The validity of a causal

conclusion from a Mendelian randomization analysis depends on the plausibility of these assumptions.

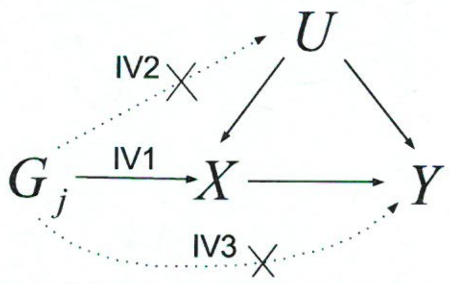

Figure 1: Illustrative diagram representing the hypothesized relationships between genetic variant G_j, exposure X, disease Y and confounders U when G_j is a valid instrumental variable (IV). Crosses indicate violations of assumptions IV2 and IV3 that potentially lead to invalid inferences from conventional methods.

Early implementations of the Mendelian randomization approach were largely constrained by limitations of power as investigations were undertaken in small sample sizes, and used only a handful of genetic variants (each explaining a small proportion of the variance in the exposure). However, a revolution in the field is under way led by the identification of increasing numbers of genetic variants robustly associated with particular traits, and the public release by many large consortia of summary association estimates for hundreds of thousands of genetic variants with exposures and disease outcomes [Burgess et al., 2015b], such as the Global Lipids Genetics Consortium (GLGC) for lipid fractions [Global Lipids Genetics Consortium, 2013] and the CARDIoGRAM consortium for coronary artery disease risk [CARDIoGRAMplusC4D Consortium, 2013]. The availability of such summary data has facilitated powerful Mendelian randomization investigations to be conducted in a two-sample framework by providing genetic associations precisely estimated in large sample sizes [Burgess et al., 2013].

The inclusion of multiple variants in a Mendelian randomization analysis typically leads to increased statistical power [Freeman et al., 2013], but presents new challenges [Glymour et al., 2012]. Firstly, if there is substantial overlap in the datasets from which the association estimates with the exposure and with the outcome were obtained, then the resulting analysis

suffers from bias and inflated type 1 error rates when the included variants are 'weak' (that is, they do not explain a substantial proportion of variation in the exposure in the dataset under analysis) [Burgess et al., 2011; Pierce and Burgess, 2013]. Secondly, it may not be the case that all included genetic variants are valid IVs.

In this paper we propose a new method for Mendelian randomization using summary data that offers protection against invalid instruments: the weighted median estimator. This approach can provide a consistent estimate of the causal effect even when up to 50% of the information contributing to the analysis comes from genetic variants that are invalid IVs. We explore the statistical properties of the weighted median estimator in a realistic simulation study, and compare with an alternative summary data analysis method also robust to some violations of the IV assumptions, MR-Egger regression [Bowden et al., 2015]. We explain how the two approaches differ in their assumptions, and when they each work well or fail. We provide an illustrative estimate of two-sample Mendelian randomization using summary data on the associations of 185 genetic variants with high-density lipoprotein cholesterol, low-density lipoprotein cholesterol, and triglycerides from the GLGC, and with coronary artery disease risk from the CARDIoGRAM consortium. We conclude with a discussion of the issues raised and the potential for future research.

Methods

Consider data from a Mendelian randomization study on J genetic variants $G_1, \dots, G_J$, a continuous exposure X and a continuous outcome Y. All confounding variables are subsumed into a single variable U. We initially assume that all genetic variants are valid IVs, and further assume that all the relationships between variables in Figure 1 are linear without heterogeneity or effect modification:

$$X|G_j = \gamma_0 + \gamma_j G_j + \epsilon_{Xj}$$
$$Y|G_j = \Gamma_0 + \Gamma_j G_j + \epsilon_{Yj}.$$

Assumption IV1 tells us that all variants are associated with the exposure, so $\gamma_j \neq 0$ for all j. Assumptions IV2 and IV3 tell us that the genetic associations with the outcome Γ_j are equal to the genetic associations with the exposure γ_j multiplied by the causal effect of the exposure on the outcome β: so $\Gamma_j = \beta\gamma_j$. The error terms ϵ_{Xj} and ϵ_{Yj} are assumed to be normally distributed and contain contributions from the confounder U and all genetic variants except G_j. In a one-sample setting, the exposure and outcome data are collected on

the same individuals, in which case ϵ_{Xj} and ϵ_{Yj} are correlated. If exposure and outcome data are collected on different sets of individuals (known as two-sample Mendelian randomization [Pierce and Burgess, 2013]), then these error terms are independent. We assume throughout this manuscript that all genetic variants are uncorrelated (that is, not in linkage disequilibrium), so that the information provided by each genetic variant is independent. Extensions to allow for correlated variants in a straightforward application of Mendelian randomization have been developed [Burgess et al., 2013], but the situation of uncorrelated variants is usual in applied practice.

Inverse-variance weighted method

The causal effect of the exposure on the outcome can be estimated using the jth variant as the ratio of the gene–outcome association and the gene–exposure association estimates [Lawlor et al., 2008]:

$$\hat{\beta}_j = \frac{\hat{\Gamma}_j}{\hat{\gamma}_j}.$$

If the IV assumptions are satisfied for genetic variant j, then $\Gamma_j = \beta\gamma_j$ and the ratio estimate is consistent asymptotically. Furthermore, if the genetic variants are uncorrelated (not in linkage disequilibrium) then the ratio estimates from each genetic variant can be combined into an overall estimate using a formula from the meta-analysis literature [Johnson, 2013]:

$$\hat{\beta}_{IVW} = \frac{\sum_j \hat{\gamma}_j^2 \sigma_{Yj}^{-2} \hat{\beta}_j}{\sum_j \hat{\gamma}_j^2 \sigma_{Yj}^{-2}}$$

where σ_{Yj} is the standard error of the gene–outcome association estimate for variant j. This is referred to as the inverse variance weighted (IVW) estimator [Burgess et al., 2013]. Provided that the genetic variants are uncorrelated, the IVW estimate is asymptotically equal to the two-stage least squares estimate commonly used with individual-level data. If all genetic variants satisfy the IV assumptions, then the IVW estimate is a consistent estimate of the causal effect (that is, it converges to the true value as the sample size increases), as it is a weighted mean of the individual ratio estimates.

Simple median estimator

The IVW estimate is an efficient analysis method when all genetic variants are valid IVs. Unfortunately, it will be biased even if only one genetic variant is invalid. For this reason, the IVW estimate can be said to have a 0% breakdown level. However, an estimator exists that

enjoys a 50% breakdown level; that is, it provides a consistent estimate of the causal effect when up to (but not including) 50% of genetic variants are invalid. This simple estimator is the median ratio estimate [Han, 2008]. Specifically, let $\hat{\beta}_j$ denote the jth ordered ratio estimate (arranged from smallest to largest). If the total number of genetic variants is odd ($J = 2k + 1$), the simple median estimator is the middle ratio estimate $\hat{\beta}_{k+1}$. If it is even ($J = 2k$), the median is interpolated between the two middle estimates $\frac{1}{2}(\hat{\beta}_k + \hat{\beta}_{k+1})$. In terms of notation, we assume in this manuscript that genetic variants are ordered according to their ratio estimates.

A4

In order to understand why the median estimator achieves a 50% breakdown level, we consider a fictional analysis using ten genetic variants, six of which are valid IVs and four of which are invalid. Figure 2 (left) shows a scatter plot of 10 gene–exposure ($\hat{\gamma}_j$) and gene–outcome ($\hat{\Gamma}_j$) association estimates for an Mendelian randomization study with a finite sample size [Kang et al., 2015]. The ratio estimate for each genetic variant is the gradient of the line connecting the relevant datapoint for that variant to the origin. The true causal effect is shown by the dotted black line, the median estimate by the dashed line, and the IVW estimate by the solid line. Estimates from valid IVs are shown by hollow circles, estimates from invalid IVs are shown by solid circles. Although the valid IVs follow the true slope, the IVW estimate is pulled away from the true value by the invalid instruments, which yield biased estimates of the causal effect. Figure 2 (right) shows the same scatter plot for an infinite sample size. Now the six valid instruments lie perfectly on the true line, and all yield the same true causal estimate. The median ratio estimate (in this case, an average of the 5th and 6th ratio estimates) is the true causal effect. In contrast, the IVW estimate remains biased even with infinite data, as the ratio estimate from each genetic variant always contributes towards the overall IVW estimate.

Weighted median estimator

The simple median estimator is inefficient, especially when the precision of the individual estimates varies considerably. In order to account for this, a weighted median can be defined as follows. Let w_j be the weight given to the jth ordered ratio estimate, and let $s_j = \sum_{k=1}^{j} w_k$ be the sum of weights up to and including the weight of the jth ordered ratio estimate. Weights are standardized, so that the sum of the weights s_J is 1. The weighted median estimator is the median of a distribution having estimate $\hat{\beta}_j$ as its $p_j = 100(s_j - \frac{w_j}{2})$th percentile. For all other percentile values, we extrapolate linearly between the neighboring ratio estimates. The contribution of the jth genetic variant to the empirical distribution is proportional to its weight w_j. The simple median estimator can be thought of as a weighted

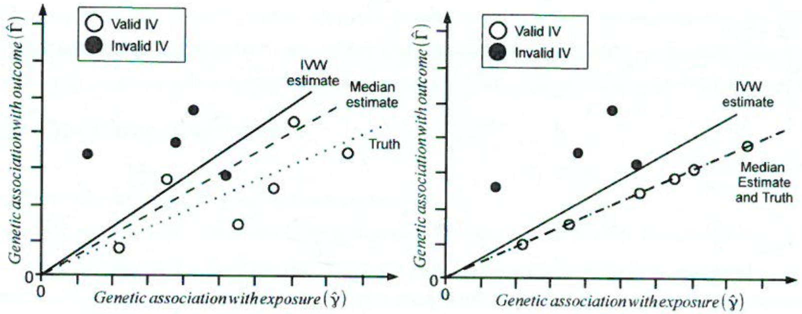

A4

Figure 2: Fictional example of a Mendelian randomization analysis with 10 genetic variants – 6 valid instrumental variables (hollow circles) and 4 invalid instrumental variables (solid circles) for finite sample size (left) and infinite sample size (right) showing inverse variance weighted (IVW, solid line) and simple median (dashed line) estimates compared with the true causal effect (dotted line). The ratio estimate for each genetic variant is the gradient of the line connecting the relevant datapoint for that variant to the origin; the simple median estimate is the median of these ratio estimates.

median estimator with equal weights. While the simple median provides a consistent estimate of causal effect if at least 50% of IVs are valid, the weighted median will provide a consistent estimate if at least 50% of the weight comes from valid IVs. We assume that no single IV contributes more than 50% of the weight, otherwise the 50% validity assumption is equivalent to assuming that this IV is valid (in which case, an analysis should simply be based on this one IV). Some technical remarks on weighted medians are given in Web Appendix 1.

As an illustration, two sets of weights are given in Table I, and percentiles are calculated for each set of weights as well as for the simple median (equal weights). As the first set of weights are symmetric, the weighted median in this case equals the simple median. However, less weight is given to outlying estimates, and the empirical distribution function (Figure 3, red line) is close to the median value across a wider range of the distribution. Confidence intervals for the weighted median, which can be obtained by a parametric bootstrap method, should therefore be narrower. In the second set of weights, smaller estimates happen to receive more weight (Figure 3, blue line). The weighted median estimate will be interpolated between ratio estimates $\hat{\beta}_3$ and $\hat{\beta}_4$, but will be closer to $\hat{\beta}_4$ as the percentile p_4 is closest to 50%. The exact weighted median estimate in this case will be:

$$\hat{\beta}_{WM} = \hat{\beta}_3 + (\hat{\beta}_4 - \hat{\beta}_3) \times \frac{50 - 27.78}{52.78 - 27.78}$$

A5

The weighted median can also be thought of as the simple median from a set of values (a pseudo-population) in which the ratio estimate $\hat{\beta}_1$ for variant 1 appears $100 \times w_1$ times, ratio estimate $\hat{\beta}_2$ for variant 2 appears $100 \times w_2$ times, and so on. R code to calculate weighted median estimates, confidence intervals, standard errors and p-values is provided in Web Appendix 2.

[Table I should appear about here]

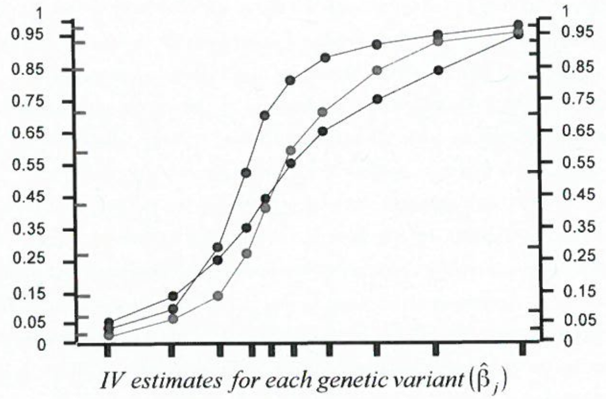

Figure 3: Empirical distribution functions of ordered ratio instrumental variable estimates ($\hat{\beta}_j$) used for calculation of the simple median estimate (black) and two weighted median estimates (shown in red and blue) using the weights given in Table I.

Analogously to the IVW method, we suggest using the inverse of the variance of the ratio estimates as weights:

$$w'_j = \frac{\hat{\gamma}_j^2}{\sigma_{Y_j}^2}$$

A3

These weights are derived from the delta method for the variance of the ratio of two random variables, and represent the reciprocal of the variance of the ratio estimates (the inverse-variance weights) [Thomas et al., 2007]. Standardized weights are $w_j = \frac{w'_j}{\sum_j w'_j}$. The unstandardized weights are identical to those used in the IVW estimator. Only the first order term from the delta expansion is used here; further terms could be considered, although we found that they did not affect estimates or standard errors from the weighted median method substantially.

Penalized weighted median estimator

In Figure 2 (left side), although the invalid IVs do not contribute directly to the median estimate, they do influence it. The simple median estimate in this case is an average of the 5th and 6th ratio estimates. If the invalid IVs were not present, the median estimate would be the average of the 3rd and 4th ratio estimates. The presence of invalid instruments does not affect the median estimate asymptotically, but in this example it will bias the estimate in finite samples, as the 5th and 6th ratio estimates will always be larger than the 3rd and 4th ratio estimates. This is likely to be a problem when the estimates from invalid IVs are not balanced about the true causal effect (as in this example, all 4 invalid estimates are greater than the true causal effect).

One way of minimizing this problem is downweighting the contribution to the analysis of genetic variants with heterogeneous ratio estimates. Heterogeneity between estimates can be quantified by Cochran's Q statistic:

$$Q = \sum_j Q_j = \sum_j w'_j(\hat{\beta}_j - \hat{\beta})^2$$

where we take $\hat{\beta}$ to be the IVW estimate [Greco et al., 2015]. The Q statistic has a chi-squared distribution on $J - 1$ degrees of freedom under the null hypothesis that all genetic variants are valid IVs and the same causal effect is identified by all variants. Under this null hypothesis, the components of the Q statistic corresponding to the individual genetic variants (Q_j) approximately have chi-squared distributions with 1 degree of freedom. So as not to distort the weightings of the majority of variants, we propose penalization using the one-sided upper p-value (denoted q_j) on a chi-squared-1 distribution corresponding to Q_j, by multiplying the weight by the p-value multiplied by 20 (or by 1 if the p-value is greater than 0.05). The (unstandardized) penalized weights (w^*_j) are therefore:

$$w^*_j = w'_j \times \min(1, 20q_j)$$

This means that most variants will be unaffected by the penalization, but outlying variants will be severely downweighted.

MR-Egger regression

B2

An alternative robust method for Mendelian randomization with summary data has been recently proposed by Bowden et al. [Bowden et al., 2015], referred to as 'MR-Egger regression'. This approach was motivated from a method in the meta-analysis literature for the

B2

assessment of small-study bias (often called "publication bias") [Egger et al., 1997]. This performs a weighted linear regression of the gene–outcome coefficients $\hat{\Gamma}_j$ on the gene–exposure coefficients $\hat{\gamma}_j$:

$$\hat{\Gamma}_j = \beta_{0E} + \beta_E \hat{\gamma}_j$$

in which all the $\hat{\gamma}_j$ associations are orientated to be positive (the orientation of the $\hat{\Gamma}_j$ associations should be altered if necessary to match the orientation of the $\hat{\gamma}_j$ parameters), and the weights in the regression are the inverse-variances of the gene–outcome associations (σ_{Yj}^{-2}). Re-orientation of the variants is performed as the orientation of genetic variants is arbitrary (that is, estimates can be presented with reference to either the major or minor allele), and different orientations of genetic variants change the estimate of the intercept, as well as the sign and magnitude of the pleiotropic effect of the genetic variant. If there is no intercept term in the regression model, then the MR-Egger slope estimate $\hat{\beta}_E$ will equal the IVW estimate [Burgess et al., 2015a].

A6

The value of the intercept term $\hat{\beta}_{0E}$ can be interpreted as an estimate of the average pleiotropic effect across the genetic variants [Bowden et al., 2015]. The pleiotropic effect is the effect of the genetic variant on the outcome that is not mediated via the exposure. An intercept term that differs from zero is indicative of overall directional pleiotropy: that is, pleiotropic effects do not cancel out and the IVW estimate is biased.

MR-Egger regression additionally provides an estimate for the true causal effect $\hat{\beta}_E$ that is consistent even if all genetic variants are invalid due to violation of IV3, but under a weaker assumption known as the InSIDE (instrument strength independent of direct effect) assumption. If the association of the jth genetic variant with the outcome $\Gamma_j = \beta\gamma_j + \alpha_j$, where α_j is the pleiotropic (direct) effect of the variant, then the InSIDE assumption states that the pleiotropic effects α_j must be distributed independently of the instrument strength parameters γ_j [Kolesár et al., 2014]. (Formally, the consistency property holds both as the sample size and the number of instruments increases. For a fixed number of instruments, consistency only holds asymptotically if the correlation between the α_j and γ_j parameters is zero.) The InSIDE assumption is likely to be satisfied if pleiotropic effects on the outcome are direct (that is, not via a confounder). There is some empirical evidence supporting the proposition that genetic effects on separate exposures are independent [Pickrell, 2015]. However, if the pleiotropic effects of genetic variants are all via a single confounder, then they will be correlated with instrument strength, and the InSIDE assumption will be violated.

Simulation study

In order to investigate the performance of the weighted median method in realistic settings, as well as to determine in what scenarios it performs well or badly in comparison with the IVW and MR-Egger regression methods, we perform a simulation study. We assume there are 25 genetic variants that are candidate IVs, and consider three scenarios:

1. balanced pleiotropy, InSIDE assumption satisfied – pleiotropic effects are equally likely to be positive as negative, these effects are uncorrelated with the instrument strength;
2. directional pleiotropy, InSIDE assumption satisfied – only positive pleiotropic effects are simulated, these effects are uncorrelated with the instrument strength;
3. directional pleiotropy, InSIDE assumption not satisfied – pleiotropic effects are via a confounder, these effects on the outcome are therefore positive and are correlated with the instrument strength.

The status of a genetic variant as an invalid IV is determined by a random draw for each variant. The probability of being an invalid variant is taken as 0.1, 0.2, and 0.3. We consider cases with 10 000 and 20 000 participants.

We generated 10 000 simulated datasets for each scenario in a two-sample setting with two values of the causal effect ($\beta = 0$, null causal effect; $\beta = 0.1$, positive causal effect); the simulations are repeated in Web Appendix 3 in a one-sample setting. Only the summary data on the genetic associations with the exposure and with the outcome (and their standard errors) are used as data inputs in the analysis methods (individual-level data are not used). Details of the data-generating model and the parameters used in the simulation study are given in Web Appendix 3.

Simulation results

A7

The simulation results in the two-sample setting are given in Table II (null causal effect) and Table III (positive causal effect). In Scenario 1 (balanced pleiotropy), the methods all give close to unbiased causal estimates, and have reasonable Type 1 error rates (power under the null is equal to Type 1 error). However, the power of the estimates with a positive causal effect differs substantially. The weighted median methods have lower mean standard errors than the IVW method, and generally have greater power with a positive causal effect (although not uniformly so), particularly as the proportion of invalid IVs increases. This is because invalid IVs do not influence the median estimates directly. Although Type 1 error

rates from MR-Egger regression are at nominal levels, estimates from the MR-Egger method are considerably less precise (mean standard errors are around 3 times larger than IVW standard errors), and power to detect a causal effect is considerably reduced. Precision in the MR-Egger method depends on the genetic variants having different associations with the exposure; if all genetic variants had the same magnitude of association with the exposure, then the MR-Egger regression estimate would not be identified.

In Scenario 2 (directional pleiotropy, InSIDE assumption satisfied), estimates from the IVW method are biased with inflated Type 1 error rates. Estimates from the weighted median methods are less biased, although there is a consistent bias in the direction of the pleiotropic variants. Nominal Type 1 error rates are maintained when only 10% of genetic variants are invalid IVs, although Type 1 error rates are above the expected 5% rate when 20% or more genetic variants are invalid IVs. However, even though they are inflated, Type 1 error rates are far lower for the median-based methods than those from the IVW method. The penalized weighted median estimates are less biased when a few genetic variants are invalid, but when 30% of genetic variants are invalid, it seems that the invalid IVs are often retained in the analysis (as their estimates are homogeneous with each other) and valid IVs are excluded. Estimates from MR-Egger regression are close to unbiased, and Type 1 error rates are at nominal levels, suggesting its utility as a sensitivity analysis method when the InSIDE assumption is satisfied. However, the wide confidence intervals and low power with a true causal effect (close to 5%) mean that MR-Egger regression is not likely to be a discerning sensitivity analysis, but rather giving conservative findings as to whether a causal effect is present or not. Although power to detect a causal effect is greater in the IVW method, this is achieved at the cost of the Type 1 error rate; comparisons of power that do not make reference to the Type 1 error rate are meaningless.

In Scenario 3 (directional pleiotropy, InSIDE assumption not satisfied), all methods suffer from bias and inflated Type 1 error rates. Most concerning are results from the MR-Egger method, which are more biased than those from the IVW method and have similar Type 1 error rates. The weighted median methods, and in particular the penalized weighted median method, give lower Type 1 error rates, suggesting their potential use as a sensitivity analysis method when InSIDE is not satisfied, as well as when it is satisfied.

In Web Tables A1 and A2, results are presented in a one-sample setting. Each of the methods suffers from weak instrument bias in this setting, and Type 1 error rates are inflated. Estimates from MR-Egger regression are substantially more biased than those from other methods. However, the median-based methods remain a reasonable sensitivity analysis, as Type 1 error rates are similar to those of the IVW method in Scenario 1, and substantially

lower in Scenarios 2 and 3. In Web Table A3, results are presented in a two-sample setting for the simple median estimator, and a weighted median estimator using inverse-standard error weights rather than the inverse-variance weights used above. Simple median estimates are slightly less precise than those from the weighted median methods, and have similar bias and Type 1 error properties in Scenarios 1 and 2, but much improved Type 1 error rates in Scenario 3. This is because the invalid variants in this scenario are stronger than the valid variants, and so receive more weight in the weighted analyses. It is unclear that this would occur in applied practice, although it suggests that the simple median method is a worthwhile additional sensitivity analysis. The inverse-standard error weighted median estimator has improved Type 1 error properties compared with the inverse-variance weighted median estimator particularly in Scenario 3 (although not uniformly), indicating the potential to use different choices of weights in median-based methods to provide further sensitivity analyses.

[Tables II and III should appear around here]

Example: lipid concentrations and coronary artery disease risk

Low-density lipoprotein cholesterol (LDL-c) is observationally positively associated with coronary artery disease (CAD) risk [Di Angelantonio et al., 2009]. Evidence from randomized trials of pharmaceutical interventions to lower LDL-c concentrations (such as statins [Pedersen et al., 1994; Cheung et al., 2004]), and from Mendelian randomization [Linsel-Nitschke et al., 2008; Do et al., 2013] suggests that this association is reflective of a causal effect of LDL-c on CAD risk. On the contrary, although high-density lipoprotein cholesterol (HDL-c) is observationally inversely associated with CAD risk, interventions to raise HDL-c concentrations [Schwartz et al., 2012] and focused Mendelian randomization investigations [Voight et al., 2012] suggest that there is not even a moderate causal effect of HDL-c on CAD risk. However, a more liberal Mendelian randomization analysis including genetic variants associated with other lipid fractions (in particular, triglycerides) did suggest a causal effect of HDL-c on CAD risk [Holmes et al., 2015]. As a proof of concept example, we use summary data from the GLGC on genetic associations with lipid fractions, and from CARDIoGRAM on associations with CAD risk, and perform the analysis methods discussed in this paper to see whether the causal effect of LDL-c is preserved by the weighted median and MR-Egger regression methods, as well as whether the spurious causal effect of HDL-c is contradicted by the robust methods. We use data provided by Do et al. [Do et al., 2013] on 185 genetic

variants for this analysis. The genetic associations with the lipid fractions are in standard deviation units, and with the outcome are log odds ratios, so the causal effects represent log odds ratios per 1 standard deviation increase in the lipid fraction. Further details of the analysis are provided in Web Appendix 4.

Taking each of LDL-c, HDL-c, and additionally triglycerides as the exposure variable in turn, we consider two analysis strategies. First, we take all genetic variants associated with the exposure at a genome-wide level of significance (taken as $p < 10^{-8}$). Second, we restrict to genetic variants for which the p-value for association with the target exposure (say, HDL-c) is less than the p-values for association with the non-target exposures (say, LDL-c and triglycerides). We perform each of the methods presented in the manuscript (IVW, simple median, weighted median, penalized weighted median, MR-Egger regression). The data are presented as scatter plots in Figure 4 and Web Figure A1, and as funnel plots in Web Figure A2.

[Table IV should appear about here]

Results are given in Table IV. The causal effects of LDL-c and triglycerides are robustly detected by all the analysis methods. In contrast for HDL-c, both MR-Egger regression and the weighted median methods suggest that the 'causal effect' of HDL-c on CAD risk detected by the IVW method is suspect. With the exception of the simple median estimates, all other robust analysis methods give estimates compatible with the null. The test for directional pleiotropy in the MR-Egger regression method suggested pleiotropy for HDL-c, but not for LDL-c or triglycerides. Although similar results for HDL-c can be obtained by filtering out genetic variants that are suspected to have pleiotropic effects, by omitting genetic variants there is a loss of power (for example, in Holmes et al. a liberal genetic risk score explained 3.8% of the variance in HDL-c, whereas a conservative score omitting potentially pleiotropic variants explained only 0.3%).

By way of comparison between the robust methods, similar results were seen in this example as in the simulation study. The median-based methods were consistently and substantially more precise than the MR-Egger regression method, with standard errors reduced by around 30-50%. The precision of the weighted median methods, in particular the penalized weighted median method, was not much worse than that of the IVW method, and in some cases was slightly better. The simple median method was not as impressive, suggesting a causal effect of HDL-c on CAD risk, and giving less precise estimates than those from the weighted median methods. The MR-Egger regression method performed well despite doubts about the InSIDE assumption in the case of HDL-c; many variants associated with HDL-c

A4 (lines added)

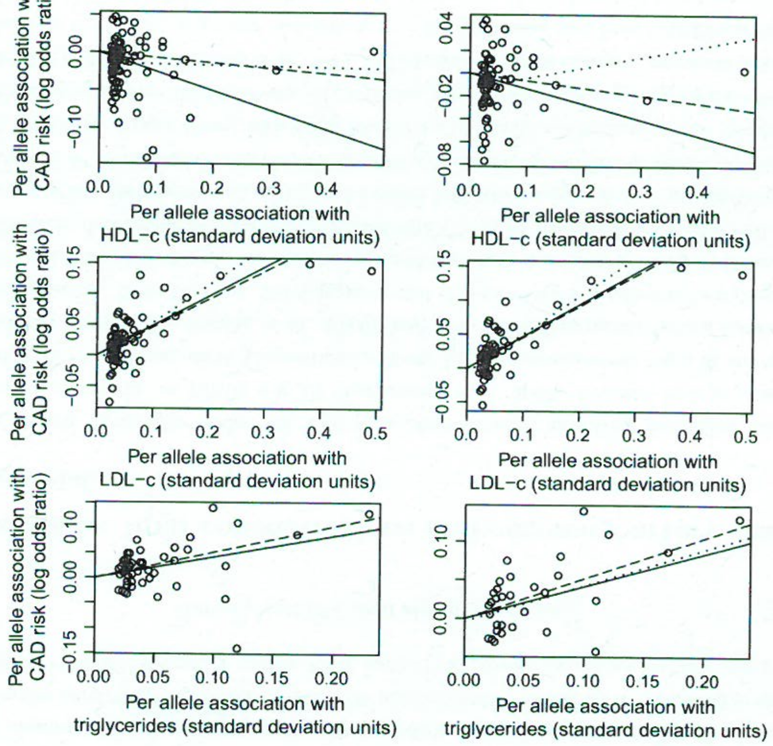

A4 Figure 4: Scatter plots of genetic associations with the outcome (coronary artery disease risk, CAD) against genetic associations with the exposure (low-density lipoprotein cholesterol, LDL-c; high-density lipoprotein cholesterol, HDL-c; triglycerides). Left side: all genetic variants, right side: genetic variants having primary association with the target exposure. Solid line represents IVW estimate, dashed line represents weighted median estimate, and dotted line represents MR-Egger estimate.

are also associated with LDL-c and triglycerides, and these associations are approximately proportional (see Web Figure A1), hence pleiotropic effects on CAD risk may operate via LDL-c and triglycerides. This may be the reason why the MR-Egger estimate changed sign in the analysis using variants having primary association with HDL-c.

Discussion

In this paper, we have introduced a weighted median method for the estimation of a causal effect using multiple instrumental variables. Unlike other methods commonly used in Mendelian randomization, this method can give consistent estimates when some of the genetic variants in the analysis are not valid IVs. We have shown how the method performs in a simulation study and in an applied example.

A summary of the median-based and other methods considered in this paper is given in Table V. Of particular interest is the comparison between the weighted median approach and MR-Egger regression. MR-Egger regression can give consistent estimates when 100% of genetic variants are invalid IVs, whereas the weighted median method requires 50% of the weight to come from valid IVs. However, the weighted median approach allows the IV assumptions to be violated in a more general way for the invalid IVs, whereas MR-Egger regression replaces one set of untestable assumptions (IV2 and IV3) with a weaker, but still untestable assumption (the InSIDE assumption). Additionally, weighted median estimates were substantially more precise than those from MR-Egger regression in the simulation study and applied example. MR-Egger regression estimates are likely to be particularly imprecise if all genetic variants have similar magnitudes of association with the exposure.

B3 B5

Although the only mechanism for generating invalid IVs considered in this paper was pleiotropy, median-based methods are agnostic to the mechanism by which the invalid IVs violate the IV assumptions. Consistent estimates would be guaranteed if some genetic variants were invalid IVs due to other mechanisms, such as linkage disequilibrium, population stratification, or differential survival [Bochud et al., 2008]. One potential source of bias that may not be resolved by the proposed robust methods is selection bias [Hernán et al., 2004]. This could arise from differential selection of individuals in the datasets from which the genetic associations are obtained, or else the selection of genetic variants based on their strength in the dataset under analysis, or if genetic variants were discovered as associated with the exposure in the dataset under analysis. Selection bias may affect all genetic variants in a particular analysis, and so is unlikely to be addressed by the use of the median-based methods proposed in this paper.

B1

We clarify that the objective of this paper is not to provide guidance on how to choose genetic variants for a Mendelian randomization analysis. This is an important question, but not one that it is possible to answer in a general way, in that it requires biological as well as statistical considerations of the specific analysis question in each case. The robust analysis methods presented in this paper are able to weaken the assumptions necessary for the consistent estimation of a causal effect. However, they do not eliminate the need to assess the validity of the instrumental variable assumptions. This is particularly true for the median-based approaches, as the assumption that 50% of the information in the analysis comes from valid instrumental variables is still required. As a corollary, these robust methods should not be used to promote analysis approaches based on the whole genome for causal inference without further justification.

[Table V should appear about here]

One assumption that we have made in this paper is that of no causal effect heterogeneity. This means that the same change in the outcome is expected no matter how the exposure is intervened on, and so the same causal effect is identified by different genetic variants that are valid IVs. In practice, it may be that genetic variants influence the exposure via different biological mechanisms. However, empirical evidence for the causal effect of LDL-c on CAD risk provides no evidence of causal effect heterogeneity despite genetic variants having different mechanisms and substantially different magnitudes of association with LDL-c [Davey Smith and Hemani, 2014].

Other approaches for the estimation of causal effects with some invalid instrumental variables have been proposed. Kang et al. have proposed a method based on penalized regression for detecting and accounting for invalid instruments that provides a consistent estimate of causal effect if at least 50% of the genetic variants are valid [Kang et al., 2015]. Han has proposed a similar penalized estimator within the generalized method of moments framework, again with a 50% breakdown level [Han, 2008]. Kolesár et al. have proposed a method within the framework of k-class estimators with a 100% breakdown level under the InSIDE assumption [Kolesár et al., 2014]. The first two methods are similar to the median-based approaches proposed here, and the final method is similar to MR-Egger regression. An alternative approach was proposed by Windmeijer et al. based on Hansen's overidentification test, a test of the homogeneity of the ratio estimates from different candidate instrumental variables [Windmeijer et al., 2015]. The basic idea of this approach is to report a causal estimate based on genetic variants whose ratio estimates are mutually similar. A related approach based on step-wise selection using a heterogeneity test statistic was proposed by

A9

Johnson [Johnson, 2013]. While the exclusion of certain variants to consider their potential impact on the overall estimate may be a reasonable sensitivity analysis, a potential danger of such an approach is that of *post hoc* or data-driven analysis, in which genetic variants are cherry-picked for inclusion in the analysis model, and dissenting variants are filtered out.

We have chosen in this paper to focus on the two-sample situation using summary data as this is most relevant to Mendelian randomization; all the other methods have been developed for use with individual-level data in a one-sample setting, and hence are not considered in this manuscript. We look forward to further methodological developments in this area. One particular area that may be fruitful for such developments is bias-correction methods in the meta-analysis literature; two particular examples are the trim-and-fill method [Duval and Tweedie, 2000] and the use of pseudo-data [Bowden et al., 2006]. Equivalently, there may be application of the median-based methods proposed in this paper in the field of meta-analysis, to provide a more robust pooled estimate.

From a practical perspective, it is important to acknowledge the limitations of all methods for obtaining causal inferences. Our aim in presenting the median-based methods in this paper is not to recommend a single authoritative method for all Mendelian randomization analyses. Rather, examining the results from different methods that make different assumptions (IVW, simple median, weighted median, MR-Egger regression) provides a sensitivity analysis that either adds to or questions the robustness of a finding from a Mendelian randomization investigation. If, as in the case of the effect of LDL-c on CAD risk, a causal effect is reported across all methods, then a causal finding is far more plausible than if the methods give contradictory findings. Our advice in Mendelian randomization investigations using multiple genetic variants where the IV assumptions are in doubt for some or all genetic variants, would therefore be to perform and report results from a range of sensitivity analyses using robust methods, including the simple median, weighted median, and MR-Egger regression methods, in addition to the main analysis result.

Acknowledgements

The authors have no conflict of interest. Jack Bowden is supported by a Methodology Research Fellowship from the Medical Research Council (grant number MR/N501906/1). George Davey Smith is supported by the Medical Research Council (grant number MC_UU_12013/1-9). Philip C Haycock is supported by a Cancer Research UK Population Research Postdoctoral Fellowship. Stephen Burgess is supported by a fellowship from the Wellcome Trust (100114).

References

Bochud, M., Chiolero, A., Elston, R., and Paccaud, F. 2008. A cautionary note on the use of Mendelian randomization to infer causation in observational epidemiology. *International Journal of Epidemiology*, 37(2):414–416.

Bowden, J., Davey Smith, G., and Burgess, S. 2015. Mendelian randomization with invalid instruments: effect estimation and bias detection through Egger regression. *International Journal of Epidemiology*, 44(2):512–525.

Bowden, J., Thompson, J., and Burton, P. 2006. Using pseudo-data to correct for publication bias in meta-analysis. *Statistics in Medicine*, 25:3798–3813.

Burgess, S., Butterworth, A., and Thompson, S. 2013. Mendelian randomization analysis with multiple genetic variants using summarized data. *Genetic Epidemiology*, 37(7):658–665.

Burgess, S., Dudbridge, F., and Thompson, S. G. 2015a. Re: "Multivariable Mendelian randomization: the use of pleiotropic genetic variants to estimate causal effects". *American Journal of Epidemiology*, 181(4):290–291.

Burgess, S., Scott, R., Timpson, N., Davey Smith, G., Thompson, S., and EPIC-InterAct Consortium 2015b. Using published data in Mendelian randomization: a blueprint for efficient identification of causal risk factors. *European Journal of Epidemiology*, 30(7):543–552.

Burgess, S., Thompson, S., and CRP CHD Genetics Collaboration 2011. Avoiding bias from weak instruments in Mendelian randomization studies. *International Journal of Epidemiology*, 40(3):755–764.

Burgess, S. and Thompson, S. G. 2015. *Mendelian randomization: methods for using genetic variants in causal estimation.* Chapman & Hall.

CARDIoGRAMplusC4D Consortium 2013. Large-scale association analysis identifies new risk loci for coronary artery disease. *Nature Genetics*, 45(1):25–33.

Cheung, B., Lauder, I., Lau, C., and Kumana, C. 2004. Meta-analysis of large randomized controlled trials to evaluate the impact of statins on cardiovascular outcomes. *British Journal of Clinical Pharmacology*, 57(5):640–651.

Davey Smith, G. and Ebrahim, S. 2003. 'Mendelian randomization': can genetic epidemiology contribute to understanding environmental determinants of disease? *International Journal of Epidemiology*, 32(1):1–22.

Davey Smith, G. and Ebrahim, S. 2004. Mendelian randomization: prospects, potentials, and limitations. *International Journal of Epidemiology*, 33(1):30–42.

Davey Smith, G. and Ebrahim, S. 2005. What can Mendelian randomisation tell us about modifiable behavioural and environmental exposures? *British Medical Journal*, 330(7499):1076–1079.

Davey Smith, G. and Hemani, G. 2014. Mendelian randomization: genetic anchors for causal inference in epidemiological studies. *Human Molecular Genetics*, 23(R1):R89–98.

Di Angelantonio, E., Sarwar, N., Perry, P., et al. 2009. Major lipids, apolipoproteins, and risk of vascular disease. *Journal of the American Medical Association*, 302(18):1993–2000.

Didelez, V. and Sheehan, N. 2007. Mendelian randomization as an instrumental variable approach to causal inference. *Statistical Methods in Medical Research*, 16(4):309–330.

Do, R., Willer, C. J., Schmidt, E. M., et al. 2013. Common variants associated with plasma triglycerides and risk for coronary artery disease. *Nature Genetics*, 45:1345–1352.

Duval, S. and Tweedie, R. 2000. Trim and fill: A simple funnel-plot-based method of testing and adjusting for publication bias in meta-analysis. *Biometrics*, 56:455–463.

Egger, M., Smith, G. D., Schneider, M., and Minder, C. 1997. Bias in meta-analysis detected by a simple, graphical test. *British Medical Journal*, 315(7109):629–634.

Freeman, G., Cowling, B., and Schooling, M. 2013. Power and sample size calculations for Mendelian randomization studies. *International Journal of Epidemiology*, 42(4):1157–1163.

Global Lipids Genetics Consortium 2013. Discovery and refinement of loci associated with lipid levels. *Nature Genetics*, 45:1274–1283.

Glymour, M., Tchetgen Tchetgen, E., and Robins, J. 2012. Credible Mendelian randomization studies: approaches for evaluating the instrumental variable assumptions. *American Journal of Epidemiology*, 175(4):332–339.

Greco, M., Minelli, C., Sheehan, N. A., and Thompson, J. R. 2015. Detecting pleiotropy in Mendelian randomisation studies with summary data and a continuous outcome. *Statistics in Medicine*, 34(21):2926–2940.

Greenland, S. 2000. An introduction to instrumental variables for epidemiologists. *International Journal of Epidemiology*, 29(4):722–729.

Han, C. 2008. Detecting invalid instruments using L1-GMM. *Economics Letters*, 101:285–287.

Hernán, M., Hernández-Díaz, S., and Robins, J. 2004. A structural approach to selection bias. *Epidemiology*, 15(5):615–625.

Holmes, M. V., Asselbergs, F. W., Palmer, T. M., et al. 2015. Mendelian randomization of blood lipids for coronary heart disease. *European Heart Journal*, 36(9):539–550.

Johnson, T. 2013. Efficient calculation for multi-SNP genetic risk scores. Technical report, The Comprehensive R Archive Network. Available at http://cran.r-project.org/web/packages/gtx/vignettes/ashg2012.pdf [last accessed 2014/11/19].

Kang, H., Zhang, A., Cai, T., and Small, D. 2015. Instrumental variables estimation with some invalid instruments, and its application to Mendelian randomisation. *Journal of the American Statistical Association*, available online before print.

Kolesár, M., Chetty, R., Friedman, J., Glaeser, E., and Imbens, G. 2014. Identification and inference with many invalid instruments. *Journal of Business & Economic Statistics*, available online before print.

Lawlor, D., Harbord, R., Sterne, J., Timpson, N., and Davey Smith, G. 2008. Mendelian randomization: using genes as instruments for making causal inferences in epidemiology. *Statistics in Medicine*, 27(8):1133–1163.

Linsel-Nitschke, P., Götz, A., Erdmann, J., et al. 2008. Lifelong reduction of LDL-cholesterol related to a common variant in the LDL-receptor gene decreases the risk of coronary artery disease — a Mendelian randomisation study. *PLoS One*, 3(8):e2986.

Martens, E., Pestman, W., de Boer, A., Belitser, S., and Klungel, O. 2006. Instrumental variables: application and limitations. *Epidemiology*, 17(3):260–267.

Nitsch, D., Molokhia, M., Smeeth, L., DeStavola, B., Whittaker, J., and Leon, D. 2006. Limits to causal inference based on Mendelian randomization: a comparison with randomized controlled trials. *American Journal of Epidemiology*, 163(5):397–403.

Pedersen, T., Kjekshus, J., Berg, K., et al. 1994. Randomised trial of cholesterol lowering in 4444 patients with coronary heart disease: the Scandinavian Simvastatin Survival Study (4S). *The Lancet*, 344(8934):1383–1389.

Pickrell, J. 2015. Detection and interpretation of shared genetic influences on 40 human traits. Technical report, bioRxiv.

Pierce, B. and Burgess, S. 2013. Efficient design for Mendelian randomization studies: subsample and two-sample instrumental variable estimators. *American Journal of Epidemiology*, 178(7):1177–1184.

Schwartz, G. G., Olsson, A. G., Abt, M., et al. 2012. Effects of dalcetrapib in patients with a recent acute coronary syndrome. *New England Journal of Medicine*, 367(22):2089–2099.

Thomas, D., Lawlor, D., and Thompson, J. 2007. Re: Estimation of bias in nongenetic observational studies using "Mendelian triangulation" by Bautista et al. *Annals of Epidemiology*, 17(7):511–513.

Voight, B., Peloso, G., Orho-Melander, M., et al. 2012. Plasma HDL cholesterol and risk of myocardial infarction: a mendelian randomisation study. *The Lancet*, 380(9841):572–580.

Windmeijer, F., Farbmacher, H., Davies, N., Davey Smith, G., and White, I. 2015. Selecting (in)valid instruments for instrumental variables estimation. Available at http://www.hec.unil.ch/documents/seminars/iems/1849.pdf.

Tables

Table I: **Weights and percentiles of weighted median function**

	$\hat{\beta}_1$	$\hat{\beta}_2$	$\hat{\beta}_3$	$\hat{\beta}_4$	$\hat{\beta}_5$	$\hat{\beta}_6$	$\hat{\beta}_7$	$\hat{\beta}_8$	$\hat{\beta}_9$	$\hat{\beta}_{10}$
Simple median										
Weight (w_j)	$\frac{1}{10}$	$\frac{1}{10}$	$\frac{1}{10}$	$\frac{1}{10}$	$\frac{1}{10}$	$\frac{1}{10}$	$\frac{1}{10}$	$\frac{1}{10}$	$\frac{1}{10}$	$\frac{1}{10}$
Percentile (p_j)	5	15	25	35	45	55	65	75	85	95
Weighting 1										
Weight (w_j)	$\frac{1}{30}$	$\frac{2}{30}$	$\frac{3}{30}$	$\frac{4}{30}$	$\frac{5}{30}$	$\frac{5}{30}$	$\frac{4}{30}$	$\frac{3}{30}$	$\frac{2}{30}$	$\frac{1}{30}$
Percentile	1.67	6.67	15.00	26.67	41.67	58.33	73.33	85.00	93.33	98.33
Weighting 2										
Weight (w_j)	$\frac{2}{36}$	$\frac{3}{36}$	$\frac{10}{36}$	$\frac{8}{36}$	$\frac{5}{36}$	$\frac{3}{36}$	$\frac{2}{36}$	$\frac{1}{36}$	$\frac{1}{36}$	$\frac{1}{36}$
Percentile (p_j)	2.78	9.72	27.78	52.78	70.83	81.94	88.89	93.06	95.83	98.61

Weights and percentiles of the empirical distribution function assigned to the ordered ratio instrumental variable estimates ($\hat{\beta}_j$) for the hypothetical examples given in Figure 3.

Table II: **Results from simulation study in two-sample setting with null causal effect**

				Inverse-variance weighted		Weighted median		Penalized weighted median		MR-Egger regression	
N	Proportion of invalid IVs	F	R^2	Mean estimate (mean SE)	Power	Mean estimate (mean SE)	Power	Mean estimate (mean SE)	Power	Mean estimate (mean SE)	Power
				Scenario 1. Balanced pleiotropy, InSIDE assumption satisfied							
10 000	0.1	10.7	2.6%	-0.001 (0.114)	5.4	-0.001 (0.093)	3.2	-0.001 (0.093)	3.4	-0.003 (0.287)	6.3
10 000	0.2	10.7	2.6%	0.001 (0.153)	6.2	0.001 (0.098)	4.5	0.001 (0.098)	4.0	-0.001 (0.386)	6.2
10 000	0.3	10.7	2.6%	0.003 (0.185)	6.3	0.001 (0.103)	6.2	0.001 (0.104)	5.2	0.000 (0.467)	6.0
20 000	0.1	20.5	2.5%	-0.001 (0.107)	5.1	0.000 (0.067)	3.4	0.000 (0.067)	3.6	0.000 (0.305)	6.0
20 000	0.2	20.5	2.5%	0.002 (0.150)	5.3	0.001 (0.071)	4.4	0.001 (0.071)	4.4	-0.004 (0.426)	6.1
20 000	0.3	20.5	2.5%	-0.004 (0.184)	5.7	-0.001 (0.075)	6.4	-0.001 (0.077)	6.3	-0.004 (0.523)	6.2
				Scenario 2. Directional pleiotropy, InSIDE assumption satisfied							
10 000	0.1	10.7	2.6%	0.126 (0.111)	14.6	0.033 (0.093)	4.9	0.024 (0.093)	4.2	0.013 (0.279)	6.3
10 000	0.2	10.7	2.6%	0.256 (0.145)	37.0	0.078 (0.100)	10.7	0.071 (0.102)	9.6	0.037 (0.363)	6.5
10 000	0.3	10.7	2.6%	0.384 (0.169)	62.7	0.139 (0.109)	21.8	0.149 (0.114)	22.1	0.046 (0.421)	6.3
20 000	0.1	20.5	2.5%	0.134 (0.104)	15.0	0.026 (0.067)	4.9	0.026 (0.068)	5.2	0.003 (0.295)	6.1
20 000	0.2	20.5	2.5%	0.271 (0.141)	42.9	0.061 (0.072)	11.9	0.080 (0.078)	15.8	0.011 (0.398)	6.2
20 000	0.3	20.5	2.5%	0.404 (0.166)	70.4	0.115 (0.080)	25.4	0.177 (0.095)	35.9	0.016 (0.467)	6.0
				Scenario 3. Directional pleiotropy, InSIDE assumption not satisfied							
10 000	0.1	13.5	3.3%	0.182 (0.092)	48.0	0.145 (0.095)	29.9	0.062 (0.094)	12.6	0.363 (0.195)	50.9
10 000	0.2	16.3	3.9%	0.318 (0.105)	77.2	0.303 (0.097)	61.3	0.186 (0.097)	37.9	0.555 (0.204)	72.5
10 000	0.3	19.2	4.6%	0.421 (0.110)	91.1	0.435 (0.092)	82.5	0.335 (0.095)	65.9	0.651 (0.204)	83.2
20 000	0.1	26.0	3.1%	0.189 (0.084)	53.5	0.131 (0.072)	32.4	0.059 (0.070)	13.2	0.412 (0.184)	57.5
20 000	0.2	31.7	3.8%	0.327 (0.100)	81.0	0.290 (0.075)	63.8	0.176 (0.077)	40.5	0.607 (0.198)	77.1
20 000	0.3	37.2	4.4%	0.427 (0.105)	93.5	0.428 (0.072)	83.9	0.321 (0.077)	68.4	0.697 (0.197)	86.9

Mean estimates, mean standard errors, and power of 95% confidence interval to reject null hypothesis of inverse-variance weighted, weighted median, and MR-Egger regression methods in simulation study for two-sample Mendelian randomization with a null ($\beta = 0$) causal effect.

Abbreviations: IV, instrumental variable; SE, standard error

Table III: **Results from simulation study in two-sample setting with positive causal effect**

				Inverse-variance weighted		Weighted median		Penalized weighted median		MR-Egger regression	
N	Proportion of invalid IVs	F	R^2	Mean estimate (mean SE)	Power	Mean estimate (mean SE)	Power	Mean estimate (mean SE)	Power	Mean estimate (mean SE)	Power
				Scenario 1. Balanced pleiotropy, InSIDE assumption satisfied							
10 000	0.1	10.7	2.6%	0.090 (0.116)	16.2	0.085 (0.098)	12.3	0.086 (0.098)	12.4	0.049 (0.292)	6.7
10 000	0.2	10.7	2.6%	0.092 (0.155)	11.7	0.088 (0.103)	13.5	0.089 (0.103)	12.7	0.052 (0.390)	6.5
10 000	0.3	10.7	2.6%	0.094 (0.186)	9.4	0.088 (0.109)	13.4	0.089 (0.109)	12.8	0.053 (0.470)	6.4
20 000	0.1	20.5	2.5%	0.095 (0.108)	22.1	0.092 (0.071)	24.0	0.093 (0.071)	24.2	0.064 (0.309)	6.8
20 000	0.2	20.5	2.5%	0.097 (0.150)	13.7	0.093 (0.075)	24.4	0.094 (0.075)	24.1	0.060 (0.428)	6.5
20 000	0.3	20.5	2.5%	0.092 (0.184)	9.6	0.091 (0.079)	22.6	0.092 (0.080)	22.7	0.061 (0.525)	6.3
				Scenario 2. Directional pleiotropy, InSIDE assumption satisfied							
10 000	0.1	10.7	2.6%	0.217 (0.114)	45.9	0.121 (0.099)	20.9	0.111 (0.099)	18.3	0.066 (0.285)	7.4
10 000	0.2	10.7	2.6%	0.348 (0.148)	68.0	0.168 (0.107)	32.5	0.160 (0.108)	28.7	0.090 (0.367)	7.3
10 000	0.3	10.7	2.6%	0.475 (0.171)	84.3	0.232 (0.116)	47.6	0.239 (0.121)	46.1	0.099 (0.425)	7.0
20 000	0.1	20.5	2.5%	0.230 (0.105)	61.4	0.120 (0.071)	37.2	0.119 (0.072)	36.0	0.067 (0.298)	7.1
20 000	0.2	20.5	2.5%	0.366 (0.143)	80.3	0.157 (0.077)	52.5	0.173 (0.082)	53.9	0.076 (0.401)	6.7
20 000	0.3	20.5	2.5%	0.500 (0.168)	92.3	0.213 (0.086)	66.3	0.269 (0.099)	72.0	0.081 (0.469)	6.4
				Scenario 3. Directional pleiotropy, InSIDE assumption not satisfied							
10 000	0.1	13.5	3.3%	0.274 (0.095)	71.1	0.238 (0.101)	48.5	0.154 (0.099)	29.1	0.432 (0.202)	55.5
10 000	0.2	16.3	3.9%	0.411 (0.107)	89.9	0.400 (0.103)	75.8	0.283 (0.103)	55.6	0.634 (0.209)	76.5
10 000	0.3	19.2	4.6%	0.515 (0.112)	96.8	0.533 (0.099)	90.5	0.433 (0.101)	78.5	0.736 (0.209)	86.9
20 000	0.1	26.0	3.1%	0.285 (0.085)	81.1	0.229 (0.076)	63.8	0.153 (0.074)	47.6	0.491 (0.189)	62.1
20 000	0.2	31.7	3.8%	0.423 (0.101)	93.4	0.391 (0.079)	85.0	0.274 (0.081)	71.1	0.694 (0.201)	81.2
20 000	0.3	37.2	4.4%	0.525 (0.106)	98.0	0.529 (0.076)	94.7	0.420 (0.082)	87.1	0.788 (0.200)	90.2

Mean estimates, mean standard errors, and power of 95% confidence interval to reject null hypothesis of inverse-variance weighted, weighted median, and MR-Egger regression methods in simulation study for two-sample Mendelian randomization with a positive ($\beta = 0.1$) causal effect.

Table IV: **Results from applied example**

Analysis method	All genetic variants Estimate (SE)	p-value [1]	Primary association with target exposure Estimate (SE)	p-value [1]
Low-density lipoprotein cholesterol (LDL-c)				
Inverse-variance weighted	0.482 (0.060)	***	0.470 (0.055)	***
Simple median	0.429 (0.070)	***	0.429 (0.079)	***
Weighted median	0.458 (0.065)	***	0.457 (0.065)	***
Penalized weighted median	0.457 (0.063)	***	0.457 (0.067)	***
MR-Egger regression: slope	0.617 (0.103)	***	0.562 (0.094)	***
intercept	-0.009 (0.005)		-0.006 (0.005)	
High-density lipoprotein cholesterol (HDL-c)				
Inverse-variance weighted	-0.254 (0.070)	***	-0.137 (0.066)	*
Simple median	-0.267 (0.090)	**	-0.224 (0.085)	**
Weighted median	-0.069 (0.071)		-0.066 (0.065)	
Penalized weighted median	-0.071 (0.068)		-0.064 (0.066)	
MR-Egger regression: slope	-0.013 (0.115)		0.092 (0.107)	
intercept	-0.014 (0.005)	*	-0.013 (0.005)	*
Triglycerides				
Inverse-variance weighted	0.416 (0.081)	***	0.417 (0.095)	**
Simple median	0.512 (0.101)	***	0.565 (0.105)	***
Weighted median	0.516 (0.084)	***	0.521 (0.087)	***
Penalized weighted median	0.528 (0.078)	***	0.539 (0.089)	***
MR-Egger regression: slope	0.422 (0.140)	**	0.464 (0.155)	**
intercept	-0.000 (0.008)		-0.004 (0.009)	

[1]p-values are indicated as: * = $p < 0.05$, ** = $p < 0.01$, *** = $p < 0.0001$

Estimates (standard errors) of causal effects of lipid fractions on coronary artery disease risk. Estimates are log odds ratios per 1 standard deviation increase in the exposure. The intercept term in MR-Egger regression provides a test of directional pleiotropy.

Table V: **Summary of methods considered in this paper**

Method	Breakdown	IV2	IV3	Comments
Two-stage least squares	0%	✗	✗	Requires individual-level data. Biased when at least 1 genetic variant is an invalid IV.
Inverse-variance weighted (IVW)	0%	✗	✗	Equivalent to two-stage least squares method with summary data. Also biased when at least 1 genetic variant is an invalid IV.
Simple median	50%	✓	✓	Consistent when 50% of genetic variants are valid IVs. Inefficient compared with IVW and weighted median methods.
Weighted median	50%	✓	✓	Consistent when 50% of weight contributed by genetic variants is valid. Efficiency is similar to that of IVW method.
Penalized weighted median	50%	✓	✓	Equivalent to weighted median when there is no causal effect heterogeneity. Downweights the contribution of heterogeneous variants, so may have better finite sample properties, particularly if there is directional pleiotropy.
MR-Egger regression	100%	✗	✓	Consistent when 100% of genetic variants are invalid, but requires variants to satisfy a weaker assumption (the InSIDE assumption). This assumption is not automatically violated by an association between a genetic variant and a confounder, but it would be violated if several variants were associated with the same confounder. Substantially less efficient than IVW and median-based methods, and more susceptible to weak instrument bias in a one-sample setting.

Breakdown refers to the breakdown level, the proportion of information that can come from invalid instrumental variables (IVs) before the method gives biased estimates. IV2 and IV3 refer to whether violations of the second (no association with confounders) and third (no direct effect on the outcome) instrumental variable assumptions are allowed (✓) or not allowed (✗).
